# PhotoGEA: An R Package for Closer Fitting of Photosynthetic Gas Exchange Data With Non‐Gaussian Confidence Interval Estimation

**DOI:** 10.1111/pce.15501

**Published:** 2025-03-30

**Authors:** Edward B. Lochocki, Coralie E. Salesse‐Smith, Justin M. McGrath

**Affiliations:** ^1^ Carl R. Woese Institute for Genomic Biology University of Illinois Urbana–Champaign Urbana Illinois USA; ^2^ Plant Biology Department University of Illinois Urbana–Champaign Urbana Illinois USA; ^3^ USDA ARS Global Change and Photosynthesis Research Unit Urbana Illinois USA

**Keywords:** C_3_ and C_4_ *A*‐*C*_*i*_ curve fitting, estimating mesophyll conductance, estimating *V*_*c*max_, photosynthetic gas exchange

## Abstract

Fitting mechanistic models, such as the Farquhar‐von‐Caemmerer‐Berry model, to experimentally measured photosynthetic CO_2_ response curves (*A*‐*C*_*i*_ curves) is a widely used technique for estimating the values of key leaf biochemical parameters and determining limitations to photosynthesis in vivo. Here, we present *PhotoGEA*, an *R* package with tools for C_3_ *A*‐*C*_*i*_, C_3_ Variable *J* and C_4_ *A*‐*C*_*i*_ curve fitting. In contrast to existing software, these automated tools use derivative‐free optimizers to ensure close fits and they calculate non‐Gaussian confidence intervals to indicate which parameter values are most reliable. Results from *PhotoGEA*'s C_3_ *A*‐*C*_*i*_ curve fitting tool are compared against other available tools, where it is found to achieve the closest fits and most reasonable parameter estimates across a range of curves with different characteristics. *PhotoGEA*'s C_3_ Variable *J* and C_4_ *A*‐*C*_*i*_ fitting tools are also presented, demonstrating how they can provide insights into mesophyll conductance and the processes limiting C_4_ photosynthesis at high CO_2_ concentrations. *PhotoGEA* enables users to develop data analysis pipelines for efficiently reading, processing, fitting and analysing photosynthetic gas exchange measurements. It includes extensive documentation and example scripts to help new users become proficient as quickly as possible.

## Summary statement

Fitting mechanistic models to gas exchange measurements is a key technique in plant physiology for understanding limitations to photosynthesis. *PhotoGEA* is a new *R* package for fitting C_3_ and C_4_ CO_2_ response curves and its unique approach ensures close fits and useful uncertainty metrics.

## 1 Introduction

Vascular land plants emit water vapour through transpiration, absorb CO_2_ during the Calvin‐Benson‐Bassham cycle, emit O_2_ via the light‐dependent reactions and emit CO_2_ through photorespiration and several other biochemical pathways. These gas exchange processes are critical in determining plant growth, water use and nutrient uptake and even influence the molecular and isotopic composition of the atmosphere (Ainsworth and Long 2021; Yakir and Sternberg 2000). Thus, the ability to quantify rates of gas exchange plays a key role in diverse fields such as plant science, agriculture, ecology and climate science (Mooney 1972; Evans and von Caemmerer 2013; Haworth et al. 2018; Baldocchi 2020).

In a plant physiology context, leaf‐level CO_2_ response curves, where the net CO_2_ assimilation rate ($A_{n}$) and the intercellular CO_2_ concentration ($C_{i}$) are recorded while varying the CO_2_ concentration surrounding a leaf, are one of the most important gas exchange measurements because they enable estimates of key biochemical parameters and the identification of rate‐limiting processes (Long 2003; Busch et al. 2024). These curves are commonly referred to as *A*‐*C*_*i*_ curves, or, when also using chlorophyll fluorescence (CF) to record the operating efficiency of photosystem II ($\phi_{\mathrm{PSII}}$), *A*‐*C*_*i*_ + CF curves. Parameter estimates are made by fitting a biochemical model of leaf‐level CO_2_ assimilation to experimentally measured curves, such as the Farquhar‐von‐Caemmerer‐Berry (FvCB) model for C_3_ plants (Farquhar et al. 1980; Busch et al. 2018; Lochocki and McGrath 2025) or the von Caemmerer model for C_4_ plants (von von Caemmerer 2000; 2021).

Measuring and fitting CO_2_ response curves has provided a great deal of insight into photosynthetic biochemistry at several scales and contexts. Key results include an understanding of how capacities for light harvesting and CO_2_ fixation are coordinated with each other (Wullschleger 1993) and dependent on nutrient availability (Kattge et al. 2009; Walker et al. 2014), temperature (Kattge and Knorr 2007; Kumarathunge et al. 2019) and atmospheric CO_2_ concentration (Bernacchi et al. 2005) across diverse C_3_ plant species. Parameters estimated from response curves have been used to calibrate high‐throughput phenotyping methods based on hyperspectral reflectance (Heckmann et al. 2017; Yendrek et al. 2017; Meacham‐Hensold et al. 2019) or satellite data (Croft et al. 2017), and as inputs to mechanistic crop growth models (Lochocki et al. 2022; Wu 2023). Curve fitting has revealed natural variation in photosynthetic biochemistry within a single species (De Souza et al. 2020) and has been used to characterize engineered plants with improved water use or photosynthetic efficiency (Caine et al. 2019; Dunn et al. 2019; De Souza et al. 2022; Salesse‐Smith, Lochocki, et al. 2024).

This important work has been facilitated by software tools for fitting CO_2_ response curves, which have helped make this technique more accessible to researchers without programming expertise (Busch et al. 2024). These tools span a wide range of methods, and they can be categorized broadly by biological system and measurement type (C_3_ or C_4_ *A*‐*C*_*i*_ with or without CF), software platform (Excel spreadsheet, R package, or other), approach to identifying rate‐limiting processes and determining parameter uncertainties, and type of numerical optimization algorithm. Some of the earliest fitting tools were Excel spreadsheets, a format that revolutionized the fitting process due to its ease of operation and remains popular today (Sharkey et al. 2007). However, spreadsheet tools require frequent manual operations, preventing automation, and it is difficult to deliver updates or bug fixes to spreadsheet users, among other potential issues (Stinziano et al. 2021). As an alternative, software packages designed for the *R* language (R Core Team 2024) have been available for the last decade (Duursma 2015), enabling command line interfaces for automation and a robust distribution system for updates, but also requiring more coding skills to operate. There are also options available in other software languages such as *SAS* code (Dubois et al. 2007), or via online services (Gu et al. 2010).

All these curve fitting tools perform two central functions—identifying the rate‐limiting process at each point in the curve, and estimating the values of key parameters. There are three main approaches to identifying limiting processes: manual, exhaustive and full‐curve. In manual fitting tools, users assign a rate‐limiting process to each point before estimating parameter values (Sharkey et al. 2007). Exhaustive fitting tools can be understood as an extension of manual tools where an algorithm automatically tests all possible ways to assign limiting processes to each point. The set of assignments that produces the smallest sum of squared residuals is chosen and used for the final parameter estimates (Gu et al. 2010). In full‐curve tools, identification of limiting processes and estimation of parameter values proceed simultaneously (Dubois et al. 2007), where the rate‐limiting process at each point is determined from the parameter values according to an equation that incorporates each potential process. There are also three main approaches to parameter estimation, where values can be estimated using ordinary least squares regression, nonlinear regression with quasi‐Newton optimization, or nonlinear regression with derivative‐free optimization. Besides best estimates of parameter values, tools can quantify uncertainties by assuming a Gaussian probability distribution or by using non‐Gaussian approaches such as likelihood ratio confidence intervals (Pek et al. 2017; Doganaksoy 2021), although some tools do not calculate uncertainties. Finally, tools can vary in the model equations they use; for example, not all tools for fitting C_3_ CO_2_ response curves include triose phosphate utilization (TPU) limitations, although considering TPU is necessary for accurate parameter estimates (Gregory et al. 2021).

In principle, each approach described above can produce close fits and accurate parameter estimates, but each may have different drawbacks or advantages. Moreover, even fitting tools employing the same central approach may produce different results due to differences in the details of their code implementations. Thus, despite the diversity of existing software, there is still room for new tools and fitting approaches, especially regarding full‐curve limitation identification, derivative‐free optimizers and non‐Gaussian uncertainty estimates, which have not been widely employed. Among existing peer‐reviewed tools for fitting C_3_ *A*‐*C*_*i*_ curves that include TPU limitations (Sharkey et al. 2007; Duursma 2015; Sharkey 2016; Stinziano et al. 2021; Gregory et al. 2021), none provide these features (Table 1), and we argue later that these features add substantial value to curve fitting analyses. Likewise, no tools for fitting C_3_ *A*‐*C*_*i*_ + CF curves with the ‘Variable J’ method that include TPU limitations (Moualeu‐Ngangue et al. 2017) or for fitting C_4_ *A*‐*C*_*i*_ curves (Bellasio et al. 2016; Zhou et al. 2019) provide them either. Another issue among existing tools is that none of the available *R* packages include fully worked examples starting from instrument log files, posing a barrier to some researchers who wish to use them.

**Table 1:** Overview of peer‐reviewed tools for fitting C_3_ *A‐C_i_* curves that include triose phosphate utilization (TPU) limitations.

Name	Platform	Limitation identification	Optimization algorithm	Uncertainty estimate^a^	TPU options^b^		
					$\alpha_{\mathrm{old}}$	$\alpha_{G}$ and $\alpha_{S}$	$\alpha_{T}$
*PCE calculator*	Excel spreadsheet	Manual	Quasi‐Newton	None	Fit	N/A	N/A
*plantecophys*	R package	Exhaustive	Ordinary least squares	Gaussian SE	Fixed	N/A	N/A
*photosynthesis*	R package	Exhaustive	Ordinary least squares	Gaussian SE	Fixed	N/A	N/A
*msuRACiFit*	R package	Full curve	Quasi‐Newton	None	N/A	Fit	N/A
*PhotoGEA*	R package	Full curve	Derivative‐free	Likelihood ratio CI	Fit	Fit	Fit

*Note:* Versions and other specifications for the tools are provided in Section 2.4. *α*_old_ is the fraction of remaining glycolate carbon not returned to the chloroplast after accounting for carbon released as CO_2_, typically denoted *α* in older publications. *α_G_, α_S_* and *α_T_* are the fractions of glycolate carbon remaining in the cytosol as glycine, serine and 5,10‐methylene‐tetrahydrofolate (CH_2_‐THF), respectively. Fit: the parameter can be fit. Fixed: the parameter value can be set by the user but cannot be fit. N/A: the parameter is not used in the package's equations, and is therefore effectively fixed to a value of zero.

^a^ CI, confidence interval; SE, standard error.

^b^ The TPU options use nomenclature from Busch et al. (2018) and Busch (2020).

Here, we present an *R* package called *PhotoGEA* (photosynthetic gas exchange analysis) with tools for fitting C_3_ *A*‐*C*_*i*_, C_3_ *A*‐*C*_*i*_ + CF and C_4_ *A*‐*C*_*i*_ curves that provide unique features not found in other software. We show that *PhotoGEA*'s C_3_ *A*‐*C*_*i*_ fitting tool produces results that more closely fit tobacco *A*‐*C*_*i*_ curves than other tools, and that its non‐Gaussian confidence interval calculations accurately identify unreliable parameter estimates. We also apply *PhotoGEA*'s Variable *J* fitting tool to a set of soybean *A*‐*C*_*i*_ + CF curves, and present *PhotoGEA*'s C_4_ *A*‐*C*_*i*_ fitting tool using a set of maize and sorghum *A*‐*C*_*i*_ curves. *PhotoGEA* helps facilitate other tasks related to curve fitting, such as reading instrument log files, validating data and synthesizing results, and has already proven useful in several plant physiology studies (Salesse‐Smith, Lochocki, et al. 2024; Salesse‐Smith, Adar, et al. 2025; Pelech et al. 2025).

## 2 Methods

### 2.1 Brief Overview of *PhotoGEA*

The *PhotoGEA* package contains three core functions for fitting CO_2_ response curves using mechanistic models:

- *fit_c3_aci* for C_3_ *A‐C_i_* curves; currently able to fit $\alpha_{\mathrm{old}}$, $\alpha_{G}$, $\alpha_{S}$, $\alpha_{T}$, $\Gamma^{*}$, $g_{\mathrm{mc}}$, J, $R_{L}$, $T_{p}$ and $V_{c\max}$. By default, only $\alpha_{\mathrm{old}}$, J, $R_{L}$, $T_{p}$ and $V_{c\max}$ are fit.
- *fit_c3_variable_j* for C_3_ *A‐C_i_* + CF curves; currently able to fit $\alpha_{\mathrm{old}}$, $\alpha_{G}$, $\alpha_{S}$, $\alpha_{T}$, $\Gamma^{*}$, J, $R_{L}$, τ, $T_{p}$ and $V_{c\max}$. By default, only $\alpha_{\mathrm{old}}$, J, $R_{L}$, τ, $T_{p}$ and $V_{c\max}$ are fit.
- *fit_c4_aci* for C_4_ *A‐C_i_* curves; currently able to fit $\alpha_{\mathrm{PSII}}$, $g_{\mathrm{bs}}$, $g_{\mathrm{mc}}$, J, $R_{L}$, $f_{R_{\mathrm{Lm}}}$, $V_{c\max}$, $V_{p\max}$ and $V_{\mathrm{pr}}$. By default, only $R_{L}$, $V_{c\max}$ and $V_{p\max}$ are fit.

See Supporting Information S1: Sections S1–S2 for definitions of all parameters listed above. These functions allow users to choose temperature response parameters from preset options (or to provide their own; Supporting Information S1: Section S7), to choose which parameters to fit, and to apply constraints on the range of each parameter. Flexible temperature response options are essential for analysing curves from some species; for example, *Gossypium hirsutum* mesophyll conductance was found to follow a second‐order polynomial temperature response rather than a typical Arrhenius‐type response (Sargent et al. 2024). Each function takes a whole‐curve fitting approach using maximum likelihood regression and derivative‐free optimization algorithms, and non‐Gaussian confidence intervals for each estimated parameter value are calculated from likelihood ratios. To avoid numerical errors, *PhotoGEA*'s FvCB model code identifies limiting processes by choosing minimal carboxylation rates (Supporting Information S1: Equations A4 and A9) rather than minimal assimilation rates (Supporting Information S1: Equation A17), and only allows TPU limitations above a biochemistry‐based CO_2_ concentration threshold determined by $\Gamma^{*}$, $\alpha_{\mathrm{old}}$, $\alpha_{G}$, $\alpha_{S}$ and $\alpha_{T}$ (Supporting Information S1: Equations A3 and A8) rather than a fixed threshold such as 400 μbar (Lochocki and McGrath 2025).

A major goal of the *PhotoGEA* package is to provide a full set of tools for creating a complete gas exchange data analysis pipeline. Data analysis involves many steps, including data collection, data integration, exploratory analysis, statistical analysis and others (Tukey 1962; O'Neil and Schutt 2014). *PhotoGEA* aims to aid plant physiologists with these tasks by providing functions for directly reading instrument log files without requiring any pre‐formatting (*read_gasex_file*), for checking that the same sequence of CO_2_ set‐points were used across all curves in a data set (*check_response_curve_data*), for removing extra recovery points (*organize_response_curve_data*) and for many other common practicalities. To help ensure valid results, all *PhotoGEA* functions check and report units. *PhotoGEA* includes detailed examples with methods for visually checking the input data and the fits.

The *PhotoGEA* package is written entirely in *R* and its source code is publicly available from its GitHub page under the MIT license (Lochocki 2024). Extensive documentation, including simple examples using each function in the package and long‐form vignettes, are included with the package itself and available at the GitHub page. Users are encouraged to use the long‐form examples as a basis for their own scripts. Automated tests ensure that the package works on Windows, macOS and Ubuntu using *R* version 3.6.0 (*PhotoGEA*'s minimum supported version) and the latest version of *R* (4.4.2 at the time of writing). Regression tests are implemented using the *testthat* package (Wickham 2011), and *covr::package_coverage* (Hester 2023) reports that 90.8% of *PhotoGEA*'s code is covered by tests, examples, and vignettes. In general, *PhotoGEA* is designed to follow the principles for resilient coding described by Stinziano et al. (2021).

### 2.2 Experimental Methods

CO_2_ response curves were measured from fully developed leaves of greenhouse‐grown tobacco (cv. Samsun) plants using portable photosynthesis systems (LI‐6800) with integrated multiphase flash fluorometers (6800‐01A) and 6 cm^2^ apertures (LI‐COR Environmental). For these curves, the sequence of reference CO_2_ concentration set‐points was 400, 300, 200, 150, 100, 75, 50, 40, 30, 20, 10, 400, 400, (500), 600, 800, 1000, 1200, 1500 μmol mol^−1^, and measurements were logged after 3–5 min according to stability criteria. The point at 500 μmol mol^−1^ was used for most, but not all, curves. Chlorophyll fluorescence was also measured at each point using the multiphase flash option with a saturating flash of 10,000 μmol m^−2^ s^−1^. CO_2_ response curves were measured at incident photosynthetically active photon flux density (PPFD) of 100, 150, 200, 250, 300, 400, 450, 500, 600, 800, 1000, 1200 or 1500 μmol m^−2^ s^−1^ with leaf temperature set to 27°C and vapour pressure deficit in the leaf chamber set to 1.3 kPa. Three or four curves were measured from different plants at each incident PPFD ($Q_{\mathrm{in}}$), with the exception of 450 and 1200 μmol m^−^^2^ s^−1^ (one curve each) and 1000 and 1500 μmol m^−^^2^ s^−1^ (two curves each), for a total of 36 curves. These plants were sown in January 2023 and measured in March 2023 (winter in Champaign, Illinois). The greenhouse used supplemental lighting with a 12 h photoperiod. A sensor in the greenhouse measuring total light incident on the plants (natural and supplemental) indicates the median hourly daytime PPFD during this period was approximately 300 μmol m^−^^2^ s^−1^, and the daily maximum was below 600 μmol m^−2^ s^−1^ on most days.

CO_2_ response curves were also measured from field‐grown soybean (cv. LD11), maize (DeKalb DKC58 34R1B), and grain sorghum (DeKalb DKS38‐16) during August 2021 and 2022 using LI‐6800 systems with $Q_{\mathrm{in}}$ set to 2000 μmol m^−2^ s^−1^, leaf temperature between 30°C and 32°C, and relative humidity in the leaf chamber between 60% and 70%. The sequence of reference CO_2_ concentration set‐points was 400, 300, 200, 150, 100, 75, 50, 20, 400, 400, 600, 800, 1000, 1200, 1500, 1800 μmol mol^−1^ (soybean) or 400, 300, 200, 120, 70, 30, 10, 400, 400, 500, 600, 800, 1200 μmol mol^−1^ (maize and sorghum). Logging and fluorometer settings were identical to those used for the tobacco curves. All plants were in early reproductive stages, and fully developed sunlit leaves were chosen for measurements.

### 2.3 Computational Methods

Tobacco *A*‐*C*_*i*_ curves were fit on a $C_{i}$ basis using the *fit_c3_aci* function from *PhotoGEA* and four other tools: *PCE calculator* (Sharkey 2016), *plantecophys* (Duursma 2015), *photosynthesis* (Stinziano et al. 2021) and *msuRACiFit* (Gregory et al. 2021). Soybean *A*‐*C*_*i*_ + CF curves were fit using the *fit_c3_variable_j* function from *PhotoGEA*. All C_3_ fits used temperature response parameters from Sharkey et al. (2007), with the exception of soybean $K_{c}$, $K_{O}$ and $\Gamma^{*}$, where the Arrhenius parameters were estimated from Orr et al. (2016). When comparing C_3_ parameter estimates between tools, values are always reported at leaf temperature (27°C), since some tools do not provide values at the standard reference temperature of 25°C. Maize and sorghum *A*‐*C*_*i*_ curves were fit using the *fit_c4_aci* and *fit_c4_aci_hyperbola* functions from *PhotoGEA*. Temperature response parameters from von von Caemmerer (2021) were used for the C_4_ mechanistic fits. For all *PhotoGEA* fits, version 1.1.0 of the package was used, and best‐fit parameters were identified using the *DEoptim* evolutionary optimizer from the *DEoptim R* package (Ardia et al. 2011; Mullen et al. 2011). For detailed settings used with each package, see Supporting Information S1: Section S9.

## 3 Results

### 3.1 Comparing Fits Between *PhotoGEA* and Other Peer‐Reviewed Tools for One C_3_ *A‐C_i_* Curve

In the FvCB model for C_3_ leaves, $A_{n}$ can be limited by ribulose‐1,5 bisphosphate carboxylase/oxygenase (Rubisco) activity, ribulose‐1,5 bisphosphate (RuBP) regeneration, or TPU, and key parameters that can be estimated from a curve fit include the maximum rate of Rubisco activity ($V_{c\max}$), the potential whole‐chain electron transport rate (J), the maximum rate of TPU ($T_{p}$) and the rate of non‐photorespiratory CO_2_ release in the light ($R_{L}$). When assimilation is limited by Rubisco activity, it is typically denoted by $A_{c}$ rather than $A_{n}$; likewise, $A_{j}$ and $A_{p}$ denote rates limited by RuBP regeneration or TPU, respectively. See Supporting Information S1: Section S3 for a full description of the model equations. Key outputs from any fitting tool using this model will include the modelled values of $A_{n}$, $A_{c}$, $A_{j}$ and $A_{p}$ at each point in the curve, along with best‐fit values of $V_{c\max}$, J, $T_{p}$ and $R_{L}$ and uncertainty estimates for each parameter. If fits are made on a $C_{i}$ basis (rather than using the chloroplast CO_2_ concentration, $C_{c}$), the photosynthetic parameter estimates should be understood as ‘apparent’ values rather than true chloroplastic values (Ethier and Livingston 2004).

The Rubisco‐limited rate $A_{c}$ depends on $V_{c\max}$, so any points in a CO_2_ response curve identified as being Rubisco‐limited will determine the best‐fit estimate for $V_{c\max}$. Similarly, the points identified as being limited by RuBP regeneration or TPU will determine the estimates for J and $T_{p}$. As a corollary, if no points are identified as being Rubisco‐limited, then it is not possible to estimate $V_{c\max}$; similar considerations apply for for J and $T_{p}$. The net CO_2_ assimilation rate depends on $R_{L}$ regardless of which process is limiting. Although TPU was not part of the original FvCB model, including TPU is essential for accurate parameter estimates; otherwise, TPU‐limited points may be misattributed to RuBP regeneration limitation, skewing estimates of J (Gregory et al. 2021). TPU is particularly important for fitting curves where $A_{n}$ decreases with $C_{i}$ at high $C_{i}$. This ‘reverse sensitivity’ can be understood as a consequence of glycolate carbon remaining in the cytosol, and two different sets of equations have been developed to model this process. The newer version (Supporting Information S1: Section S1.2) includes three parameters ($\alpha_{G}$, $\alpha_{S}$ and $\alpha_{T}$) representing the amounts of glycolate carbon leaving the photorespiratory pathway as glycine, serine, or 1‐5, methylase‐tetrahydrofolate (CH_2_‐THF), respectively (Busch et al. 2018; Busch 2020). The original version (Supporting Information S1: Section S1.1) includes a single parameter ($\alpha_{\mathrm{old}}$, originally denoted α) representing the amount of glycolate carbon leaving the photorespiratory pathway in any of these forms (Harley et al. 1992; von Caemmerer 2000). In practice, C_3_ *A*‐*C*_*i*_ curves often exhibit TPU‐limited assimilation, including reverse sensitivity, so fitting tools that do not include this process have limited utility.

**Figure 1:**
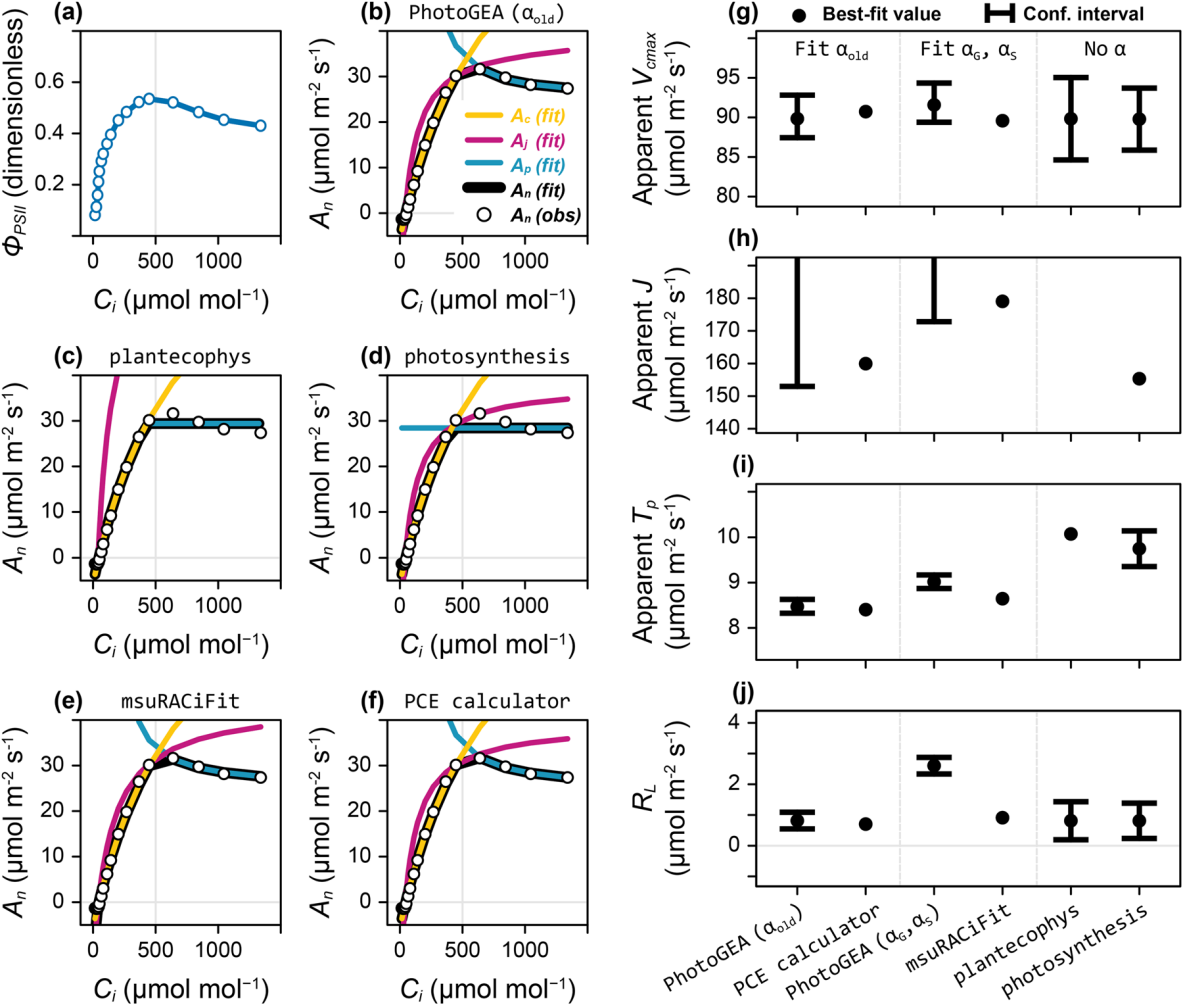
Fitting a single C_3_ *A‐C_i_* curve (designated ‘800 – wt‐5 – mcgrath1’) with several software tools. (a) $\phi_{\mathrm{PSII}}$ versus $C_{i}$ measured simultaneously with gas exchange. (b–f) Fits of $A_{n}$ versus $C_{i}$ made using *PhotoGEA* (allowing $\alpha_{\mathrm{old}}$ to vary), *plantecophys*, *photosynthesis*, *msuRACiFit* and *PCE calculator*, respectively. All lines in (a–f) are composed of straight segments connecting adjacent points and are intended only as guides to the eye. (g–j) Best‐fit values and 95% confidence intervals of apparent $V_{c\max}$, apparent J, apparent $T_{p}$, and $R_{L}$ values at leaf temperature (27°C), respectively, as determined by the fit from each tool, where results from *PhotoGEA* (allowing $\alpha_{G}$ and $\alpha_{S}$ to vary) are also included. Confidence intervals for *plantecophys* and *photosynthesis* were calculated from the standard error (SE) as best‐fit value ± 1.96 × SE.

Among available peer‐reviewed tools for fitting C_3_ *A*‐*C*_*i*_ curves that include TPU, we have selected four to compare against *PhotoGEA* (Section 2.3); these tools were chosen based on their prominence in the plant physiology literature and their ease of use, and they represent a range of approaches to curve fitting. The main goal for this comparison is to assess the broad ability of each tool to fit measured curves with a variety of limiting states and characteristics. One way to illustrate the differences between them is to fit a single curve with each tool (Figure 1). Based on CF measurements made along with this curve, assimilation is expected to be Rubisco‐limited for $C_{i}$ below 500 μmol mol^−1^ (where $\phi_{\mathrm{PSII}}$ increases with $C_{i}$) and TPU‐limited for $C_{i}$ above 500 μmol mol^−1^ (where $\phi_{\mathrm{PSII}}$ decreases with $C_{i}$), with a possible narrow region of RuBP‐regeneration limitations near 500 μmol mol^−1^, although there is no clear range where $\phi_{\mathrm{PSII}}$ is constant with $C_{i}$ (Figure 1a). Reverse sensitivity of $A_{n}$ to $C_{i}$ is evident above 500 μmol mol^−1^ (Figure 1b). This curve was measured with $Q_{\mathrm{in}}$ = 800 μmol m^−2^ s^−1^, which is much brighter than the typical light experienced by this leaf during its development, so it may be expected that few or none of the measured points are limited by RuBP regeneration (Sharkey 2019). These features may pose a challenge to fitting tools, and any estimated values of J will likely be particularly unreliable.

The curve fit from *PCE calculator* (Figure 1f and Supporting Information S1: Table S1), a manual spreadsheet tool, follows the reasoning above; all points where $C_{i}$ is below 400 μmol mol^−1^ are identified as Rubisco‐limited ($A_{n}=A_{c}$) and all points for $C_{i}$ above 500 μmol mol^−1^ are identified as TPU‐limited ($A_{n}=A_{p}$), with a single point at $C_{i}$ ≈ 450 μmol mol^−1^ identified as RuBP‐regeneration‐limited ($A_{n}=A_{j}$). The presence of an RuBP‐regeneration‐limited point is somewhat uncertain, since assigning this point to Rubisco limitations was found to just slightly increase the sum of squared residuals from 9.572 to 9.577. The curve fit from *PhotoGEA* makes the same assignments of rate‐limiting processes as *PCE calculator* (Figure 1b and Supporting Information S1: Table S1). The curve fits from *plantecophys* and *photosynthesis* have no points where $A_{n}=A_{j}$ (Figure 1c,d and Supporting Information S1: Table S1). The *msuRACiFit* package identifies three points at low $C_{i}$ as RuBP‐regeneration‐limited but none where $C_{i}$ is near 500 μmol mol^−1^ (Figure 1e and Supporting Information S1: Table S1). While the fitted values of $A_{n}$ from *msuRACiFit* are similar to those from *PhotoGEA* and *PCE calculator*, the *plantecophys* and *photosynthesis* packages are unable to capture the reverse sensitivity at high $C_{i}$ because they do not fit $\alpha_{\mathrm{old}}$, $\alpha_{G}$, $\alpha_{S}$ or $\alpha_{T}$.

Parameter estimates vary between the curve fitting tools (Figure 1g–j), where differences in best‐fit values can be attributed to differences in fitting approaches, TPU model equations, and code implementation. *PhotoGEA* is the only tool that returns a confidence interval for each estimated parameter. Major differences occur in the apparent J values (Figure 1h). *PhotoGEA* indicates a lower confidence limit but no best‐fit value or finite upper confidence limit for apparent J (Figure 1b). An infinite upper confidence limit means that J can be increased indefinitely without negatively impacting the fit quality; in other words, that no part of the curve unambiguously exhibits RuBP‐limited assimilation. In the fit for this curve, $A_{c}$ is just slightly higher than $A_{j}$ at the single RuBP‐limited point, so increasing J would change that point's identification to Rubisco‐limited but would not substantially reduce the fit quality. When the upper limit for a parameter is infinite, *PhotoGEA* considers its estimate to be unreliable and does not return a value. Note that this uncertainty in J was apparent in the *PCE calculator* fit, as discussed above; yet, *PCE calculator* does not quantify the uncertainty or provide a clear way to indicate whether the parameter estimate is reliable. Instead, it returns a J value just above the lower limit estimated by *PhotoGEA*. Compared to *PhotoGEA*, *msuRACiFit* identifies different points as being RuBP‐limited, but its best‐fit J value is also just above the lower limit estimated by *PhotoGEA*. Like *PhotoGEA*, the *plantecophys* tool does not return a best‐fit value for J.

Although the *photosynthesis* tool does not indicate any points where $A_{n}=A_{j}$, it nevertheless returns a best‐fit value for apparent J. This is related to a subtlety of the exhaustive and manual approaches to curve fitting, which can produce ‘inadmissible fits’ where ‘there is a contradiction between the limitation states designated in advance and the limitation states calculated with the optimized parameters’ (Gu et al. 2010). For this curve, the fit from *photosynthesis* designates points where $C_{i}$ lies between 543 and 741 μmol mol^−1^ as being RuBP‐regeneration‐limited, but the points in this range have $A_{n}=A_{p}$ (Supporting Information S1: Section S1.4). Such ‘inadmissible fits’ cannot occur when using a full‐curve fitting method since the limiting states are always determined from the parameter values themselves in this approach. Although inadmissible fits can be detected and rejected when using the exhaustive fitting method (Gu et al. 2010), implementations of this method do not always do this.

### 3.2 Comparing Fits Between *PhotoGEA* and Other Peer‐Reviewed Tools for Many C_3_ *A‐C_i_* Curves

**Figure 2:**
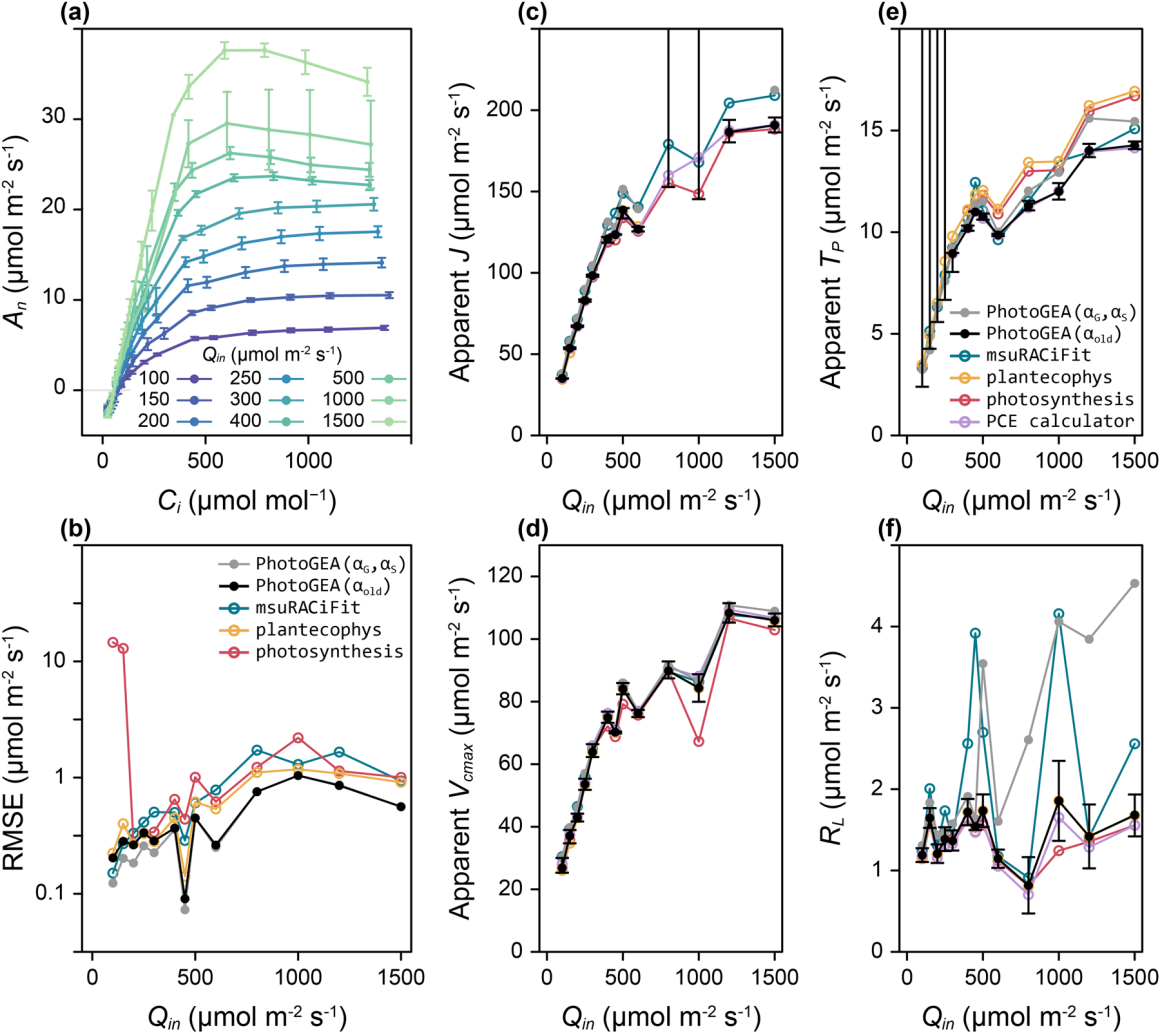
Fitting 36 C_3_ *A‐C_i_* curves measured at a range of $Q_{\mathrm{in}}$ values with several different software tools. (a) Average CO_2_ response curves at nine values of $Q_{\mathrm{in}}$. Error bars show standard error of the mean. Values of $Q_{\mathrm{in}}$ with only one *A‐C_i_* curve were excluded. (b) Average root mean square error (RMSE) values versus $Q_{\mathrm{in}}$ as calculated from each tool's fits. *PCE calculator* is excluded since the fits from this tool did not use all measured points and its RMSE values are therefore not comparable to the others. (c–f) Average best‐fit values of apparent J, apparent $V_{\mathrm{cmax}}$, apparent $T_{p}$ and $R_{L}$ at leaf temperature (27°C), respectively, versus $Q_{\mathrm{in}}$, as determined by each fitting tool. Black error bars correspond to average confidence intervals determined using *PhotoGEA* (allowing $\alpha_{\mathrm{old}}$ to vary). Some error bars have infinite upper limits and extend past the panel. Each point in (b–f) is an average value from 1 to 4 response curve fits. Statistical error bars are excluded for clarity, since they are similar across all fitting tools. [Color figure can be viewed at wileyonlinelibrary.com]

Estimating parameters from a set of CO_2_ response curves measured at different $Q_{\mathrm{in}}$ provides a more comprehensive way to compare the fitting tools (Figure 2 and Supporting Information S1: Figures S1–S9). At low $Q_{\mathrm{in}}$, RuBP regeneration is the main limiting factor across most values of $C_{i}$, and as $Q_{\mathrm{in}}$ increases, more points become limited by Rubisco activity or TPU (Figure 2a). Thus, this type of data set poses a challenge to fitting tools, which must be able to identify rate‐limiting factors across a set of curves that may each include different factors. One way to characterize the curve fits is by calculating the root mean square error (RMSE), defined by

(1) $$\mathrm{RMSE}=\sqrt{\frac{1}{N}\sum_{k=1}^{N}{\left(A_{\mathrm{obs}}^{k}-A_{\mathrm{mod}}^{k}\right)}^{2}},$$

where $A_{\mathrm{obs}}^{k}$ and $A_{\mathrm{mod}}^{k}$ are the kth observed and modelled values of $A_{n}$ in a response curve with N points. Smaller values of RMSE indicate a closer agreement between the measured and fitted assimilation rates. Across all measured curves, *PhotoGEA* generally produces the smallest RMSE values (Figure 2b), though each fitting tool produces a similar RMSE. However, the fits from *photosynthesis* at the two lowest light levels have a much larger RMSE, exceeding the RMSE from *PhotoGEA* by 10–100 times. Similar results are found when comparing values of the Akaike information criterion (AIC), a related fit quality indicator that accounts for the number of free parameters in each fit (Supporting Information S1: Figure S6).

The estimated parameter values from each tool can also be compared (Figure 2c–f), where estimates from *PhotoGEA (* $\alpha_{G}$,$\alpha_{S}$ *)* and *msuRACiFit* should be distinguished from the other tools because they use a different version of the FvCB model equations. Although estimates of apparent J from each tool show a similar dependence on $Q_{\mathrm{in}}$, demonstrating the well‐known hyperbolic relationship (von Caemmerer 2000), some differences are evident (Figure 2c). When fitting $\alpha_{\mathrm{old}}$, *PhotoGEA* indicates that J cannot be estimated reliably for any curves with $Q_{\mathrm{in}}$ of 800–1000 μmol m^−2^ s^−1^ and only returns a lower confidence limit in this range, as shown previously for one curve (Figure 1). Values estimated using *PCE calculator* and *photosynthesis* agree well with those from *PhotoGEA (* $\alpha_{\mathrm{old}}$ *)*, with the exception of curves where *PhotoGEA (* $\alpha_{\mathrm{old}}$ *)* determined that a reliable estimate could not be made. The *plantecophys* tool only provided J estimates for curves with $Q_{\mathrm{in}}$ below 800 μmol m^−2^ s^−1^ (Supporting Information S1: Figure S8). When fitting $\alpha_{G}$ and $\alpha_{S}$, *PhotoGEA* indicates that J cannot be estimated reliably for any curves with $Q_{\mathrm{in}}$ of 800–1200 μmol m^−2^ s^−2^; estimates outside this range are in good agreement with those from *msuRACiFit*, which returned an estimated value for every curve.

Each tool was able to estimate apparent $V_{c\max}$ from each curve, and the estimates from each tool have a similar $Q_{\mathrm{in}}$ dependence as the J estimates, where $V_{c\max}$ increases with $Q_{\mathrm{in}}$ (Figure 2d). Across all values of $Q_{\mathrm{in}}$, the estimated values from *PhotoGEA (* $\alpha_{\mathrm{old}}$ *)*, *PCE calculator* and *plantecophys* are nearly identical, and values from *photosynthesis* only diverge at a few points. Estimates from *PhotoGEA (* $\alpha_{G}$,$\alpha_{S}$ *)* and *msuRACiFit* also agree well.

A similar trend with $Q_{\mathrm{in}}$ was again observed for estimated values of $T_{p}$ (Figure 2e). Estimates from *PhotoGEA (* $\alpha_{\mathrm{old}}$ *)* and *PCE calculator* are nearly identical and neither indicate any TPU limitations for $Q_{\mathrm{in}}$ below 300 μmol m^−2^ s^−1^. In contrast, the *plantecophys*, *PhotoGEA (* $\alpha_{G}$,$\alpha_{S}$ *)* and *msuRACiFit* tools returned $T_{p}$ estimates for all curves, while *photosynthesis* did not indicate TPU limitations for $Q_{\mathrm{in}}$ below 450 μmol m^−2^ s^−1^ (with one exception at $Q_{\mathrm{in}}$ = 100 μmol m^−2^ s^−1^) (Supporting Information S1: Figure S9). The *plantecophys* or *photosynthesis* tools overestimate $T_{p}$ as compared to the other packages, likely because they do not fit $\alpha_{\mathrm{old}}$, $\alpha_{G}$, $\alpha_{S}$ or $\alpha_{T}$.

Estimated values of $R_{L}$ show no clear trend with $Q_{\mathrm{in}}$, although differences in magnitude are apparent between the packages (Figure 2f). Values from *PhotoGEA (* $\alpha_{\mathrm{old}}$ *)*, *PCE calculator*, *plantecophys* and *photosynthesis* are generally similar, but values from *PhotoGEA (* $\alpha_{G}$,$\alpha_{S}$ *)* and *msuRACiFit* are much higher than those from other packages and do not agree well with each other.

### 3.3 The *PhotoGEA* Approach to Fits and Confidence Intervals

*PhotoGEA* uses maximum likelihood regression with derivative‐free optimizers, a reliable fitting method that facilitates the calculation of useful non‐Gaussian confidence intervals from likelihood ratios (Doganaksoy 2021). The likelihood (L) can be thought of as the probability of observing a set of measured data, assuming a particular model parameterization (P) and level of measurement noise (σ). In maximum likelihood regression, P is varied to maximize L, enabling best estimates of the parameter values. The best‐fit parameter estimates from maximum likelihood regression do not depend on σ and always agree with the best‐fit parameters from least squares regression, a process that minimizes the sum of squared residuals (SSR). Details of confidence interval calculations are provided in the Supplemental Information (Supporting Information S1: Section S8).

**Figure 3:**
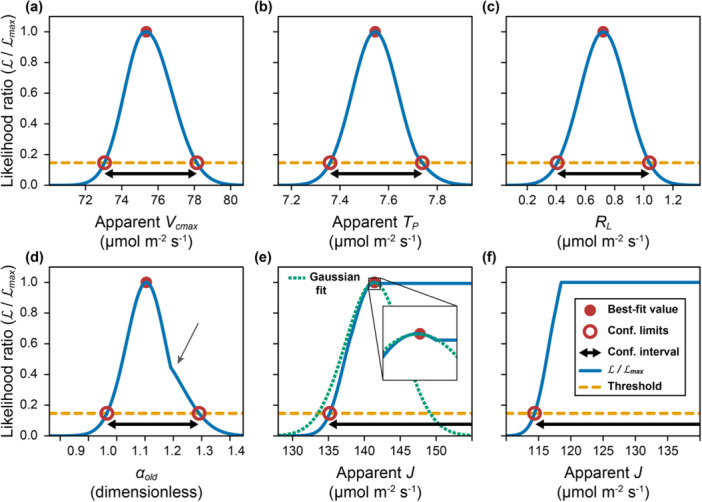
Determining confidence intervals from the likelihood ratio ($L/L_{\max}$). (a–e) Likelihood ratio as each of apparent $V_{c\max}$, apparent $T_{p}$, $R_{L}$, $\alpha_{\mathrm{old}}$ and apparent J vary around their best‐fit values, as determined by *PhotoGEA* for the curve in Figure 1 (designated ‘800 – wt‐5 – mcgrath1’). The arrow in (d) shows a ‘kink’ where the likelihood deviates from a Gaussian distribution. The dotted line in (e) shows a Gaussian fit to the likelihood ratio near the peak. The likelihood ratio confidence interval extends to infinity but the Gaussian interval would only extend to approximately 149 μmol m^−2^ s^−1^. Inset shows an expanded version of the likelihood ratio and fit near the peak. (f) Likelihood ratio versus apparent J for another curve (designated ‘1000 – wt‐1 – mcgrath1’). [Color figure can be viewed at wileyonlinelibrary.com]

In the context of CO_2_ response curve fitting, the dependence of L (or SSR) on P can take a variety of complicated shapes that can pose issues for optimization algorithms (Figure 3). Along some dimensions, there may be a shape with a clear peak, which occurs for all parameters (Figure 3a–e) when fitting the curve shown in Figure 1. However, the likelihood can also take its maximum value along a wide range of values, as shown for apparent J when fitting a different curve (Figure 3f). This happens when the corresponding rate‐limiting process is not evident, and this situation is particularly challenging for optimizers employing quasi‐Newton or gradient descent methods, which use the slope of the line to determine which direction to proceed. When there are regions of parameter space where the gradient of L or SSR is zero, multiple directions appear the same. This issue, coupled with the potential discontinuities and multiple minima, present challenges for these algorithms and may prevent them from producing close fits. On the other hand, derivative‐free optimizers do not rely on the slope and are better able to handle these situations. In the case where maximum likelihood occurs for a range of parameter values, such optimizers will report an arbitrary value from the acceptable range. For example, for the scenario in Figure 3f, the optimizer returned J ≈ 960 μmol m^−2^ s^−1^, but any value greater than approximately 120 μmol m^−2^ s^−1^ would have also maximized the likelihood.

*PhotoGEA*'s fitting functions are able to identify such arbitrary parameter values by calculating likelihood ratio confidence intervals (Supporting Information S1: Section S8). In *PhotoGEA*'s fitting procedure, σ is initialized to a value of one, and this value is used to find the best‐fit values of the parameters P. The value of σ does not affect the best‐fit estimation of P, so this choice is arbitrary. Then the RMSE of the best fit (Equation 1) is used as an estimate of σ, enabling the calculation of true likelihood values. Finally, each parameter is varied independently to find the range of values where the likelihood remains above 14.7% of its highest value ($L_{\max}$). For parameters where the likelihood takes a Gaussian shape (Figure 3a–c), relative likelihood confidence intervals calculated with this threshold closely approximate 95% confidence intervals (Rossi 2018). Note that this method for calculating confidence intervals only relies on likelihood calculations, and is therefore independent of the optimizer used to identify best‐fit parameters. In other words, derivative‐free full‐curve fitting and likelihood ratio confidence interval calculations are independent features, and, in principle, likelihood ratio confidence intervals could be combined with gradient‐based fitting approaches.

This approach becomes especially valuable when one or more rate‐limiting process is not clearly evident in a curve (Figure 3d,e). In this case, the upper limit of the confidence interval for the related parameter extends to infinity, indicating that the best‐fit value is arbitrary or otherwise not reliable and that the limitation may not be present (Figure 1h). When *PhotoGEA* identifies a confidence interval with no upper limit, the corresponding parameter is set to *NA* (‘not available’) to indicate that its value could not be reliably estimated. In tests of simulated C_3_ *A*‐*C*_*i*_ curves, this ability greatly reduces *PhotoGEA*'s rate of ‘false positives’ as compared to the other fitting tools (Supporting Information S1: Section S10). Users can disable this behaviour if desired.

When fitting CO_2_ response curves, such non‐Gaussian confidence intervals are more reliable than ones calculated by assuming Gaussian distributions. Standard errors, as returned by the widely‐used *R* functions *lm*, *nlm* and *nls*, are calculated from the Hessian matrix (i.e., the curvature) of the SSR around the best‐fit value, and are based on an assumption that the likelihood follows a Gaussian distribution. This assumption is often violated in photosynthetic response curve fitting (Figure 3d–f). For example, the curvature near the best‐fit value may be undefined, preventing an uncertainty estimate altogether (Figure 3f). The curvature may also be misleading; estimating a standard error from the curvature is equivalent to assuming a Gaussian likelihood shape, and can cause inaccurate estimates of the true confidence interval (Figure 3e). In tests of simulated C_3_ *A*‐*C*_*i*_ curves, *PhotoGEA*'s non‐Gaussian confidence intervals are much more likely to contain the true parameter values than those calculated assuming Gaussian distributions (Supporting Information S1: Section S10).

### 3.4 Estimating Mesophyll Conductance by Fitting C_3_ *A‐C_i_* + CF Curves With Variable *J*

In the FvCB model, the actual whole‐chain electron transport rate ($J_{\mathrm{actual}}$) required to support a given CO_2_ assimilation rate is

(2) $$J_{\mathrm{actual}}=\left(A_{n}+R_{L}\right)\cdot \frac{4\cdot \left(C_{i}-\frac{A_{n}}{g_{\mathrm{mc}}}\right)+8\cdot \Gamma^{*}}{\left(C_{i}-\frac{A_{n}}{g_{\mathrm{mc}}}\right)-\Gamma^{*}},$$

where $g_{\mathrm{mc}}$ is the mesophyll conductance to CO_2_ diffusion and $\Gamma^{*}$ is the CO_2_ compensation point in the absence of non‐photorespiratory CO_2_ release. This relationship holds regardless of which process is limiting assimilation (Harley et al. 1992). When assimilation is limited by RuBP regeneration, $J_{\mathrm{actual}}$ takes its maximum value for a given light level and leaf temperature ($J_{\mathrm{actual}}=J$); otherwise, $J_{\mathrm{actual}}<J$. Assuming that the electron transport rate estimated from chlorophyll fluorescence measurements ($J_{F}$) is representative of the actual rate, values of $J_{\mathrm{actual}}$ can be estimated according to

(3) $$J_{\mathrm{actual}}=J_{F}=\tau \cdot Q_{\mathrm{in}}\cdot \varphi_{\mathrm{PSII}},$$

where τ is a dimensionless proportionality factor commonly expressed as τ=α⋅β, where α is the leaf absorptance and β is the fraction of light partitioned to photosystem II (often assumed to be 0.5) (Moualeu‐Ngangue et al. 2017), or as a ‘lumped’ parameter (s) that includes other aspects of electron transport (Yin et al. 2009). Equations (2) and (3) can be solved for $g_{\mathrm{mc}}$, enabling estimates of mesophyll conductance and $C_{c}$ from combined gas exchange and chlorophyll fluorescence measurements:

(4) $$g_{\mathrm{mc}}=\frac{A_{n}}{C_{i}-\frac{\Gamma^{*}\cdot \left(\tau \cdot Q_{\mathrm{in}}\cdot \varphi_{\mathrm{PSII}}+8\cdot \left(A_{n}+R_{L}\right)\right)}{\tau \cdot Q_{\mathrm{in}}\cdot \varphi_{\mathrm{PSII}}-4\cdot \left(A_{n}+R_{L}\right)}},$$

and

(5) $$C_{c}=C_{i}-\frac{A_{n}}{g_{\mathrm{mc}}}.$$

This approach is commonly referred to as the Variable *J* method following its original description (Harley et al. 1992). Independent estimates of τ can be used in Equations (4) and (5) to calculate $g_{\mathrm{mc}}$ and $C_{c}$. Alternatively, τ can be varied along with the FvCB model parameters when fitting an *A*‐*C*_*i*_ + CF curve, where Equations (4) and (5) enable simultaneous estimates of $g_{\mathrm{mc}}$, $C_{c}$, and chloroplastic values of FvCB model parameters (Moualeu‐Ngangue et al. 2017). *PhotoGEA* allows users to take either approach, where the former does not involve any fitting and can be performed with the *calculate_c3_variable_j* function, and the latter can be performed with *fit_c3_variable_j*.

**Figure 4:**
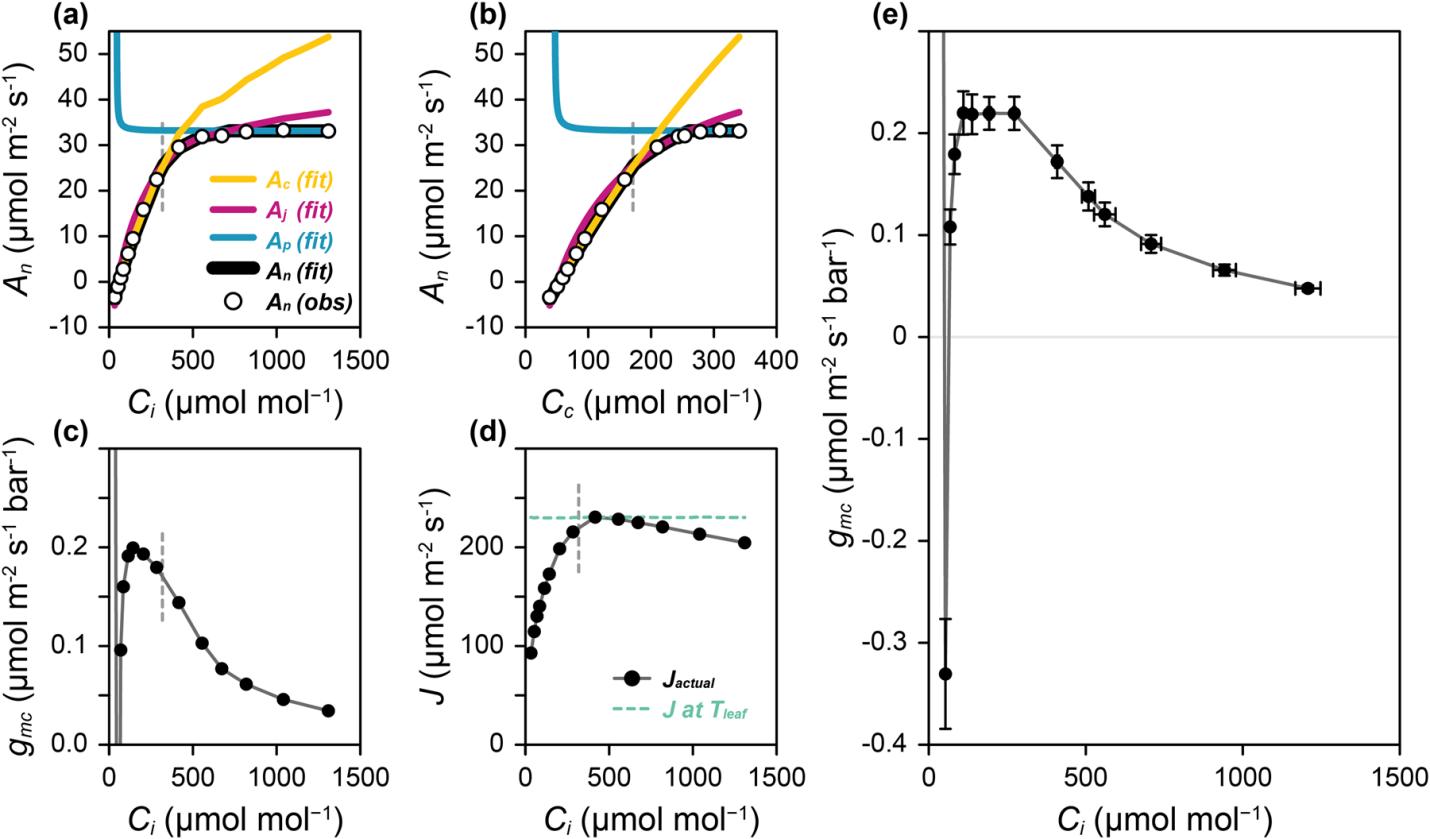
Fitting soybean *A‐C_i_* + CF curves using the *fit_c3_variable_j* function from *PhotoGEA*. (a–d) Results from one curve (designated ‘2022 – ripe2 – 4’) showing (a,b) measured and fitted assimilation rates on a $C_{i}$ and $C_{c}$ basis, (c) $g_{\mathrm{mc}}$ versus $C_{i}$, and (d) $J_{\mathrm{actual}}$ and J versus $C_{i}$. (e) Average $g_{\mathrm{mc}}$ versus $C_{i}$ from fits to eight different curves, where error bars represent the standard error. Dashed vertical lines indicate the approximate $C_{i}$ or $C_{c}$ at the operating point (where $C_{a}$ is 420 ppm). [Color figure can be viewed at wileyonlinelibrary.com]

The complicated shape of the likelihood function for Variable *J* fitting causes frequent fitting failures when using quasi‐Newton or gradient descent optimizers, preventing estimates of $g_{\mathrm{mc}}$ in 6%–24% of curves (Moualeu‐Ngangue et al. 2017). As with C_3_ *A*‐*C*_*i*_ curves, *PhotoGEA* alleviates this issue by using maximum likelihood regression and derivative‐free optimizers when fitting C_3_ *A‐C_i_* + CF curves, and it expresses parameter uncertainties using confidence intervals. Outputs from *fit_c3_variable_j* include estimated values of the FvCB model parameters and τ, along with $A_{n}$, $A_{c}$, $A_{j}$, $A_{p}$, $g_{\mathrm{mc}}$, J and $J_{\mathrm{actual}}$ at each point in the curve, enabling users to evaluate the fit results on a $C_{i}$ or $C_{c}$ basis (Figure 4a,b) and to investigate changes in $g_{\mathrm{mc}}$ and $J_{\mathrm{actual}}$ with changes in CO_2_ concentration (Figure 4c,d). As expected, $J_{\mathrm{actual}}=J$ when assimilation is limited by RuBP regeneration and $J_{\mathrm{actual}}<J$ for other points (Figure 4d). Across the curves analysed here, the fits generally produce similar trends of $g_{\mathrm{mc}}$ and $C_{i}$, with the exception of the lowest $C_{i}$ value, where there were large differences between the curves (Figure 4e and Supporting Information S1: Figures S10–S13). At the second‐lowest $C_{i}$ value, the estimated $g_{\mathrm{mc}}$ values were negative for all curves. A negative $g_{\mathrm{mc}}$ indicates that gas flows from smaller to larger concentrations, at odds with normal diffusion (Equation 5). This is consistent with the understanding of $g_{\mathrm{mc}}$ as an ‘effective’ conductance representing two nonsequential gas flow paths, which can become negative under certain circumstances when $A_{n}<0$ (Tholen et al. 2012). The calculated confidence intervals indicate tight constraints on the value of τ for each curve, although the individual best‐fit τ values vary between curves, where the coefficient of variance is 0.042/0.426 = 9.9% (Table 2).

**Table 2:** Best‐fit values of τ estimated from each of 10 soybean *A‐C_i_* + CF curves using the *fit_c3_variable_j* function from *PhotoGEA*, along with lower and upper limits of the associated confidence intervals.

Curve ID	τ lower limit *(dimensionless)*	τ best‐fit *(dimensionless)*	τ upper limit *(dimensionless)*
2021 ‐ ripe1 ‐ 5	0.412	0.415	0.415
2021 ‐ ripe2 ‐ 1	0.427	0.431	0.431
2021 ‐ ripe2 ‐ 5	0.462	0.465	0.465
2021 ‐ ripe3 ‐ 4	0.370	0.372	0.372
2021 ‐ ripe4 ‐ 4	0.400	0.402	0.402
2022 ‐ ripe15 ‐ 1	0.432	0.434	0.435
2022 ‐ ripe15 ‐ 4	0.499	0.502	0.506
2022 ‐ ripe2 ‐ 1	0.443	0.446	0.446
2022 ‐ ripe2 ‐ 4	0.431	0.433	0.433
2022 ‐ ripe2 ‐ 5	0.359	0.362	0.362

*Note:* The mean and standard deviation of the best‐fit values are 0.426 and 0.042, respectively.

### 3.5 Fitting C_4_ *A‐C_i_* Curves With Both Mechanistic and Empirical Models

Across the literature, both mechanistic and empirical models are used to represent net CO_2_ assimilation in C_4_ plants and to fit C_4_ *A*‐*C*_*i*_ curves. In the mechanistic von Caemmerer model for C_4_ leaves (von Caemmerer 2000; 2021), assimilation can be limited by phosphoenolpyruvate (PEP) carboxylase activity, PEP regeneration, Rubisco activity, or electron transport, and key parameters that can be estimated from a curve fit include the maximum rate of PEP carboxylase activity, the maximum rate of Rubisco activity, and the potential whole‐chain electron transport rate ($V_{p\max}$, $V_{c\max}$ and J). When assimilation is limited by PEP carboxylase, PEP regeneration, Rubisco activity, or light, we denote the corresponding rates by $A_{\mathrm{pc}}$, $A_{\mathrm{pr}}$, $A_{r}$ and $A_{j}$, respectively. In the model, the assimilation rate co‐limited by PEP carboxylase, PEP regeneration, and Rubisco activity is referred to as the enzyme‐limited rate and denoted by $A_{c}$, and the overall rate is given by the minimum of $A_{c}$ and $A_{j}$. See Supporting Information S1: Section S2 for a full description of the model equations.

PEP carboxylase activity generally limits $A_{n}$ at low values of $C_{i}$, and the initial part of a C_4_ *A*‐*C*_*i*_ curve plays the largest role in determining estimates of $V_{p\max}$. The remaining processes limit $A_{n}$ at high values of $C_{i}$. However, they each produce a similar dependence of $A_{n}$ on $C_{i}$, so in practice, it is rarely possible to distinguish between them when fitting a curve. Many studies, such as Markelz et al. (2011), resolve this issue by using an alternative empirical model that represents the response of $A_{n}$ to $C_{i}$ as a non‐rectangular hyperbola (Supporting Information S1: Section S3). Rather than estimating $V_{c\max}$ or J, this approach enables estimates of $V_{\max}$, a parameter that has no mechanistic basis but is related to the maximum net CO_2_ assimilation rate ($A_{\max}$) by $A_{\max}=V_{\max}-R_{L}$. Even when taking the empirical approach to $V_{\max}$, the mechanistic von Caemmerer model is used to estimate $V_{p\max}$ from the low $C_{i}$ response by fitting a subset of points where $C_{i}$ lies below a threshold value, often chosen to be 50–60 μmol mol^−1^ (Supporting Information S1: Section S2.5).

**Figure 5:**
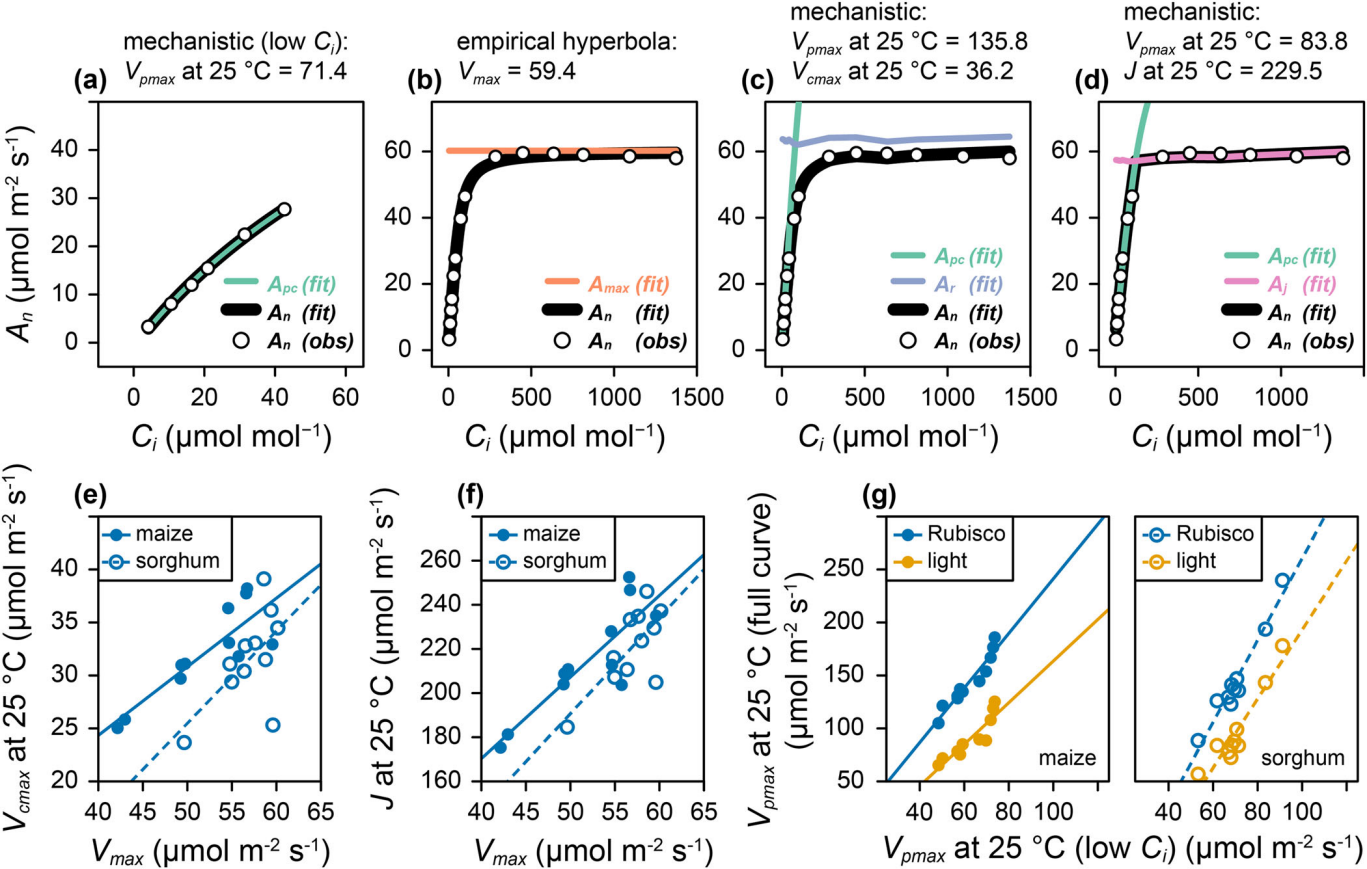
Fitting maize and sorghum A‐C_i_ curves using the *fit_c4_aci* and *fit_c4_aci_hyperbola* functions from *PhotoGEA*. (a–d) Fits of a single sorghum curve (designated ‘sorghum – ripe1 – 2 – 2021’) showing estimated parameter values in units of μmol m^−2^ s^−1^. (a) Fitting the low‐$C_{i}$ portion with the mechanistic model to estimate $V_{p\max}$. (b) Fitting the whole curve with the empirical hyperbola to estimate $V_{\max}$. (c) Fitting the whole curve with the mechanistic model (assuming Rubisco limitations at high $C_{i}$) to estimate $V_{p\max}$ and $V_{c\max}$. (d) Fitting the whole curve with the mechanistic model (assuming light limitations at high $C_{i}$) to estimate $V_{p\max}$ and J. (e, f) $V_{c\max}$ and J, respectively, plotted against $V_{\max}$ as estimated from each curve in the full set. (g) $V_{p\max}$ estimated from whole curve fits assuming either Rubisco or light limitations at high $C_{i}$, plotted against $V_{p\max}$ estimated from the low‐$C_{i}$ portion of each curve. Lines in (e–g) show linear fits (see Supporting Information S1: Table S2 for slopes, intercepts, *R* ^2^ values, and *p*‐values). [Color figure can be viewed at wileyonlinelibrary.com]

The empirical hyperbola is widely used, likely due to its simplicity, but it does not produce estimates of meaningful biochemical parameters. In *PhotoGEA*, it is straightforward to fit the mechanistic von Caemmerer model using the *fit_c4_aci* function, or the empirical hyperbolic model using the *fit_c4_aci_hyperbola* function. This flexibility enables users to compare different approaches to fitting and parameter estimation (Figure 5a–d). Following the semi‐empirical approach, $A_{\mathrm{pc}}$ can be fit to the measured points with $C_{i}$ ≤ 60 μmol mol^−1^ to estimate $V_{p\max}$ (Figure 5a) and a hyperbola can be fit to the entire curve to estimate $V_{\max}$ (Figure 5b). Taking the mechanistic approach, the von Caemmerer model can be fit to the entire curve, assuming either Rubisco limitations to estimate $V_{p\max}$ and $V_{c\max}$, or light limitations to estimate $V_{p\max}$ and J (Figure 5c,d). Fits made assuming either Rubisco or light limitations are similar in quality, illustrating the general difficulty in distinguishing between these limiting factors. Because of this, the estimated $V_{c\max}$ and J values should each be interpreted as lower limits to the true values. Estimates of $V_{p\max}$ depend on whether they are made from the low $C_{i}$ points alone (Figure 5a), from the whole curve assuming Rubisco limitations at high $C_{i}$ (Figure 5c), or from the whole curve assuming light limitations at high $C_{i}$ (Figure 5d).

This procedure can be applied to multiple maize and sorghum *A*‐*C*_*i*_ curves to identify general differences between the fitting methods and species (Figure 5e–g and Supporting Information S1: Figures S14–S18). Since $V_{\max}$, $V_{c\max}$ and J are all determined by the plateau in $A_{n}$ at high $C_{i}$, correlations between them are expected. In fact, the estimated values of $V_{c\max}$ and J are each positively correlated with $V_{\max}$, and the relationship between them is different for maize and sorghum (Figure 5e,f and Supporting Information S1: Table S2). In general, $V_{\max}$ values are slightly larger than $V_{c\max}$ and much smaller than J. Estimates of $V_{p\max}$ from whole‐curve fits are also correlated with estimates of $V_{p\max}$ made from just the low $C_{i}$ points (Figure 5g). Estimates of $V_{p\max}$ made by assuming light limitations is generally in close agreement with the low $C_{i}$ estimates, while estimates of $V_{p\max}$ made by assuming Rubisco limitations are typically larger. This difference is related to a detail of the mechanistic model, where co‐limitations between PEP carboxylase and Rubisco are included, but co‐limitations between PEP carboxylase and electron transport are not. Co‐limitation causes $A_{n}$ to be lower than either $A_{\mathrm{pc}}$ or $A_{r}$ (Figure 5c), requiring $V_{p\max}$ to be larger to achieve the same $A_{n}$ as compared to the light‐limited case where there is a sharp transition from ${A_{n}=A}_{\mathrm{pc}}$ to ${A_{n}=A}_{j}$ (Figure 5d).

## 4 Discussion

Here, we have introduced *PhotoGEA*, an *R* package with tools for fitting CO_2_ response curves, where each tool estimates parameter values using derivative‐free optimizers and calculates non‐Gaussian confidence intervals from likelihood ratios. Unreliable estimates for some parameters, such as J, $V_{c\max}$, $T_{p}$, are identified by confidence intervals whose upper limits extend to infinity, ensuring that only reliable values are returned. These features are not found in any other fitting tools. To illustrate the utility of this approach, the real‐world performance of *PhotoGEA*'s C_3_ *A*‐*C*_*i*_ fitting tool was compared to several other leading tools (*PCE calculator*, *plantecophys*, *photosynthesis* and *msuRACiFit*) using a set of tobacco *A*‐*C*_*i*_ curves. These curves were measured across a range of $Q_{\mathrm{in}}$, and some exhibit TPU limitations and even reverse sensitivity. Due to the presence of TPU limitations, only tools capable of modelling TPU‐limited assimilation were included in the test. In these comparisons, *PCE calculator* can be considered as the ‘benchmark’ method. In contrast to the other tools, where algorithms determine the limiting processes at each point, a plant ecophysiologist ensures that the assignments are biologically reasonable, based on trends in the measured $A_{n}$ and $\varphi_{\mathrm{PSII}}$ values with $C_{i}$.

Across all the curves, *PCE calculator* and *PhotoGEA (* $\alpha_{\mathrm{old}}$ *)* make nearly identical estimates for J, $V_{c\max}$, $T_{p}$ and $R_{L}$ (Figure 2c–f). This includes curves where manual analysis showed that no points were TPU‐limited (and hence $T_{p}$ could not be estimated). Such remarkable agreement shows that *PhotoGEA*'s algorithm generally makes the same limiting process identification as an expert scientist. Some disagreement between *PCE calculator* and *PhotoGEA (* $\alpha_{\mathrm{old}}$ *)* occurs for $Q_{\mathrm{in}}$ values between 800 and 1000 μmol m^−2^ s^−1^, where *PhotoGEA (* $\alpha_{\mathrm{old}}$ *)* indicates that reliable J estimates cannot be made. Yet, even for these curves, the estimates are consistent in the sense that the lower limits from *PhotoGEA (* $\alpha_{\mathrm{old}}$ *)* lie below the *PCE calculator* estimates. It is possible that *PhotoGEA*'s algorithm is overly cautious here; however, in this range, the J values estimated by *PCE calculator* and *photosynthesis* show substantial differences, despite close agreement at all other values of $Q_{\mathrm{in}}$, suggesting that *PhotoGEA (* $\alpha_{\mathrm{old}}$ *)* was correct in identifying these curves as not exhibiting unambiguous RuBP‐regeneration limitations. In fact, an in‐depth analysis of one curve in this range shows that the identification of an RuBP‐regeneration‐limited point in the *PCE calculator* fit was uncertain (Section 3.1). No other tool matched the *PCE calculator* outputs as closely as *PhotoGEA (* $\alpha_{\mathrm{old}}$ *)*, especially regarding estimates of $T_{p}$, where some tools returned estimates for curves where *PCE calculator* did not find any TPU limitations (*plantecophys*, *msuRACiFit* and *PhotoGEA [* $\alpha_{G}$,$\alpha_{S}$ *]*), and one did not return estimates for curves where *PCE calculator* did find TPU limitations (*photosynthesis*). Yet, a key advantage of *PhotoGEA* over *PCE calculator* is that *PhotoGEA* fits are fully automated and do not rely on user judgement when identifying limiting processes.

Other potential issues with some tools were discovered through this comparison. The *plantecophys* and *photosynthesis* tools returned ‘inadmissible fits’ for 30 and 19 of the 36 curves, respectively (Supporting Information S1: Section S4.1). At the two lowest $Q_{\mathrm{in}}$, the modelled assimilation rates returned by the *photosynthesis* tool were often not close to the measured rates, leading to large RMSE values (Figure 2b and Supporting Information S1: Figure S3). However, parameter estimates from these tools were often reasonable for curves where inadmissible fits or high RMSE values were returned, indicating a disconnect between the apparent fit quality and the reliability of its parameter estimates. Thus, in practice, these issues interfere with a user's ability to evaluate whether a fit from one of these tools is reasonable. It is likely they stem from implementation‐specific details that can be addressed in future releases of these tools.

It is also clear that the TPU model used by each tool plays a large role in determining its parameter estimates, even beyond values of $T_{p}$. The two tools using the updated model for TPU (*msuRACiFit* and *PhotoGEA [* $\alpha_{G}$,$\alpha_{S}$ *]*) tend to produce higher estimates of J and $R_{L}$ than the other tools, and they always return estimates of $T_{p}$, even for curves measured with $Q_{\mathrm{in}}$ below 300 μmol m^−2^ s^−1^. Visual inspection of these curves and their corresponding $\phi_{\mathrm{PSII}}$ values indicates no evidence for TPU limitations (Supporting Information S1: Figure S5), as indicated by the *PCE calculator* results. Estimated values of $T_{p}$ in this range when using the updated TPU model are thus most likely the result of overfitting because the updated TPU model has one additional parameter compared to the older one. It may be the case that the updated TPU model requires more ‘intensive’ response curves to ensure reliable results. On the other hand, *plantecophys* and *photosynthesis* tend to estimate higher $T_{p}$ as compared to the other tools, likely because they cannot fit reverse sensitivity to CO_2_ via $\alpha_{\mathrm{old}}$, $\alpha_{G}$, $\alpha_{S}$ or $\alpha_{T}$. Although it is possible to manually set nonzero $\alpha_{\mathrm{old}}$ for each curve using these tools, this process is cumbersome and not practical for large data sets with many curves.

One practical complication that arose when comparing these tools is that each *R* package uses different variable names, has different input requirements, and returns different output types, necessitating a large number of reformatting operations in the code. Interoperability across packages can likely be improved through increased standardization in future releases of these packages, including *PhotoGEA* (Ely et al. 2021; Tholen 2024).

A potential shortcoming of the comparisons above is that the true values of each parameter are not known beforehand. To address this, 600 simulated C_3_ *A*‐*C*_*i*_ curves were generated and fit with each tool (Supporting Information S1: Section S10). For these simulated curves, the true parameter values are known beforehand, as well as the number of points in each curve that are limited by Rubisco activity, RuBP regeneration, or TPU. In general, results from simulated curves are similar to those discussed above. In brief, *PhotoGEA* is shown to produce the closest parameter estimates, and is best able to identify when one or more potential limiting process is not evident in a curve. Estimates of $R_{L}$ made using the updated TPU model (*PhotoGEA [* $\alpha_{G}$,$\alpha_{S}$ *]* and *msuRACiFit*) are less accurate than other estimates, and in general, estimates made using this model are prone to false positives (where a parameter estimate is returned even though no points in the curve are limited by the corresponding process), illustrating the difficulties associated with using it for curve fits. The *photosynthesis* tool is prone to false negatives when estimating $T_{p}$, while *plantecophys* is prone to false positives when estimating $T_{p}$. This discrepancy highlights the role of implementation‐specific code details in determining a tool's results, since *plantecophys* and *photosynthesis* both use an exhaustive approach with the same TPU model. For a detailed discussion of these fits, see Supporting Information S1: Section S10.

The measured tobacco *A*‐*C*_*i*_ fits also revealed clear trends in J, $V_{c\max}$ and $T_{p}$ with $Q_{\mathrm{in}}$, consistently observed across results from all tools (Figure 2c–e). Although the dependence of J on $Q_{\mathrm{in}}$ is well‐known and commonly described using a non‐rectangular hyperbola (von Caemmerer 2000), the other responses are less understood, and no established equations are available for describing them. For $V_{c\max}$, the Rubisco concentration ($n_{\mathrm{Rubisco}}$) and carboxylation rate constant ($k_{\mathrm{cat}}$) are not expected to change with $Q_{\mathrm{in}}$. Instead, this trend may be due to changes in the number of active Rubisco sites ($E_{t}$) or in mesophyll conductance; both influence apparent $V_{\mathrm{cmax}}$ and are known to depend on environmental conditions such as $Q_{\mathrm{in}}$ (Sage et al. 2002; Théroux‐Rancourt and Gilbert 2017). The trend in $T_{p}$ may indicate coordination of TPU with Rubisco activity and RuBP regeneration limitations (Sharkey 2019). Estimated values of $\alpha_{\mathrm{old}}$ also increase with $Q_{\mathrm{in}}$, and even reach $\alpha_{\mathrm{old}}>1$ for some curves (Figure 3d and Supporting Information S1: Figure S7), indicating a stronger degree of reverse sensitivity at high light. When treated as a mechanistic factor, $\alpha_{\mathrm{old}}$ is expected to be between zero and one. Yet, because of measurement noise, it may be the case that the best‐fit value lies outside this range. Constraining $\alpha_{\mathrm{old}}$ to this range can be done in *PhotoGEA*, but doing so could introduce a bias to the fits. Values above one have been noted before, and it has been suggested that because of this, $\alpha_{\mathrm{old}}$ should ‘be considered an arbitrary parameter useful for the comparison of the degree of reverse sensitivity, but without a mechanistic basis’ (Sharkey 2016). Similar constraints apply to the values of $\alpha_{G}$, $\alpha_{S}$ and $\alpha_{T}$ (Busch 2020), and these constraints can also be enforced in *PhotoGEA*, although they were not enforced here to avoid bias (Supporting Information S1: Figure S7).

Compared to fitting C_3_ *A*‐*C*_*i*_ curves, fewer tools are currently available for applying the Variable *J* fitting method to C_3_ *A*‐*C*_*i*_ + CF curves or for fitting C_4_ *A*‐*C*_*i*_ curves. Thus, the *fit_c3_variable_j* and *fit_c4_aci* functions from *PhotoGEA* fulfil an important need. Sections 3.4 and 3.5 illustrate some of the insights that can be gained through these tools. For example, Variable *J* fits are shown to place narrow constraints on values of τ, while τ can vary by more than 10% between tobacco leaves (Table 2). Variations in absorptance between tobacco leaves of similar age and health, grown in the same environment, are typically smaller than 10%, indicating that other sources of variation in τ must be present. It should be noted that Equation (3) is a simplification that does not include alternative electron acceptors or nonlinear electron transport (Flexas et al. 2007; Gilbert et al. 2012; van der van der Putten et al. 2018); thus the parameter β as used in Equation (3) likely represents the effective influence of multiple processes beyond the partitioning of light energy to Photosystem II, and its value is difficult to estimate or measure. Such processes likely contribute to variation in effective values of β, and hence to the observed variation in τ. These results emphasize the necessity of a reliable Variable *J* fitting tool that does not require a β estimate, and highlight the inherent uncertainty in assuming a β value of 0.5.

Another insight is that empirical estimates of $V_{\max}$ from C_4_ *A*‐*C*_*i*_ curves can likely be related to the mechanistic parameters $V_{c\max}$ and J through crop‐specific correlations (Figure 5e–f), and that values of $V_{p\max}$ and $V_{c\max}$ estimated from curves can be compared to in vitro biochemical assay measurements to determine which fitting assumptions regarding limitations at high $C_{i}$ are most appropriate. For maize, in vitro $V_{p\max}$ at 25°C has been reported to be 193–243 μmol m^−2^ s^−1^ (Sonawane et al. 2018; Salesse‐Smith et al. 2018). Curve fits made at low $C_{i}$ or by assuming light limitations result in substantially lower estimates ($V_{p\max}$ < 125 μmol m^−2^ s^−1^), while curve fits made assuming Rubisco limitations reach up to $V_{p\max}$ = 185 μmol m^−2^ s^−1^ (Figure 5g), suggesting that Rubisco limitations may be a more reasonable assumption and that the J estimates are lower bounds on the true value, although this would require further investigation to verify.

Beyond this, because these fitting functions are implemented in *R*, it is straightforward to apply them to sets of curves using different settings or parameter values, enabling users to investigate the sensitivity of fitted parameter values to key inputs like $\Gamma^{*}$ (for Variable *J*) and bundle sheath conductance (for C_4_). This can help to generate a more nuanced understanding of when outputs such as $g_{\mathrm{mc}}$ are most reliable. For example, it has been suggested that whenever the derivative $dC_{c}/dA_{n}$ is below 10 or above 50 bar m^2^ s mol^−2^, mesophyll conductances estimated with Equation (4) may be unreliable due to uncertainties in the value of $\Gamma^{*}$ (Harley et al. 1992). This simple rule could be replaced by a sensitivity analysis showing the range of $g_{\mathrm{mc}}$ values estimated from fits with different assumed values of $\Gamma^{*}$.

Besides offering a unique and reliable approach to estimating parameter values from CO_2_ response curves, *PhotoGEA* enables automation for quickly analysing large data sets, uses the latest models of photosynthetic biochemistry and provides detailed example scripts to increase its accessibility to researchers who may not be *R* experts. In the particular case of C_3_ *A*‐*C*_*i*_ curves, *PhotoGEA* produced nearly identical results as the *PCE calculator* tool, but without requiring time‐consuming manual operations or relying on user judgement. Finally, we note that *PhotoGEA* has additional features not discussed here, such as functions for estimating Ball‐Berry model parameters (Ball et al. 1987), calculating limitations to C_3_ photosynthesis (Warren et al. 2003; Grassi and Magnani 2005), fitting response curves with the Laisk method (Laisk 1977; Walker and Ort 2015), and analysing isotope discrimination measurements from tunable diode laser systems (Ubierna et al. 2018; Busch et al. 2020). Thus, *PhotoGEA* will be of great use to many plant scientists, and in fact, it has already played a key role in several studies (Salesse‐Smith, Lochocki, et al. 2024; Salesse‐Smith, Adar, et al. 2025; Pelech et al. 2025).

## Conflicts of Interest

The authors declare no conflicts of interest.

## Supporting information

The Supplemental Information includes Supplemental Sections **S1** (*Mechanistic Model of C*_*3*_ *Steady State Carbon Assimilation*), **S2** (*Mechanistic Model of C*_*4*_ *Steady State Carbon Assimilation*), **S3** (*Empirical Hyperbolic Model of C*_*4*_ *Steady State Carbon Assimilation*), **S4** (*C*_*3*_ *A‐Ci Fitting Results*), **S5** (*C*_*3*_ *A‐Ci* + *CF Variable J Fitting Results*), **S6** (*C*_*4*_ *A‐Ci Fitting Results*), **S7** (*Temperature Response in PhotoGEA*), **S8** (*Likelihood and Confidence Intervals in PhotoGEA*), **S9** (*Detailed Computational Methods*) and **S10** (*Fitting Simulated C*_*3*_ *A‐C*_*i*_ *Curves With Each R Package*).

## Data Availability

All data and *R* scripts for reproducing all analysis contained in this study are available online at https://github.com/ripeproject/PhotoGEA-paper. The *PhotoGEA* package code, installation instructions, and other documentation are available online at https://github.com/eloch216/PhotoGEA.
