# Supplementary material for: PhotoGEA: An R Package for Closer Fitting of Photosynthetic Gas Exchange Data With Non‐Gaussian Confidence Interval Estimation
Source: Plant Cell Environ. 2025 Mar 30;48(7):5104–19. doi: 10.1111/pce.15501 (PMC12131965; doi:10.1111/pce.15501)
Supplement: Supplementary file 1 — The Supplemental Information includes Supplemental Sections S1 (Mechanistic Model of C 3 Steady State Carbon Assimilation), S2 (Mechanistic Model of C 4 Steady State Carbon Assimilation), S3 (Empirical Hyperbolic Model of C 4 Steady State Carbon Assimilation), S4 (C 3 A‐Ci Fitting Results), S5 (C 3 A‐Ci + CF Variable J Fitting Results), S6 (C 4 A‐Ci Fitting Results), S7 (Temperature Response in PhotoGEA), S8 (Likelihood and Confidence Intervals in PhotoGEA), S9 (Detailed Computational Methods) and S10 (Fitting Simulated C 3 A‐C i Curves With Each R Package). [file PCE-48-5104-s001.docx]

**PhotoGEA: An R Package for Closer Fitting of Photosynthetic Gas Exchange Data With Non-Gaussian Confidence Interval Estimation**

**Edward B. Lochocki^1^_,_ Coralie E. Salesse-Smith^1^, and Justin M. McGrath^1,2,3*^**

**^1^**Carl R. Woese Institute for Genomic Biology, University of Illinois, Urbana–Champaign, Urbana, IL, USA

**^2^**Plant Biology Department, University of Illinois, Urbana–Champaign, Urbana, IL, USA

**^3^**USDA ARS Global Change and Photosynthesis Research Unit, Urbana, IL, USA

*****Corresponding author’s email address: justin.mcgrath@usda.gov

**Supplemental Information**

Contents

[S1. Mechanistic Model of C_3_ Steady State Leaf Carbon Assimilation 3](#_Toc191305423)

[S1.1 The Original Version 3](#_Toc191305424)

[S1.2 The Updated Version 4](#_Toc191305425)

[S1.3 Process-Limited Assimilation Rates 5](#_Toc191305426)

[S2. Mechanistic Model of C_4_ Steady State Leaf Carbon Assimilation 6](#_Toc191305427)

[S2.1 Overview 6](#_Toc191305428)

[S2.1 Enzyme Limited Assimilation 6](#_Toc191305429)

[S2.3 Light Limited Assimilation 7](#_Toc191305430)

[S2.4 Understanding the Model in a Simplified Scenario 8](#_Toc191305431)

[S2.5 Estimating *V_pmax_* from the Initial Slope of an *A*-*C_i_* Curve 9](#_Toc191305432)

[S2.6 Other Considerations for Curve Fitting 9](#_Toc191305433)

[S3. Empirical Hyperbolic Model of C_4_ Steady State Leaf Carbon Assimilation 10](#_Toc191305434)

[S4. C_3_ A-C_i_ Fitting Results 11](#_Toc191305435)

[S4.1 Limiting Factors For One Curve 11](#_Toc191305436)

[S4.2 Fits from Each Tool 12](#_Toc191305437)

[S4.3 Each Curve 17](#_Toc191305438)

[S4.4 Comparison of Akiake Information Criterion (AIC) values 18](#_Toc191305439)

[S4.5 Comparison of Estimated α Values 19](#_Toc191305440)

[S4.6 Comparison of Estimated *J* and *T_p_* Values from Each Curve 20](#_Toc191305441)

[S5. C_3_ *A*-*C_i_* + CF Variable *J* Fitting Results 22](#_Toc191305442)

[S6. C_4_ *A*-*C_i_* Fitting Results 27](#_Toc191305443)

[S6.1 Correlations 27](#_Toc191305444)

[S6.2 Fits at Low C_i_ 28](#_Toc191305445)

[S6.3 Fits with the Empirical Hyperbola 29](#_Toc191305446)

[S6.4 Fits Assuming Rubisco Limitations 30](#_Toc191305447)

[S6.5 Fits Assuming Light Limitations 31](#_Toc191305448)

[S6.6 Each Curve 32](#_Toc191305449)

[S7. Temperature Response in *PhotoGEA* 33](#_Toc191305450)

[S8. Likelihood and Confidence Intervals in *PhotoGEA* 35](#_Toc191305451)

[S9. Detailed Fitting Settings for Each Tool 37](#_Toc191305452)

[S9.1 C_3_ *A*-*C_i_* curves 37](#_Toc191305453)

[S9.2 C_3_ *A*-*C_i_* + CF curves 39](#_Toc191305454)

[S9.3 C_4_ *A*-*C_i_* curves 40](#_Toc191305455)

[S10. Fitting Simulated C_3_ *A*-*C_i_* Curves With Each *R* Package 41](#_Toc191305456)

[S10.1 Curves Without Reverse Sensitivity 41](#_Toc191305457)

[S10.2 Curves With Reverse Sensitivity (α_old_) 46](#_Toc191305458)

[S10.3 Curves with Reverse Sensitivity (α_G_ and α_S_) 48](#_Toc191305459)

[Works Cited 50](#_Toc191305460)

# S1. Mechanistic Model of C_3_ Steady State Leaf Carbon Assimilation

## S1.1 The Original Version

The mechanistic model of C_3_ steady state leaf carbon assimilation was first described in Farquhar, von Caemmerer, and Berry (1980) and Farquhar and von Caemmerer (1982) and is commonly referred to as the Farquhar-von-Caemmerer-Berry (FvCB) model in recognition of its original authors. The first version of the model only considered two potential processes that may limit photosynthesis: Rubisco activity and RuBP regeneration. Later, it was shown that TPU also plays a role in determining leaf photosynthesis rates in some situations, and the model was updated to include this third process (Sharkey 1985; Kirschbaum and Farquhar 1984; von Caemmerer 2000). Across the thousands of publications that use the FvCB model, the equations are not always written the same way, and some of the variations in the equations introduce errors into the model. To avoid these errors, it is essential to choose between limiting states using the smallest potential carboxylation rate and to include a model-based CO_2_ concentration threshold below which TPU limitations cannot occur (Lochocki and McGrath 2025).

*PhotoGEA* implements the equations as written in Lochocki and McGrath (2024) while also adopting the $\alpha_{old}$ notation from Busch, Sage, and Farquhar (2018). In this version, the model consists of five equations:

$W_{c}=\frac{C_{c}\cdot V_{cmax}}{C_{c}+K_{c}\cdot\left( 1+\frac{O}{K_{o}} \right)}$ (**A1**)

$W_{j}=\frac{C_{c}\cdot J}{4C_{c}+8\Gamma^{*}}$ (**A2**)

$W_{p}=\left\{ \begin{matrix} \infty, & C_{c}\leq\Gamma^{*}\cdot\left( 1+3\alpha_{old} \right) \\ \frac{3\cdot C_{c}\cdot T_{p}}{C_{c}-\Gamma^{*}\cdot\left( 1+3\alpha_{old} \right)}, & C_{c}>\Gamma^{*}\cdot\left( 1+3\alpha_{old} \right) \end{matrix} \right.$ (**A3**)

$V_{c}=\text{min}\left\{ W_{c},W_{j},W_{p} \right\}$ (**A4**)

$A_{n}=V_{c}\cdot\left( 1-\frac{\Gamma^{*}}{C_{c}} \right)-R_{L}$ (**A5**)

where $W_{c}$, $W_{j}$, and $W_{p}$ are the potential RuBP carboxylation rates limited by Rubisco activity, RuBP regeneration, and TPU; $C_{c}$ and $O$ are the partial pressures of CO_2_ and O_2_ in the chloroplast; $V_{cmax}$ is the maximum rate of Rubisco carboxylation; $K_{c}$ and $K_{o}$ are the Michaelis-Menten constants for Rubisco carboxylation and oxygenation; $J$ is the potential rate of whole-chain electron transport; $\Gamma^{*}$ is the CO_2_ compensation point in the absence of non-photorespiratory CO_2_ release; $T_{p}$ is the maximum rate of triose phosphate utilization; $\alpha_{old}$ is the fraction of glycolate carbon not returned to the chloroplast after accounting for carbon released as CO_2_; $V_{c}$ is the actual RuBP carboxylation rate; $A_{n}$ is the net CO_2_ assimilation rate; and $R_{L}$ is the rate of non-photorespiratory CO_2_ release in the light. Note that when $C_{c}\leq\Gamma^{*}\cdot\left( 1+3\cdot\alpha_{old} \right)$, $W_{p}$ is infinitely large (Equation **A3**), so that$\text{min}\left\{ W_{c},W_{j},W_{p} \right\}$ cannot be $W_{p}$. In this case, $V_{c}=\text{min}\left\{ W_{c},W_{j} \right\}$ (Equation **A4**); in other words, TPU limitations cannot occur.

In the context of fitting C_3_ *A*-*C_i_* curves, values of $C_{c}$ may not be available. In this case it is common to assume that $C_{c}=C_{i}$. If mesophyll conductance values are known or can be estimated, then $C_{c}$ can be calculated using a one-dimensional diffusion equation (Equation **5** in the main text). Setting mesophyll conductance ($g_{mc}$) to infinity in this equation produces $C_{c}=C_{i}$. In *PhotoGEA*, the FvCB model is implemented via the *calculate_c3_assimilation* function.

## S1.2 The Updated Version

In Equation **A3** above, the parameter $\alpha_{old}$ accounts for carbon that remains in the cytosol as either glycine or serine as a byproduct of the photorespiratory cycle. In an updated version of the FvCB model, separate terms are included for glycolate carbon leaving the photorespiratory pathway in the form of glycine ($\alpha_{G}$), serine ($\alpha_{S}$), or 5,10-methylene-tetrahydrofolate (CH_2_-THF) ($\alpha_{T}$) (Busch, Sage, and Farquhar 2018; Busch 2020). Furthermore, the number of CO_2_ molecules released following each RuBP oxygenation is adjusted from $0.5$ to $0.5\cdot\left( 1-\alpha_{G} \right)+\alpha_{T}$ to account for carbon leaving the photorespiratory pathway as glycine and CH_2_-THF, and the electron transport requirements for RuBP regeneration are also adjusted. In this updated version of the model, Equations **A1-5** above become

$W_{c}=\frac{C_{c}\cdot V_{cmax}}{C_{c}+K_{c}\cdot\left( 1+\frac{O}{K_{o}} \right)}$ (**A6**)

$W_{j}=\frac{C_{c}\cdot J}{4\cdot C_{c}+\left( 8+16\alpha_{G}-8\alpha_{T}+8\alpha_{S} \right)\cdot\Gamma_{\alpha_{GT}}^{*}}$ (**A7**)

$W_{p}=\left\{ \begin{matrix} \infty, & C_{c}\leq\Gamma_{\alpha_{GT}}^{*}\cdot\left( 1+3\alpha_{G}+6\alpha_{T}+4\alpha_{S} \right) \\ \frac{3\cdot C_{c}\cdot T_{p}}{C_{c}-\Gamma_{\alpha_{GT}}^{*}\cdot\left( 1+3\alpha_{G}+6\alpha_{T}+4\alpha_{S} \right)}, & C_{c}>\Gamma_{\alpha_{GT}}^{*}\cdot\left( 1+3\alpha_{G}+6\alpha_{T}+4\alpha_{S} \right) \end{matrix} \right.$ (**A8**)

$V_{c}=\text{min}\left\{ W_{c},W_{j},W_{p} \right\}$ (**A9**)

$A_{n}=V_{c}\cdot\left( 1-\frac{\Gamma_{\alpha_{GT}}^{*}}{C_{c}} \right)-R_{L}$ (**A10**)

where $\Gamma_{\alpha_{GT}}^{*}=\Gamma^{*}\cdot\left( 1-\alpha_{G}+2\alpha_{T} \right)$ is the adjusted value of $\Gamma^{*}$ accounting for glycolate carbon remaining in the cytosol as glycine and CH_2_-THF. Note that Equations **A1** and **A6** are identical, that Equations **A4** and **A9** are identical, and that the lower threshold for TPU limitations has become $\Gamma_{\alpha_{GT}}^{*}\cdot\left( 1+3\alpha_{G}+6\alpha_{T}+4\alpha_{S} \right)$. In terms of curve fitting, a key difference between the two versions of the model is that Equations **A6-10** have two more free parameters as compared to Equations **A1-5** because $\alpha_{old}$ has been replaced by $\alpha_{G}$, $\alpha_{S}$, and $\alpha_{T}$. In *PhotoGEA*, this updated version of the FvCB model is implemented via the *calculate_c3_assimilation* function, which includes Equations **A1-10** and can calculate assimilation rates using either version.

## S1.3 Process-Limited Assimilation Rates

Equations **A1-10** above are written in terms of the process-limited carboxylation rates $W_{c}$, $W_{j}$, and $W_{p}$, but it is also possible to define and calculate the corresponding net CO_2_ assimilation rates, which are denoted by $A_{c}$, $A_{j}$, and $A_{p}$, respectively. For example, when carboxylation is limited by Rubisco activity in the original version of the model, $V_{c}=W_{c}$ from Equation **A4** and therefore the Rubisco-limited assimilation rate is given by

$A_{c}=W_{c}\cdot\left( 1-\frac{\Gamma^{*}}{C_{c}} \right)-R_{L}$ (**A11**)

according to Equation **A5**. Likewise, the RuBP-regeneration-limited and TPU-limited assimilation rates are given by

$A_{j}=W_{j}\cdot\left( 1-\frac{\Gamma^{*}}{C_{c}} \right)-R_{L}$ (**A12**)

and

$A_{p}=W_{p}\cdot\left( 1-\frac{\Gamma^{*}}{C_{c}} \right)-R_{L}$. (**A13**)

In the updated version of the model, Equations **A11-A13** become

$A_{c}=W_{c}\cdot\left( 1-\frac{\Gamma_{\alpha_{G}}^{*}}{C_{c}} \right)-R_{L}$ (**A14**)

$A_{j}=W_{j}\cdot\left( 1-\frac{\Gamma_{\alpha_{G}}^{*}}{C_{c}} \right)-R_{L}$ (**A15**)

and

$A_{p}=V_{p}\cdot\left( 1-\frac{\Gamma_{\alpha_{G}}^{*}}{C_{c}} \right)-R_{L}$. (**A16**)

It is essential to note that although Equations **A4-5** and **A9-10** are sometimes replaced by

$A_{n}=\min\left\{ A_{c},A_{j},A_{p} \right\}$, (**A17**)

this equation is not true in either version of the model (Lochocki and McGrath 2025). Although $A_{n}$ is given by the smallest potential assimilation rate when $C_{c}\geq\Gamma^{*}$ in the original version (or when $C_{c}\leq\Gamma_{\alpha_{G}}^{*}$ in the updated version), this relationship does not hold for smaller values of $C_{c}$.

# S2. Mechanistic Model of C_4_ Steady State Leaf Carbon Assimilation

## S2.1 Overview

The mechanistic model for C_4_ steady state leaf carbon assimilation used in *PhotoGEA* is described in von Caemmerer (2000) and von Caemmerer (2021) and implemented as the *calculate_c4_assimilation* function. Here, we reproduce the model’s equations (with small changes to notation for clarity) and introduce some additional terminology. In general, the model calculates the net CO_2_ assimilation rate ($A_{n}$) as the smaller of the enzyme limited rate ($A_{c}$) and the light limited rate ($A_{j}$):

$A_{n}=\min\left\{ A_{c},A_{j} \right\}$. (**B1**)

## S2.1 Enzyme Limited Assimilation

The enzyme limited rate is given by the smaller root of the quadratic equation

$a_{c}{A_{c}}^{2}+b_{c}A_{c}+c_{c}=0$, (**B2**)

where the coefficients $a_{c}$, $b_{c}$, and $c_{c}$ are given by

$a_{c}=1-\frac{\alpha_{PSII}}{a_{o}}\frac{K_{c}}{K_{o}}$, (**B3**)

$b_{c}=-\left[ A_{p}+A_{r}+g_{bs}K_{c}\left( 1+\frac{O_{m}}{K_{o}} \right)+\frac{\alpha_{PSII}}{a_{o}}\left( \gamma_{*}V_{cmax}+R_{L}\frac{K_{c}}{K_{o}} \right) \right]$, (**B4**)

and

$c_{c}=A_{p}A_{r}-g_{bs}\left[ \gamma_{*}V_{cmax}O_{m}+R_{L}K_{c}\left( 1+\frac{O_{m}}{K_{o}} \right) \right]$. (**B5**)

Here, $\alpha_{PSII}$ is the fraction of photosystem II activity occurring in the bundle sheath, $a_{o}$ is the ratio of solubility and diffusivity of O_2_ to CO_2_, $K_{c}$ and $K_{o}$ are the Michaelis-Menten constants of Rubisco for CO_2_ and O_2_, $A_{p}$ and $A_{r}$ are PEP and Rubisco limited net CO_2_ assimilation rates, $g_{bs}$ is the bundle sheath conductance to CO_2_ diffusion, $O_{m}$ is the partial pressure of O_2_ in the mesophyll, $\gamma_{*}$ is half the reciprocal of Rubisco specificity, $V_{cmax}$ is maximum rate of Rubisco activity, and $R_{L}$ is the rate of non-photorespiratory CO_2_ release in the light. As compared to the original presentation of this model, we have introduced two new terms $A_{p}$ and $A_{r}$ in order to simplify Equations **B3-B5** above. These are defined as

$A_{p}=V_{p}-R_{Lm}+g_{bs}C_{m}$ (**B6**)

and

$A_{r}=V_{cmax}-R_{L}$, (**B7**)

where $V_{p}$ is the rate of PEP carboxylase activity, $R_{Lm}$ is the rate of non-photorespiratory CO_2_ release in the light occurring in the mesophyll and $C_{m}$ is the partial pressure of CO_2_ in the mesophyll. PEP carboxylase activity is calculated as the smaller of the CO_2_ limited rate ($V_{pc}$) and the PEP regeneration limited rate ($V_{pr}$):

$V_{p}=\min\left\{ V_{pc},V_{pr} \right\}$. (**B8**)

The terms $V_{pc}$ and $V_{pr}$ are not used in the original description but are introduced here for clarity. Because there are two processes that can potentially limit PEP activity, there are also two potential PEP carboxylase limited net assimilation rates: the CO_2_ and PEP carboxylase limited rate ($A_{pc}$) and the PEP regeneration limited rate ($A_{pr}$). These are given by

$A_{pc}=V_{pc}-R_{Lm}+g_{bs}C_{m}$ (**B9**)

and

$A_{pr}=V_{pr}-R_{Lm}+g_{bs}C_{m}$. (**B10**)

The CO_2_ limited PEP carboxylation rate is given by

$V_{pr}=\frac{C_{m}V_{pmax}}{C_{m}+K_{p}}$, (**B11**)

where $V_{pmax}$ is the maximum rate of PEP carboxylase activity and $K_{p}$ is the Michaelis-Menten constant of PEP carboxylase for CO_2_.

## S2.3 Light Limited Assimilation

Like the enzyme limited rate, the light limited rate is given by the smaller root of the quadratic equation

$a_{j}{A_{j}}^{2}+b_{j}A_{j}+c_{j}=0$, (**B12**)

where the coefficients $a_{j}$, $b_{j}$, and $c_{j}$ are given by

$a_{j}=1-\frac{7\gamma_{*}}{3}\frac{\alpha_{PSII}}{a_{o}}$, (**B13**)

$b_{j}=-\left[ A_{jm}+A_{jbs}+g_{bs}O_{m}\frac{7\gamma_{*}}{3}+\gamma_{*}\frac{\alpha_{PSII}}{a_{o}}\left( \frac{z}{3}\left[ 1-x \right]\cdot J+\frac{7}{3}R_{L} \right) \right]$, (**B14**)

and

$c_{j}=A_{jm}A_{jbs}-g_{bs}\gamma_{*}O_{m}\left( \frac{z}{3}\left[ 1-x \right]\cdot J+\frac{7}{3}R_{L} \right)$. (**B15**)

Here, $z$ is the ratio of the rate of ATP production to linear electron transport, $x$ is the fraction of electron transport occurring in the mesophyll (as opposed to the bundle sheath), $J$ is the linear electron transport rate, and $A_{jm}$ and $A_{jbs}$ are the net assimilation rates limited by electron transport in the mesophyll and bundle sheath, respectively. The terms $A_{jm}$ and $A_{jbs}$ are introduced here to simplify Equations **B13-B15**. They are given by

$A_{jm}=\frac{z}{2}xJ-R_{Lm}+g_{bs}C_{m}$ (**B16**)

and

$A_{jbs}=\frac{z}{3}\left[ 1-x \right]\cdot J-R_{L}$. (**B17**)

The potential whole-chain electron transport rate $J$ can be calculated as the smaller root of another quadratic equation:

$\theta J^{2}-\left( I_{2}+J_{max} \right)J+I_{2}\cdot J_{max}=0$, (**B18**)

where $\theta$ is an empirical curvature factor, $J_{max}$ is the maximum whole-chain electron transport rate, and $I_{2}$ is the useful light partitioned to photosystem II. $I_{2}$ is related to the incident irradiance $I$ according to

$I_{2}=I\cdot abs\cdot\rho\left( 1-f \right)$, (**B19**)

where $abs$ is the leaf absorptance, $\rho$ is the fraction of absorbed light energy partitioned to photosystem II, and $f$ corrects for the spectral quality of light.

## S2.4 Understanding the Model in a Simplified Scenario

Equations **B1-B19** above are complicated and their behavior is complex. The model is easier to understand in a simplified scenario where $g_{bs}$ and $\alpha_{PSII}$ are both zero, and the insights gained from the simplified version can then be applied to the full model. When $g_{bs}=0$, Equations **B2-B5** show that the enzyme limited rate $A_{c}$ is given by the smaller root of

$\left( 1-\frac{\alpha_{PSII}}{a_{o}}\frac{K_{c}}{K_{o}} \right){A_{c}}^{2}-\left( A_{p}+A_{r} \right)A_{c}+A_{p}A_{r}=0$. (**B20**)

This can be recognized as a “smooth minimum” function acting on $A_{p}$ and $A_{r}$ with a curvature given by $1-\frac{\alpha_{PSII}}{a_{o}}\frac{K_{c}}{K_{o}}$. When $\alpha_{PSII}=0$, the curvature is equal to one, and choosing the smaller root of Equation **B20** is equivalent to a simple minimum:

$A_{c}=\min\left\{ A_{p},A_{r} \right\}$. (**B21**)

Likewise, when $g_{bs}=0$, Equations **B12-B15** show that the light limited rate $A_{j}$ is given by the smaller root of

$\left( 1-\frac{7\gamma_{*}}{3}\frac{\alpha_{PSII}}{a_{o}} \right){A_{j}}^{2}-\left( A_{jm}+A_{jbs} \right)A_{j}+A_{jm}A_{jbs}=0$. (**B22**)

This is another “smooth minimum” function acting on $A_{jm}$ and $A_{jbs}$. When $\alpha_{PSII}=0$, the curvature is equal to one, and

$A_{j}=\min\left\{ A_{jm},A_{jbs} \right\}$. (**B23**)

Combining Equations **B21** and **B23** with Equations **B1** and **B8**, the overall assimilation rate is found to be

$A_{n}=\min\left\{ A_{pc},A_{pr},A_{r},A_{jm},A_{jbs} \right\}$. (**B24**)

Thus, the overall rate is the smallest of the five potential rates when $g_{bs}$ and $\alpha_{PSII}$ are both zero. This can be a useful heuristic for understanding C_4_ photosynthesis. Yet, in the full model, the equations deviate from simple minima, and Equations **B21**, **B23**, and **B24** do not hold. For example, $A_{c}$ is actually smaller than the minimum of $A_{p}$ and $A_{r}$ when $\alpha_{PSII}$ is zero but $g_{bs}$ is nonzero, which can be seen in Figure **5c** of the main text. Another feature of the full model is that $A_{r}$ influences $A_{c}$ even when $A_{pc}$ is smaller than $A_{r}$, which means that the initial slope of a C_4_ *A*-*C_i_* curve is sensitive to both $V_{cmax}$ and $V_{pmax}$.

## S2.5 Estimating *V_pmax_* from the Initial Slope of an *A*-*C_i_* Curve

In the literature, it is common to estimate $V_{pmax}$ by fitting the following equation to the low $C_{i}$ part of an *A*-*C_i_* curve:

$A_{n}=\frac{C_{i}V_{pmax}}{C_{i}+K_{p}}-R_{L}$. (**B25**)

In light of the discussion above, this is tantamount to an assumption that $g_{bs}$ and $\alpha_{PSII}$ are both zero, and that mesophyll conductance is infinite (so that $C_{m}=C_{i}$). Under these assumptions, $A_{n}=A_{pc}=\frac{C_{i}V_{pmax}}{C_{i}+K_{p}}-R_{L}$ at low $C_{i}$, in agreement with Equation **B25**. In contrast, when using the full model to estimate $V_{pmax}$, Equations **B2-B5** are used along with a one-dimensional diffusion equation:

$C_{m}=C_{i}-\frac{A_{n}}{g_{m}}$, (**B26**)

where $g_{m}$ is the mesophyll conductance. Because $A_{c}$ is generally smaller than $A_{pc}$ in the full model, the latter approach tends to predict higher values of $V_{pmax}$ than the simplified approach using Equation **B25**, which can be seen in Figure **5g** of the main text.

## S2.6 Other Considerations for Curve Fitting

The mechanistic model for C_4_ steady state leaf carbon assimilation includes separate rates of non-photorespiratory CO_2_ release in the mesophyll ($R_{Lm}$) and for the leaf as a whole ($R_{L}$). In *PhotoGEA*, these are related according to $R_{Lm}=f_{R_{Lm}}\cdot R_{L}$, where $0\leq f_{R_{Lm}}\leq1$ is a fractional multiplier, and only $R_{L}$ is varied while fitting. This is a straightforward extension of the original description of the model, where $R_{Lm}$ is taken to be half of $R_{L}$.

# S3. Empirical Hyperbolic Model of C_4_ Steady State Leaf Carbon Assimilation

A potential issue with the mechanistic model described in Section **S2** is that the predicted response of $A_{n}$ to $C_{i}$ is very similar when assimilation is limited by Rubisco activity, PEP regeneration, or light. Thus, it is difficult in practice to determine which process is limiting, and hence which parameter values can reasonably be estimated. In response to this uncertainty, an empirical model is used in place of the mechanistic model. In this model, $A_{n}$ is given by

$A_{n}=A_{g}-R_{L}$, (**C1**)

where $A_{g}$ is the gross assimilation rate, calculated as the smaller root of a quadratic equation

$\theta_{H}{A_{g}}^{2}-\left( V_{initial}+V_{max} \right)A_{g}+V_{initial}\cdot V_{max}=0$. (**C2**)

Here, $\theta_{H}$ is a curvature parameter, $V_{max}$ is the maximum gross assimilation rate, and $V_{initial}$ represents the initial response of assimilation to increases in CO_2_ availability:

$V_{initial}=m\cdot C_{i}$, (**C3**)

where $m$ is a slope. Together, Equations **C1-C3** produce a shape commonly referred to as a “four parameter hyperbola” or a “non-rectangular hyperbola,” which can been found to fit most experimentally measured C_4_ A-C_i_ curves well. Rather than enabling estimates of $V_{cmax}$, $V_{pr}$, or $J_{max}$ from the high $C_{i}$ part of a curve (as in the mechanistic model), this model enables estimates of $V_{max}$ (or the maximum net assimilation rate $A_{max}=V_{max}-R_{L}$) without relating it to a specific biochemical process. It should be noted that although the slope $m$ is estimated from the response of $A_{n}$ to $C_{i}$ at low $C_{i}$, it is not equivalent to $V_{pmax}$.

# S4. C_3_ A-C_i_ Fitting Results

## S4.1 Limiting Factors For One Curve

Table **S1** below shows limiting factors at each point for the same curve in Figure **1**, as determined by fits from each tool. For *PCE calculator*, the limiting processes were chosen manually. The *PhotoGEA*, *plantecophys*, and *photosynthesis* tools include $A_{c}$, $A_{j}$, $A_{p}$, and $A_{n}$ at each point in the fitting results, so limiting factors were identified by checking whether $A_{n}$ equals $A_{c}$, $A_{j}$, or $A_{p}$. The *msuRACiFit* tool returns a numerical indicator for each point, where values of one, two, or three indicate Rubisco-limited assimilation, RuBP-limited assimilation, or TPU-limited assimilation, respectively.

Note that for the *plantecophys*, and *photosynthesis* tools, these identifications are not necessarily the same ones used for parameter estimation. For this particular curve, the *plantecophys* package used points where $C_{i}$ was below 400 μmol mol^-1^ to estimate $V_{cmax}$ and $R_{L}$, and points where $C_{i}$ was above 400 μmol mol^-1^ to estimate $T_{p}$. The *photosynthesis* package used points where $C_{i}$ was below 543 μmol mol^-1^ to estimate $V_{cmax}$ and $R_{L}$, points where $C_{i}$ was between 543 and 741 μmol mol^-1^ to estimate $J$, and points where $C_{i}$ was above 741 μmol mol^-1^ to estimate $T_{p}$. These $C_{i}$ transition points are reported by *plantecophys* as the *Ci_transition* and *Ci_transition2* outputs, and by *photosynthesis* as the *citransition1* and *citransition2* outputs. For this particular curve, the actual limiting rates in the *photosynthesis* fit are different from the ones used for parameter estimation; this is an example of an “inadmissible fit” (Gu et al. 2010). Across all thirty-six tobacco *A*-*C_i_* curves, the *photosynthesis* and *plantecophys* tools produced 19 and 30 inadmissible fits, respectively. The *plantecophys* tool returned inadmissible fits for 17 of the 18 curves with $Q_{in}$ at or below 300 μmol m^-2^ s^-1^. In contrast, the *photosynthesis* tool returned inadmissible fits for 16 of the 18 curves with $Q_{in}$ below 300 μmol m^-2^ s^-1^, showing that each tool tends to produce these fits in different situations.

There are also subtleties related to the *msuRACiFit* results. For this tool, during the fit, the limiting process is selected by choosing the smallest potential carboxylation rate. Yet, the numerical limiting-process indicator is calculated after the fitting procedure by choosing the smallest potential assimilation rate. Whenever $C_{i}$ is below $\Gamma^{*}$, these two methods always disagree (Supplemental Section **S1.3**) (Lochocki and McGrath 2025). Thus, the three points at low $C_{i}$ where $A_{n}=A_{j}$ in the *msuRACiFit* results were not actually used to estimate $J$ during the fit.

| **Measured** | | **Limiting process determined from fit (**$\boldsymbol{A}_{\boldsymbol{n}}\boldsymbol{=?}$**)** | | | | | |
| --- | --- | --- | --- | --- | --- | --- | --- |
| $C_{i}$  μmol mol^-1^ | $A_{n}$  μmol m^-2^ s^-1^ | *PhotoGEA (*$\alpha_{old}$*)* | *PhotoGEA (*$\alpha_{G},\alpha_{S}$*)* | *plantecophys* | *photosynthesis* | *msuRACiFit* | *PCE calculator* |
| 15.9 | -1.31 | $A_{c}$ | $A_{c}$ | $A_{c}$ | $A_{c}$ | $A_{j}$ | $A_{c}$ |
| 26.4 | -1.43 | $A_{c}$ | $A_{c}$ | $A_{c}$ | $A_{c}$ | $A_{j}$ | $A_{c}$ |
| 36.1 | -1.40 | $A_{c}$ | $A_{c}$ | $A_{c}$ | $A_{c}$ | $A_{j}$ | $A_{c}$ |
| 43.8 | -0.98 | $A_{c}$ | $A_{c}$ | $A_{c}$ | $A_{c}$ | $A_{c}$ | $A_{c}$ |
| 50.3 | -0.33 | $A_{c}$ | $A_{c}$ | $A_{c}$ | $A_{c}$ | $A_{c}$ | $A_{c}$ |
| 66.1 | 1.31 | $A_{c}$ | $A_{c}$ | $A_{c}$ | $A_{c}$ | $A_{c}$ | $A_{c}$ |
| 80.9 | 3.04 | $A_{c}$ | $A_{c}$ | $A_{c}$ | $A_{c}$ | $A_{c}$ | $A_{c}$ |
| 111. 8 | 6.19 | $A_{c}$ | $A_{c}$ | $A_{c}$ | $A_{c}$ | $A_{c}$ | $A_{c}$ |
| 141.6 | 9.20 | $A_{c}$ | $A_{c}$ | $A_{c}$ | $A_{c}$ | $A_{c}$ | $A_{c}$ |
| 202.3 | 14.93 | $A_{c}$ | $A_{c}$ | $A_{c}$ | $A_{c}$ | $A_{c}$ | $A_{c}$ |
| 267.5 | 19.82 | $A_{c}$ | $A_{c}$ | $A_{c}$ | $A_{c}$ | $A_{c}$ | $A_{c}$ |
| 366.8 | 26.47 | $A_{c}$ | $A_{c}$ | $A_{c}$ | $A_{c}$ | $A_{c}$ | $A_{c}$ |
| 448.2 | 30.13 | $A_{j}$ | $A_{j}$ | $A_{p}$ | $A_{p}$ | $A_{c}$ | $A_{j}$ |
| 639.1 | 31.62 | $A_{p}$ | $A_{p}$ | $A_{p}$ | $A_{p}$ | $A_{p}$ | $A_{p}$ |
| 843.7 | 29.75 | $A_{p}$ | $A_{p}$ | $A_{p}$ | $A_{p}$ | $A_{p}$ | $A_{p}$ |
| 1045.3 | 28.18 | $A_{p}$ | $A_{p}$ | $A_{p}$ | $A_{p}$ | $A_{p}$ | $A_{p}$ |
| 1339.6 | 27.38 | $A_{p}$ | $A_{p}$ | $A_{p}$ | $A_{p}$ | $A_{p}$ | $A_{p}$ |

Table S1: Limiting factors determined for a single C_3_ A-Ci curve (designated “800 – wt-5 – mcgrath1”). For each point in the curve, the table includes the measured values of $A_{n}$ and $C_{i}$ along with the limiting process identified by each fitting tool (PhotoGEA, plantecophys, photosynthesis, msuRACiFit, and PCE calculator).

## S4.2 Fits from Each Tool

Figures **S1-S4** below the fits of each tobacco *A*-*C_i_* curve as calculated using *PhotoGEA*, *plantecophys*, *photosynthesis*, and *msuRACiFit*. Individual fits were generally not retained when using *PCE calculator*, following common practice across the literature; the one available fit is shown in Figure **1** of the main text.


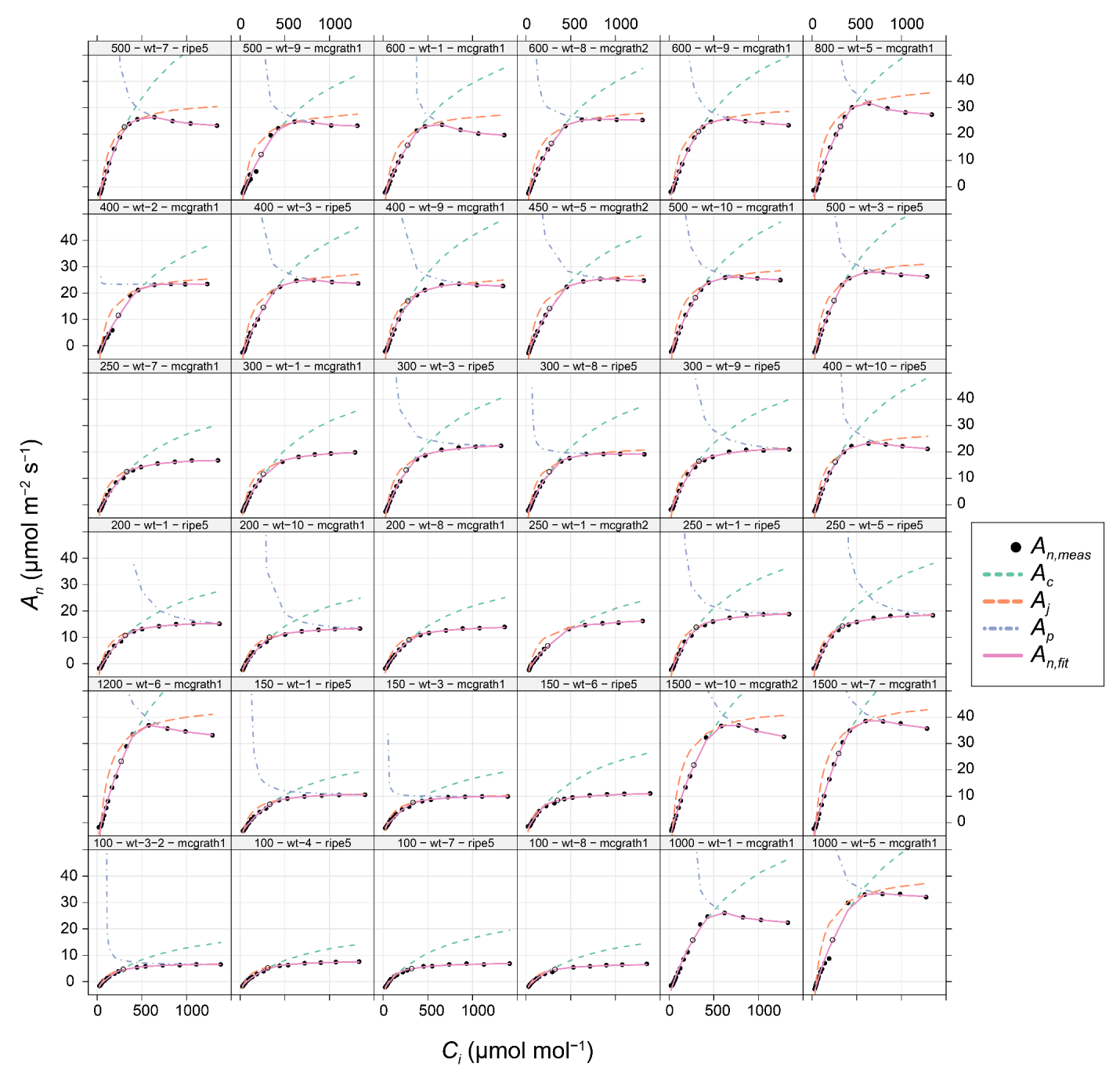


Figure S1: Fits of all thirty-six tobacco A-C_i_ curves made using PhotoGEA (allowing $\alpha_{old}$ to vary). All lines are composed of straight segments connecting adjacent points and are intended only as guides to the eye. Fitted assimilation rates are shown as lines, measured points are shown as filled black circles, and estimated operating points are shown as open black circles.


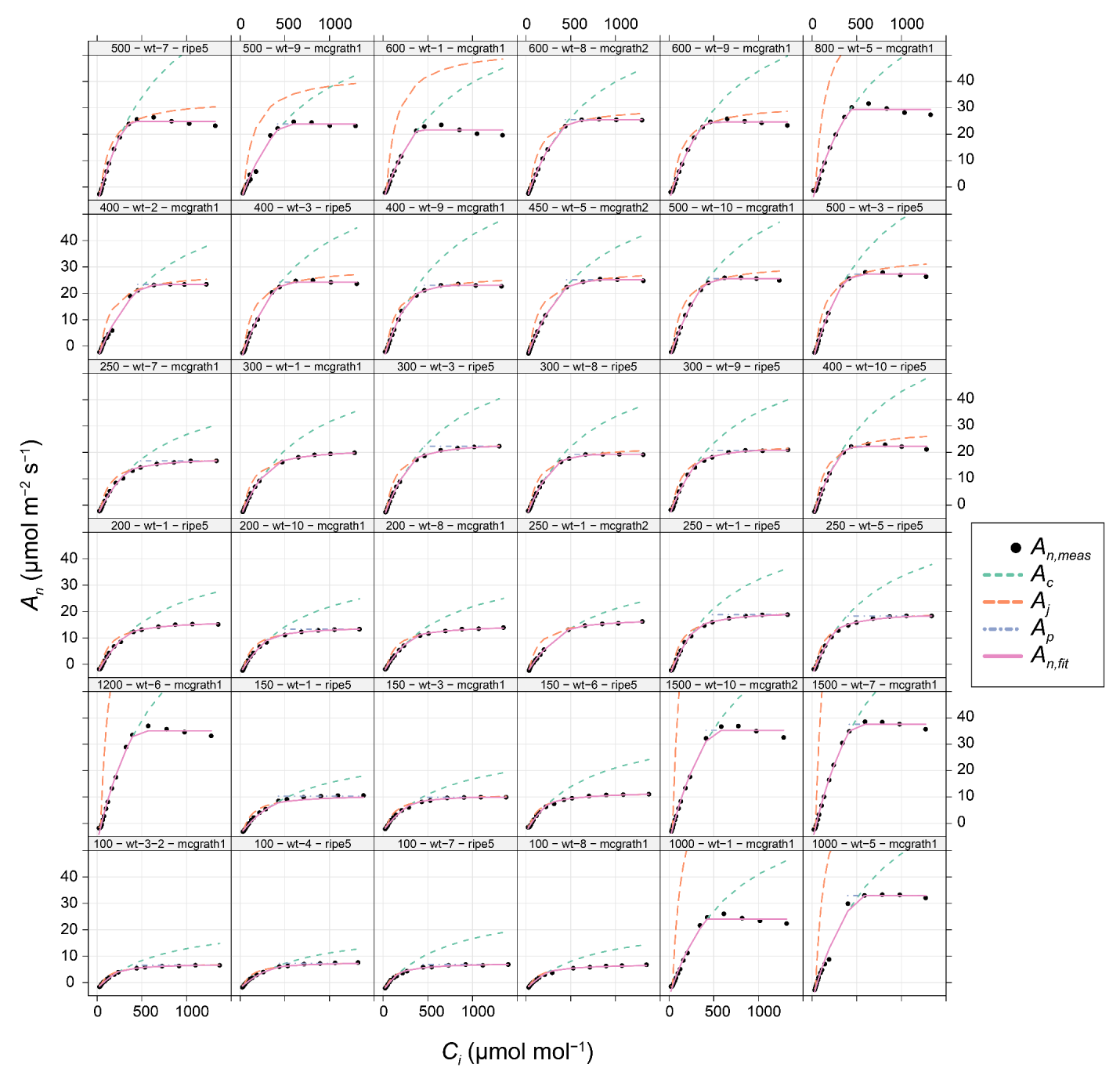


Figure S2: Fits of all thirty-six tobacco A-C_i_ curves made using plantecophys. All lines are composed of straight segments connecting adjacent points and are intended only as guides to the eye. Fitted assimilation rates are shown as lines and measured points are shown as filled black circles.


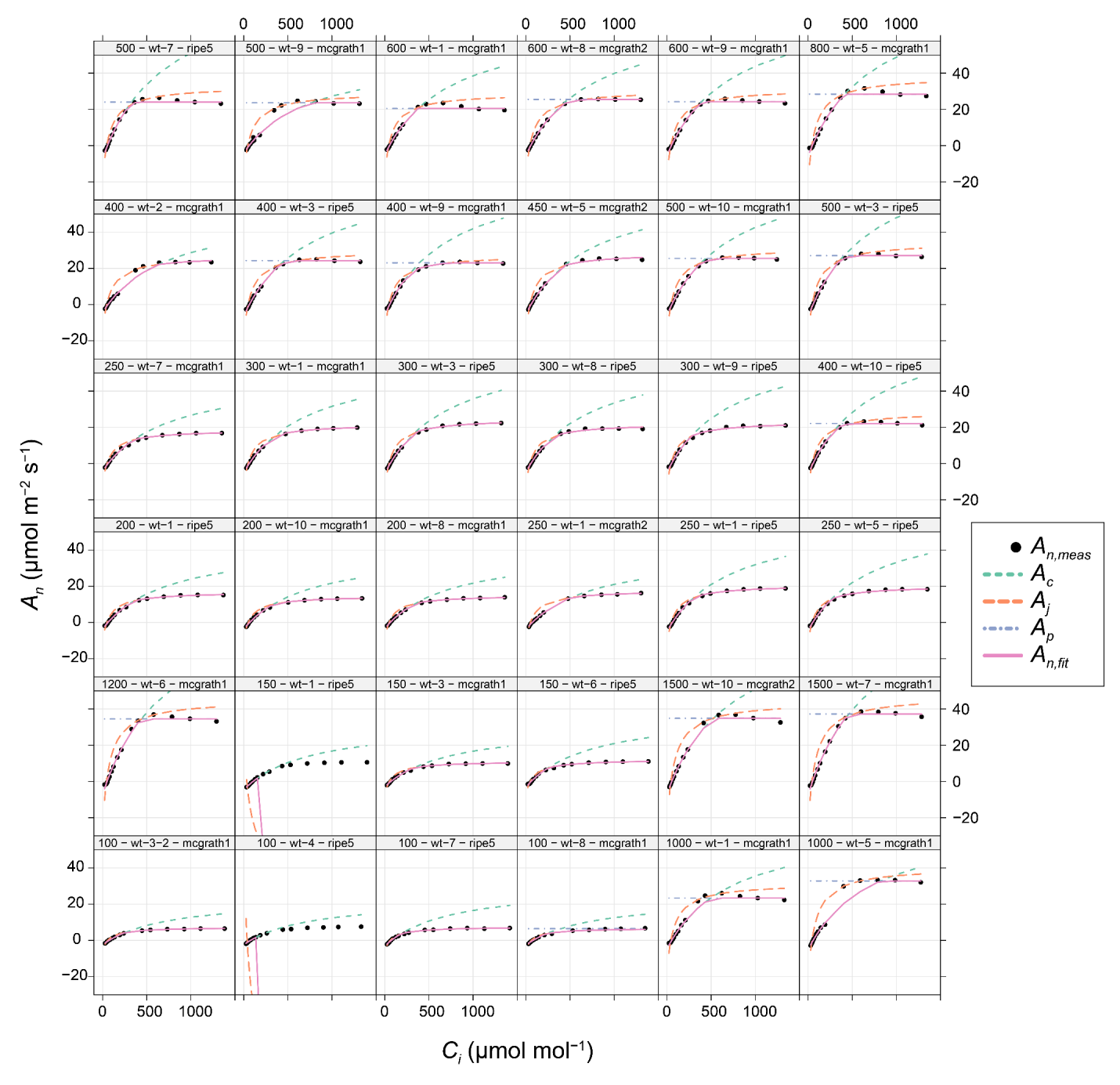


Figure S3: Fits of all thirty-six tobacco A-C_i_ curves made using photosynthesis. All lines are composed of straight segments connecting adjacent points and are intended only as guides to the eye. Fitted assimilation rates are shown as lines and measured points are shown as filled black circles.


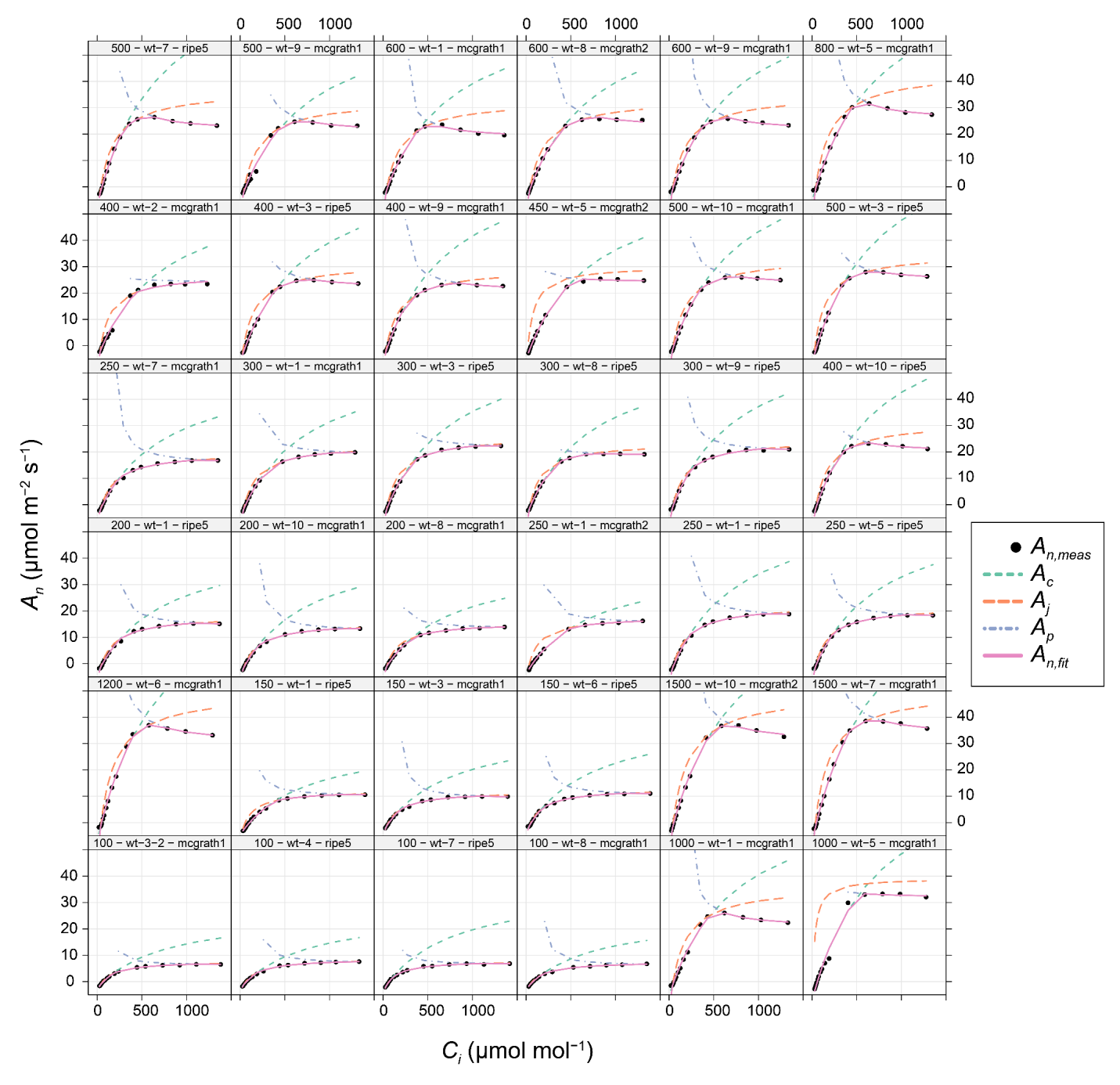


Figure S4: Fits of all thirty-six tobacco A-C_i_ curves made using msuRACiFit. All lines are composed of straight segments connecting adjacent points and are intended only as guides to the eye. Fitted assimilation rates are shown as lines and measured points are shown as filled black circles.

## S4.3 Each Curve


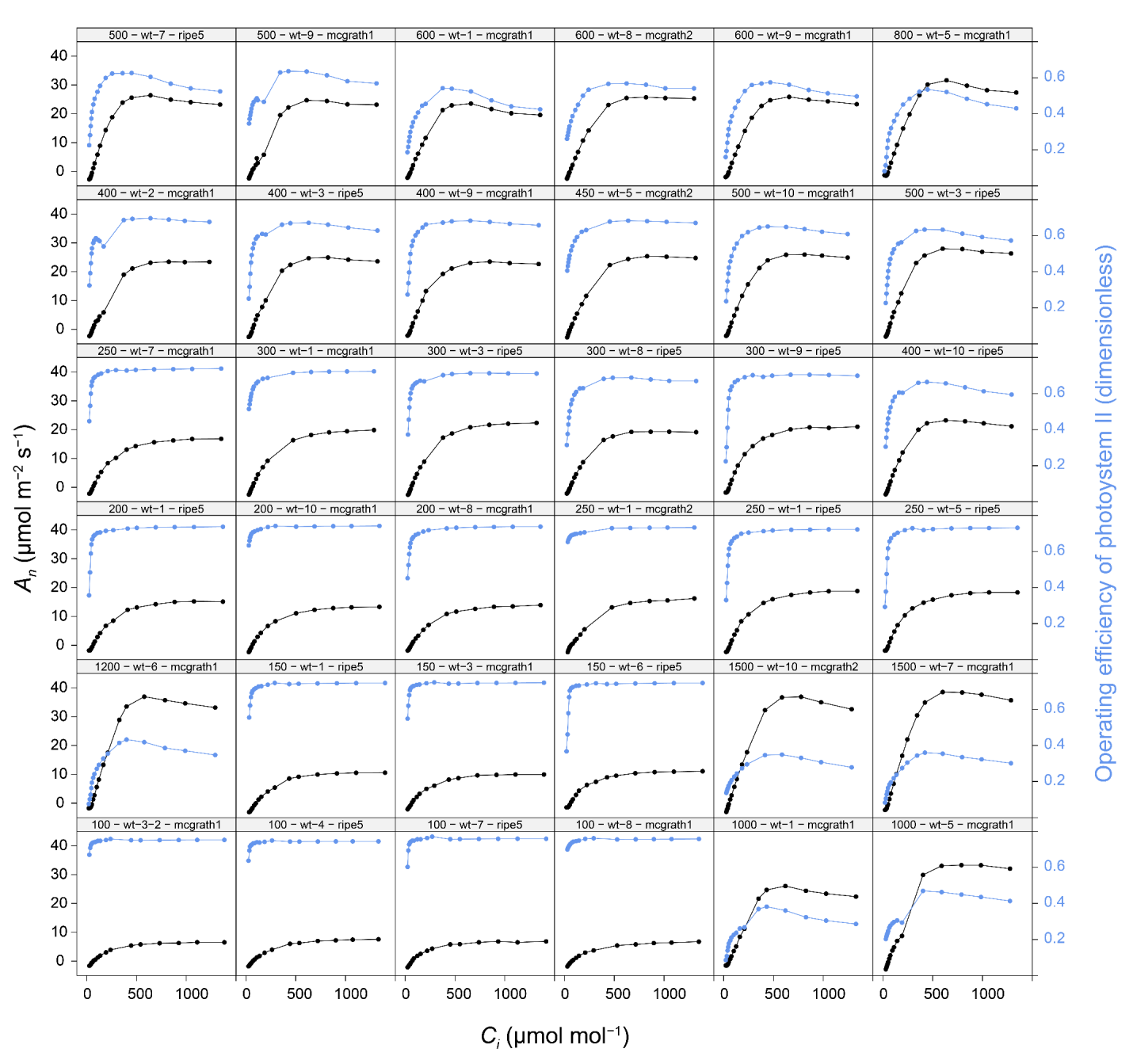


Figure S5: Measured values of $A_{n}$ (filled black circles) and $\phi_{PSII}$ (filled blue circles) vs $C_{i}$ for all thirty-six tobacco A-C_i_ curves. All lines are composed of straight segments connecting adjacent points and are intended only as guides to the eye.

## S4.4 Comparison of Akiake Information Criterion (AIC) values

The AIC was calculated for each fit from each package using the *residual_stats* function from *PhotoGEA*, which implements the equation just above Equation 6 in Banks and Joyner (2017).


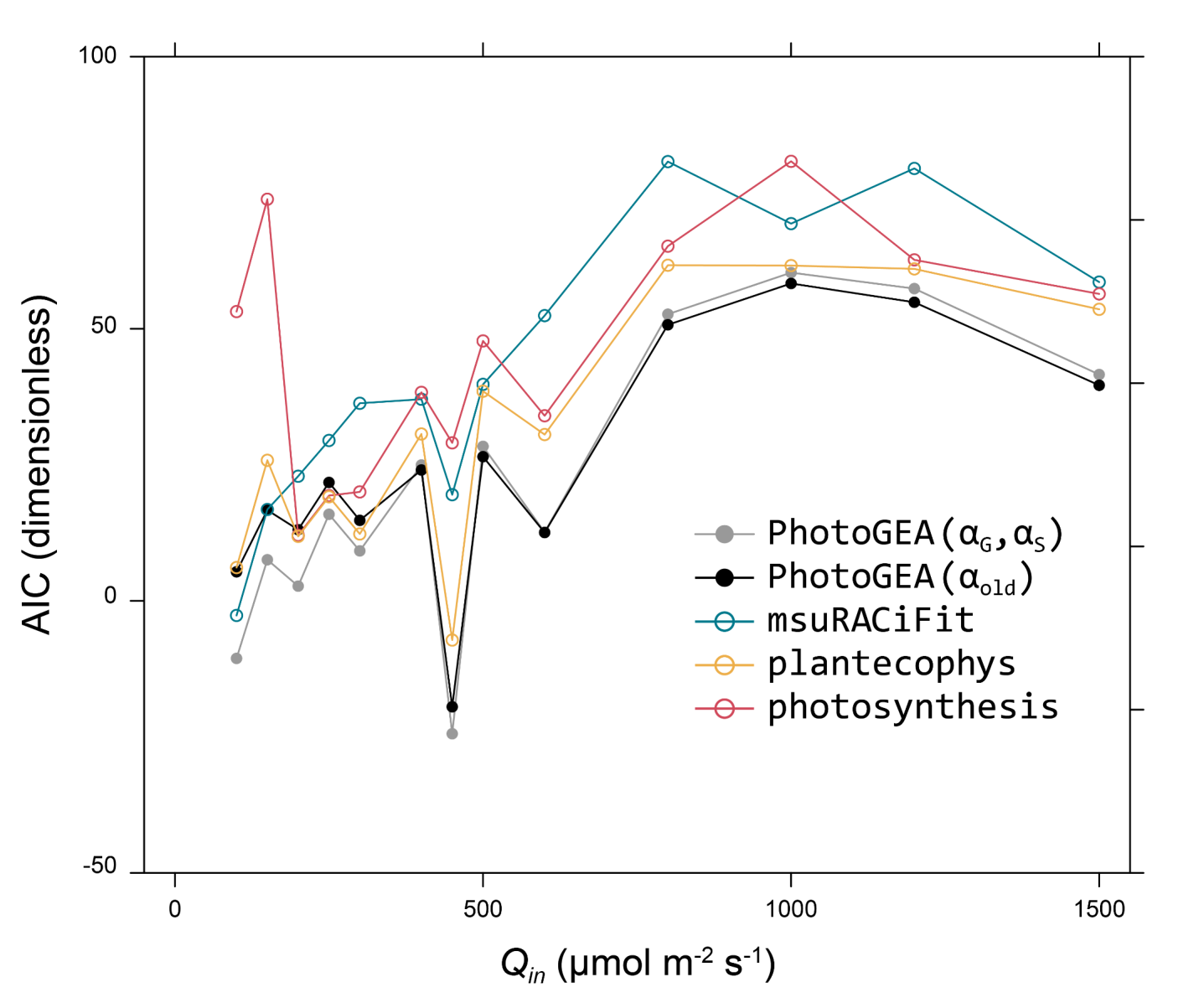


Figure S6: Average Akaike information criteria (AIC) values vs. $Q_{in}$ for the thirty-six tobacco A-C_i_ curves.

## S4.5 Comparison of Estimated α Values


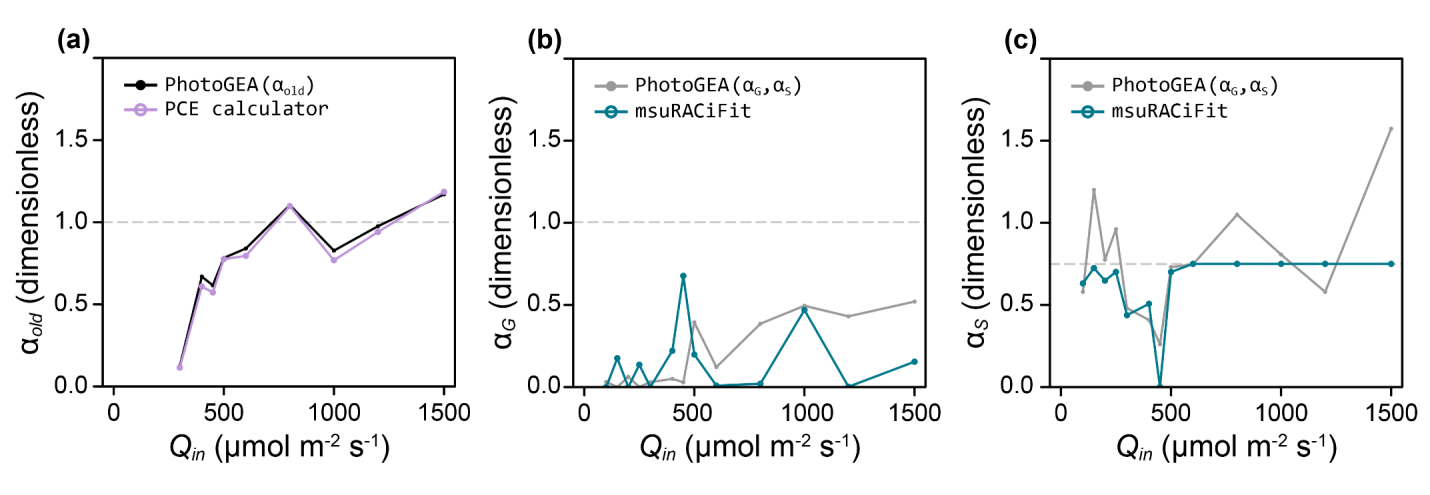


Figure S7: Fitting thirty-six C_3_ A-C_i_ curves measured at a range of $Q_{in}$ values with several different software tools. (**a-c**) Average best-fit values of $\alpha_{old}$, $\alpha_{G}$, and $\alpha_{S}$, respectively, vs. $Q_{in}$, as determined by each fitting tool. Each point is an average value from 1-4 response curve fits. Statistical error bars are excluded for clarity, since they are similar across all fitting tools. Theoretical upper limits for each parameter are shown as horizontal dashed lines at $\alpha_{old}$ = 1, $\alpha_{G}$ = 1, and$\alpha_{S}$ = 0.75. Note that $\alpha_{G}$ and $\alpha_{S}$ are theoretically constrained to ${0\leq\alpha}_{G}+\frac{4\alpha_{S}}{3}\leq1$ (Busch, Sage, and Farquhar 2018; Busch 2020), so $\alpha_{G}$ is only expected to reach 1 when $\alpha_{S}$ is zero, and $\alpha_{S}$ is only expected to reach 0.75 when $\alpha_{G}$ is zero.

## S4.6 Comparison of Estimated *J* and *T_p_* Values from Each Curve


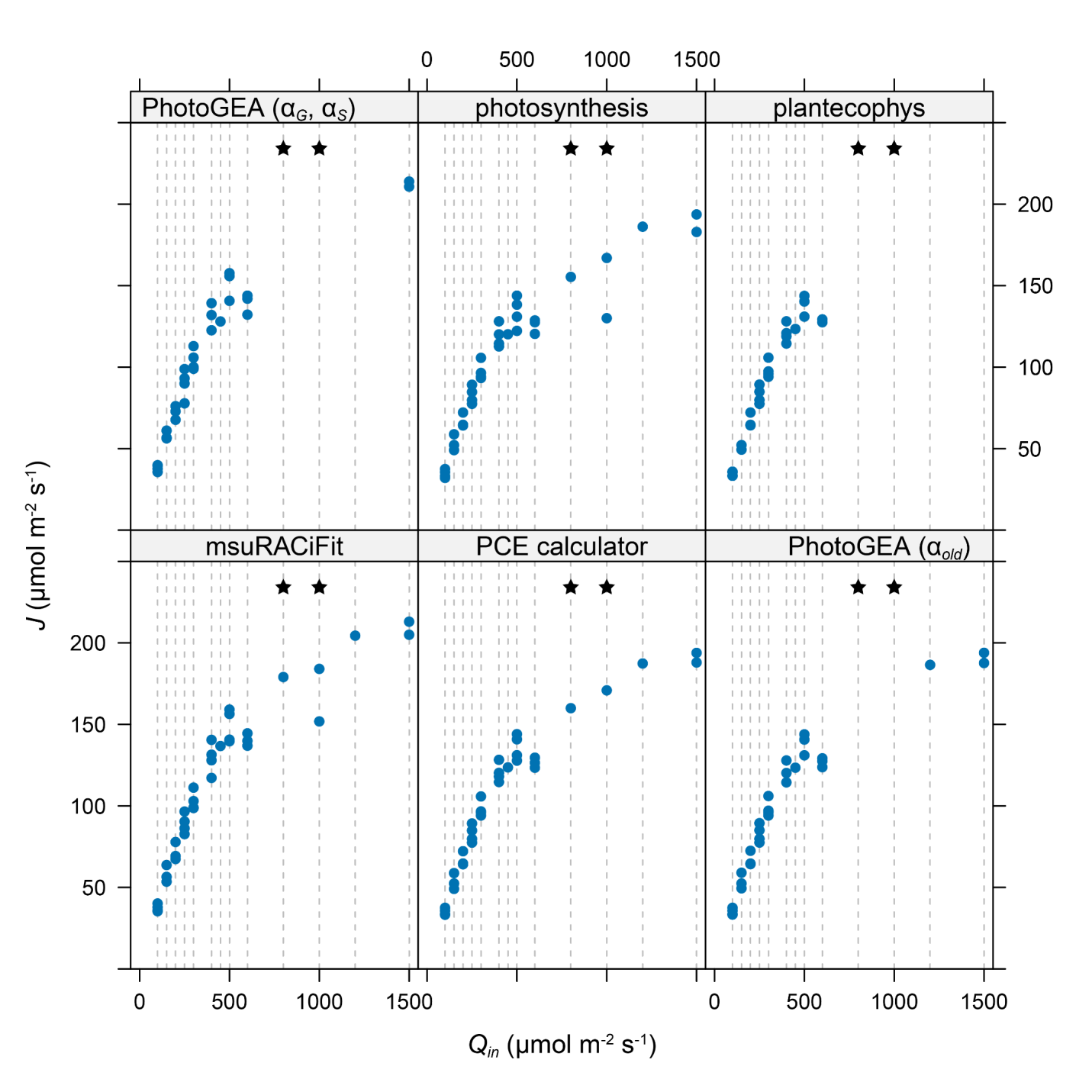


Figure S8: Values of apparent $J$ (filled blue circles) estimated from each of 36 tobacco A-C_i_ curves, plotted against $Q_{in}$ and grouped by fitting tool. Gray vertical dashed lines indicate each $Q_{in}$ value where curves were measured. Filled black stars indicate values of $Q_{in}$ where PhotoGEA ($\alpha_{old}$) indicated that reliable $J$ estimates were not possible.


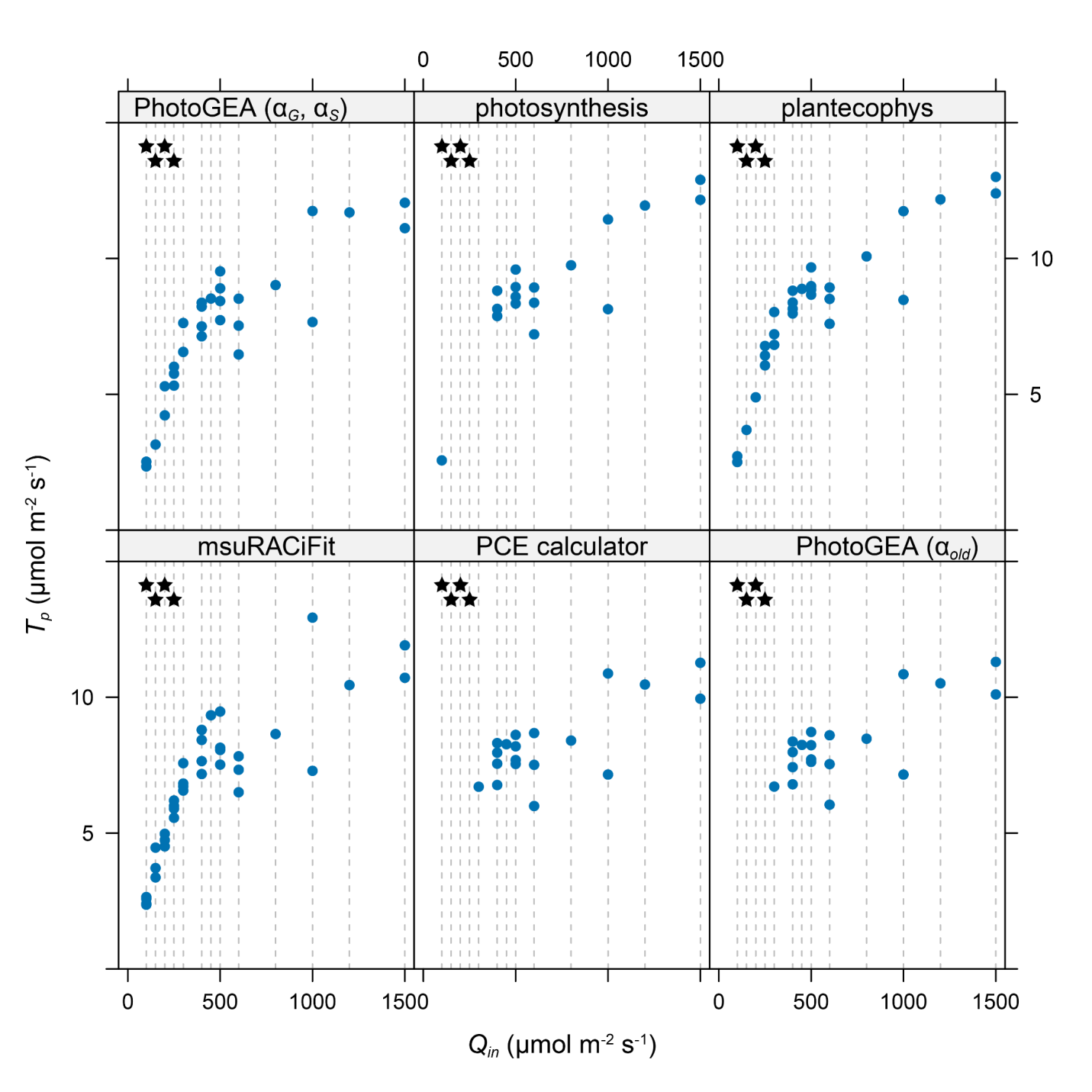


Figure S9: Values of apparent $T_{p}$ (filled blue circles) estimated from each of 36 tobacco A-C_i_ curves, plotted against $Q_{in}$ and grouped by fitting tool. Gray vertical dashed lines indicate each $Q_{in}$ value where curves were measured. Filled black stars indicate values of $Q_{in}$ where PhotoGEA ($\alpha_{old}$) indicated that reliable $T_{p}$ estimates were not possible.

# S5. C_3_ *A*-*C_i_* + CF Variable *J* Fitting Results

The figures below show the measured soybean curves (Figure **S10**), the curve fits on a $C_{i}$ basis (Figure **S11**), the estimated $g_{mc}$ values (Figure **S12**), and the estimated $J_{actual}$ values (Figure **S13**).

The expected relationship between $J$ and $J_{actual}$ is discussed in the main text (following Equation **2** in Section **3.4**), and examined for a single curve in Figure **4d**. In Figure **S13**, it can be seen that all curves exhibit $J_{actual}\leq J$, with equality (or near equality) only occurring when assimilation is limited by RuBP regeneration. This is a consistency check, indicating that the fits are biologically reasonable.

Note that to create the average curve in Figure **3e** of the main text, the curve designated “2021 – ripe3 – 4” was excluded because its $C_{i}$ values were substantially different than the other curves, and the curve designated “2022 – ripe2 – 5” was excluded because its $g_{mc}$ values were outliers.


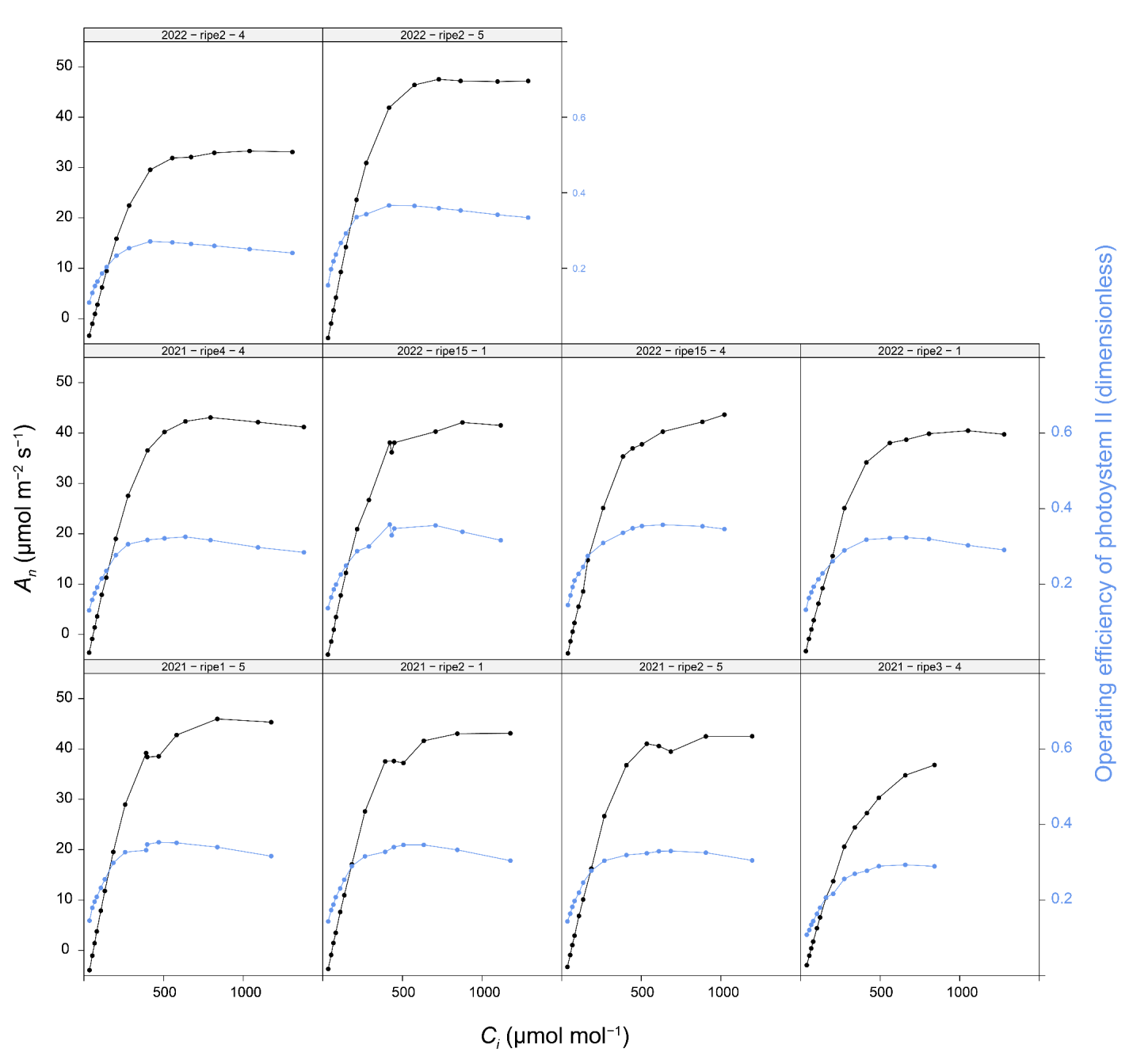


Figure S10: Measured values of $A_{n}$ (filled black circles) and $\phi_{PSII}$ (filled blue circles) vs $C_{i}$ for all ten soybean A-C_i_ +CF curves. All lines are composed of straight segments connecting adjacent points and are intended only as guides to the eye.


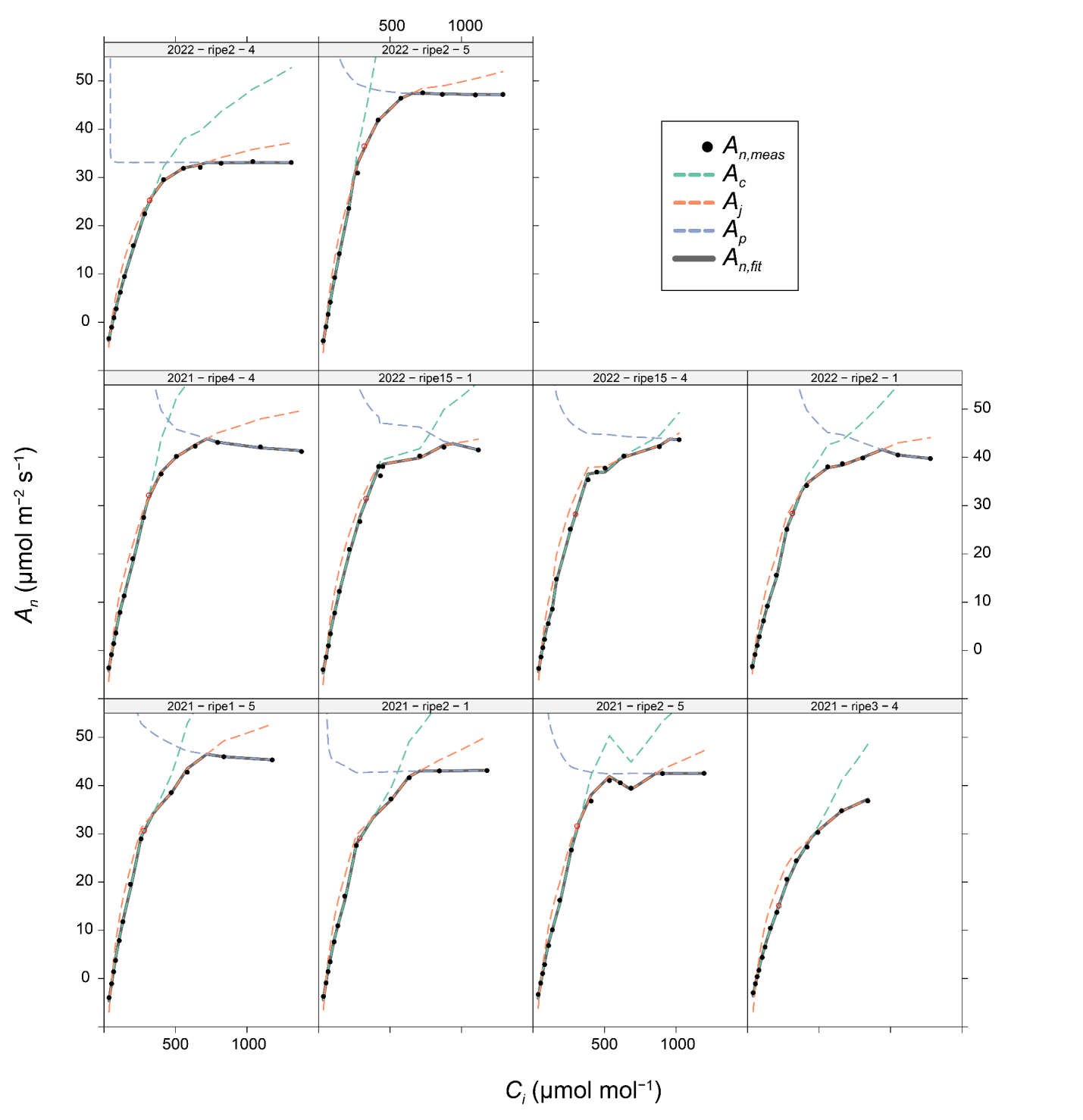


Figure S11: Assimilation rates estimated from Variable J fits of ten soybean A-C_i_ +CF curves made using PhotoGEA (allowing $\alpha_{old}$ to vary). All lines are composed of straight segments connecting adjacent points and are intended only as guides to the eye. Fitted assimilation rates are shown as lines, measured points are shown as filled black circles, and estimated operating points are shown as open red circles.


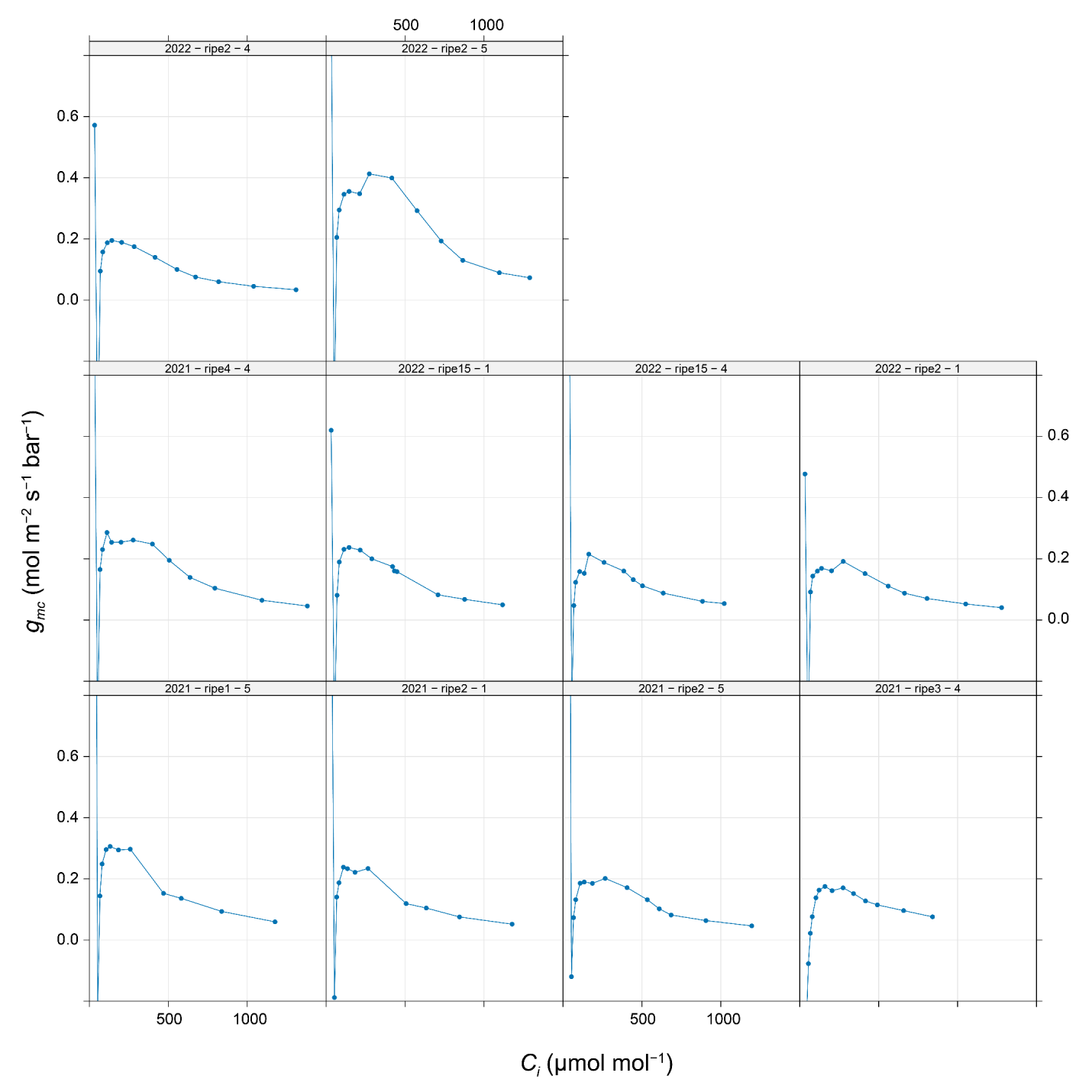


Figure S12: Values of $g_{mc}$ estimated from Variable J fits of ten soybean A-C_i_ +CF curves made using PhotoGEA (allowing $\alpha_{old}$ to vary). All lines are composed of straight segments connecting adjacent points and are intended only as guides to the eye.


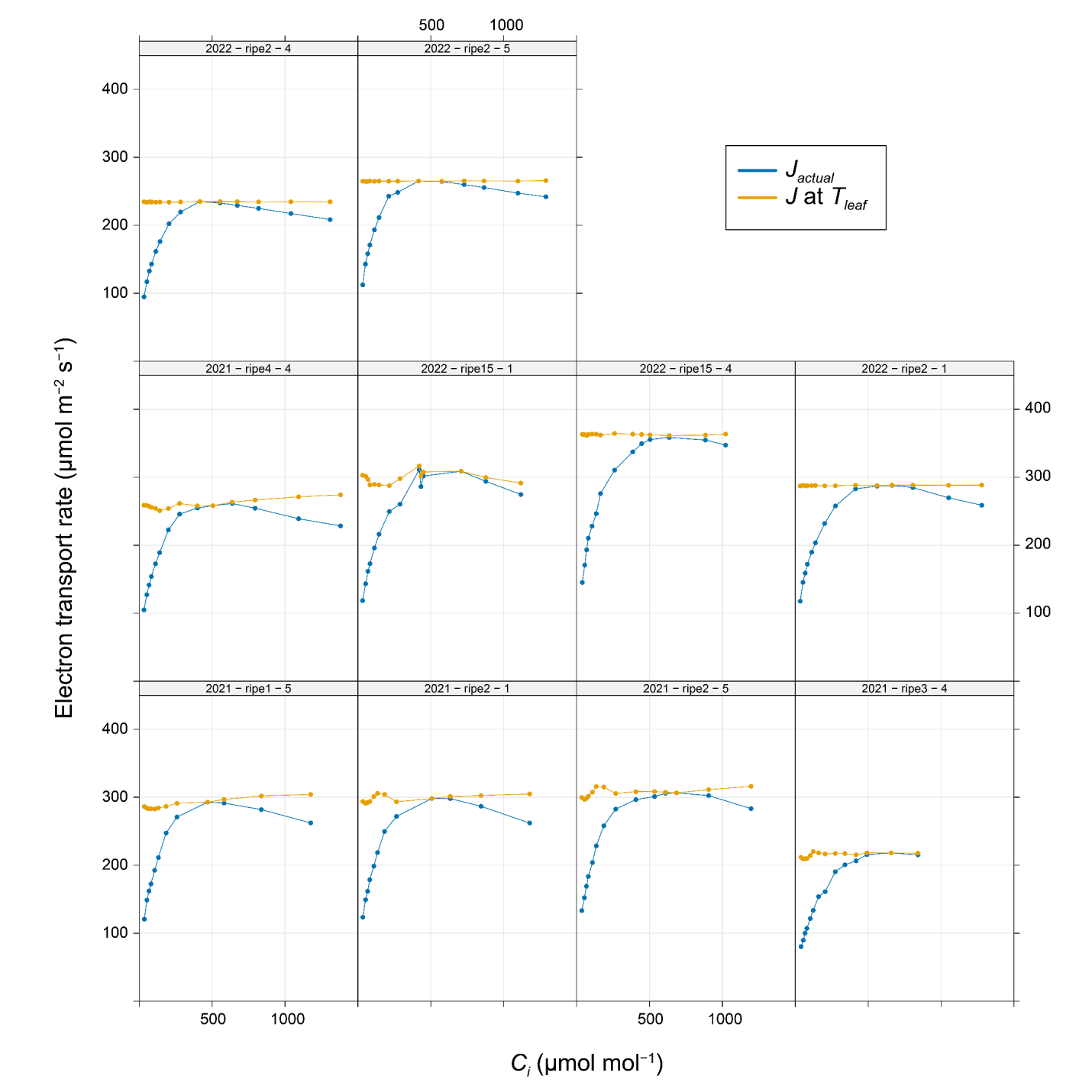


Figure S13: Values of $J_{actual}$ and $J$ at leaf temperature estimated from Variable J fits of ten soybean A-C_i_ +CF curves made using PhotoGEA (allowing $\alpha_{old}$ to vary). All lines are composed of straight segments connecting adjacent points and are intended only as guides to the eye.

# S6. C_4_ *A*-*C_i_* Fitting Results

## S6.1 Correlations

Table **S2** below shows the detailed fitting results for the correlation lines in Figure **5e-g** of the main text. Note that results from one curve (designated “sorghum – ripe14 – 4 – 2022”) are excluded from Figure **5g** of the main text (and the corresponding correlation fits) because its $V_{pmax}$ estimates were outliers.

| **Species** | **Y** | **X** | **intercept** | **slope** | **R^2^** | ***p*-value** |
| --- | --- | --- | --- | --- | --- | --- |
| Maize | $V_{cmax}$  at 25 °C | $V_{max}$ | -1.4 ± 6.8 | 0.65 ± 0.13 | 0.73 | 7.64E-04 |
|  | $J$  at 25 °C | $V_{max}$ | -22 ± 38 | 3.7 ± 0.7 | 0.75 | 6.11E-04 |
|  | $V_{pmax}$  at 25 °C (Rubisco) | $V_{pmax}$  (low $C_{i}$) | -16 ± 16 | 2.6 ± 0.3 | 0.92 | 3.67E-06 |
|  | $V_{pmax}$  at 25 °C (light) | $V_{pmax}$  (low $C_{i}$) | -33 ± 19 | 1.9 ± 0.3 | 0.83 | 9.38E-05 |
| Sorghum | $V_{cmax}$  at 25 °C | $V_{max}$ | -20 ± 22 | 0.90 ± 0.39 | 0.37 | 4.81E-02 |
|  | $J$  at 25 °C | $V_{max}$ | -26 ± 78 | 4.4 ± 1.4 | 0.52 | 1.14E-02 |
|  | $V_{pmax}$  at 25 °C (Rubisco) | $V_{pmax}$  (low $C_{i}$) | -126 ± 24 | 3.9 ± 0.3 | 0.94 | 2.63E-06 |
|  | $V_{pmax}$  at 25 °C (light) | $V_{pmax}$  (low $C_{i}$) | -132 ± 29 | 3.3 ± 0.4 | 0.89 | 4.53E-05 |

Table S2: Detailed fitting results, where a linear equation Y = intercept + slope * X was fit to several data sets. In all cases, the X variable, Y variable, and intercept have units of μmol m^-2^ s^-1^, while the slope is dimensionless.

## S6.2 Fits at Low C_i_


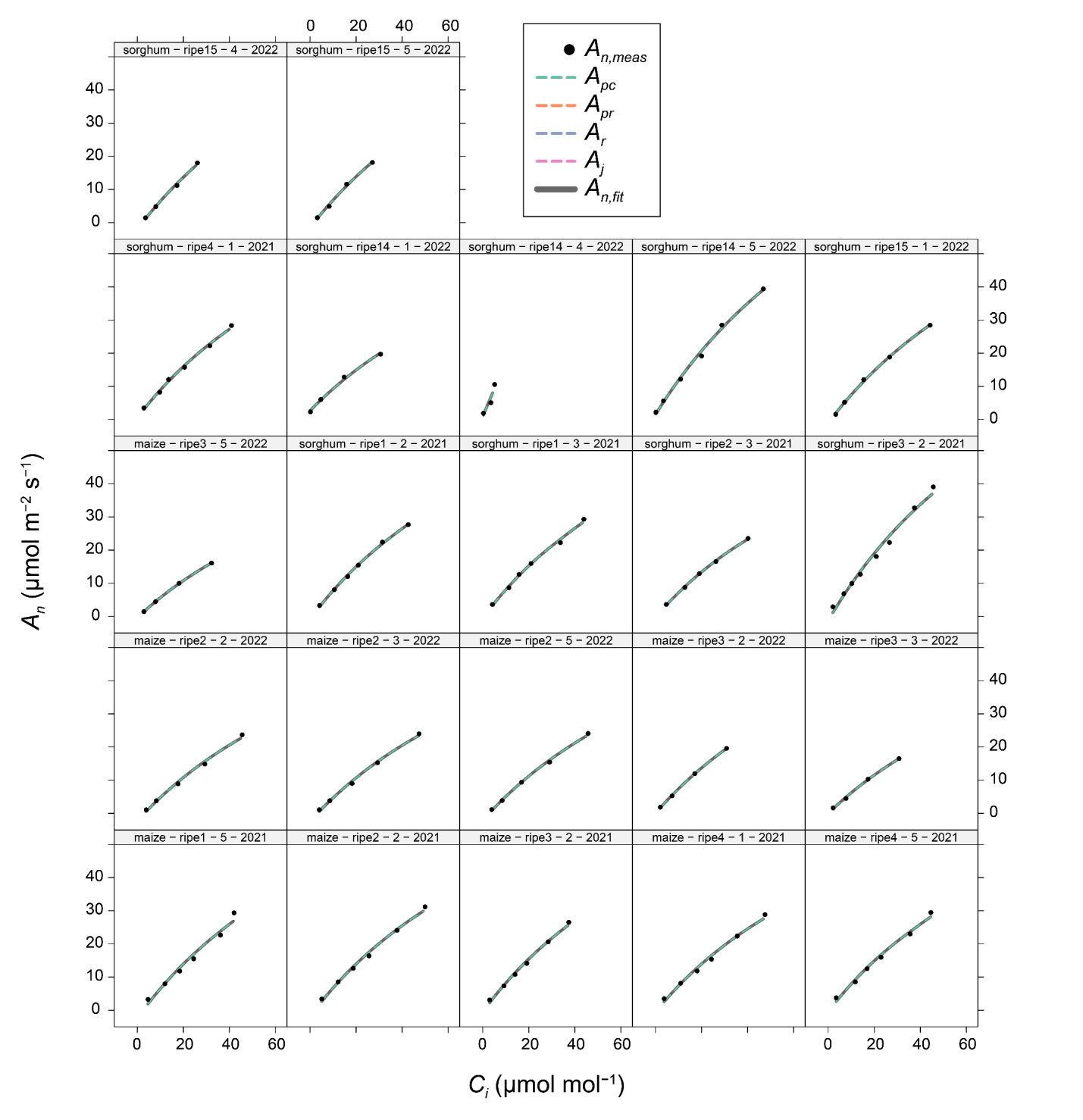


Figure S14: Fits of all eleven maize and eleven sorghum A-C_i_ curves made using PhotoGEA, fitting the mechanistic C_4_ model at low $C_{i}$ (≤ 60 μmol mol^-1^). All lines are composed of straight segments connecting adjacent points and are intended only as guides to the eye. Fitted assimilation rates are shown as lines and measured points are shown as filled black circles.

## S6.3 Fits with the Empirical Hyperbola


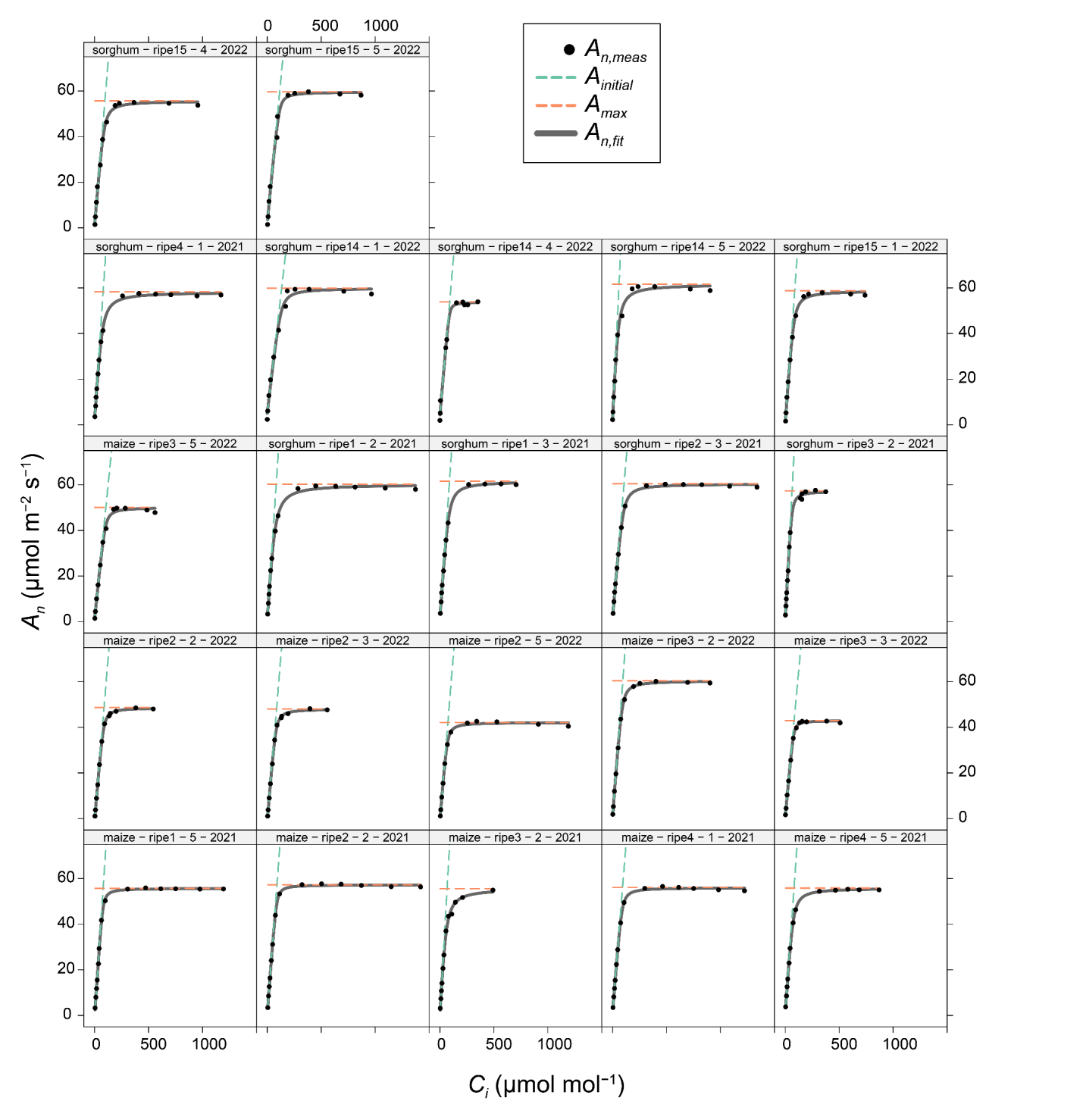


Figure S15: Fits of all eleven maize and eleven sorghum A-C_i_ curves made using PhotoGEA, fitting the empirical hyperbolic model. All lines are composed of straight segments connecting adjacent points and are intended only as guides to the eye. Fitted assimilation rates are shown as lines and measured points are shown as filled black circles.

## S6.4 Fits Assuming Rubisco Limitations


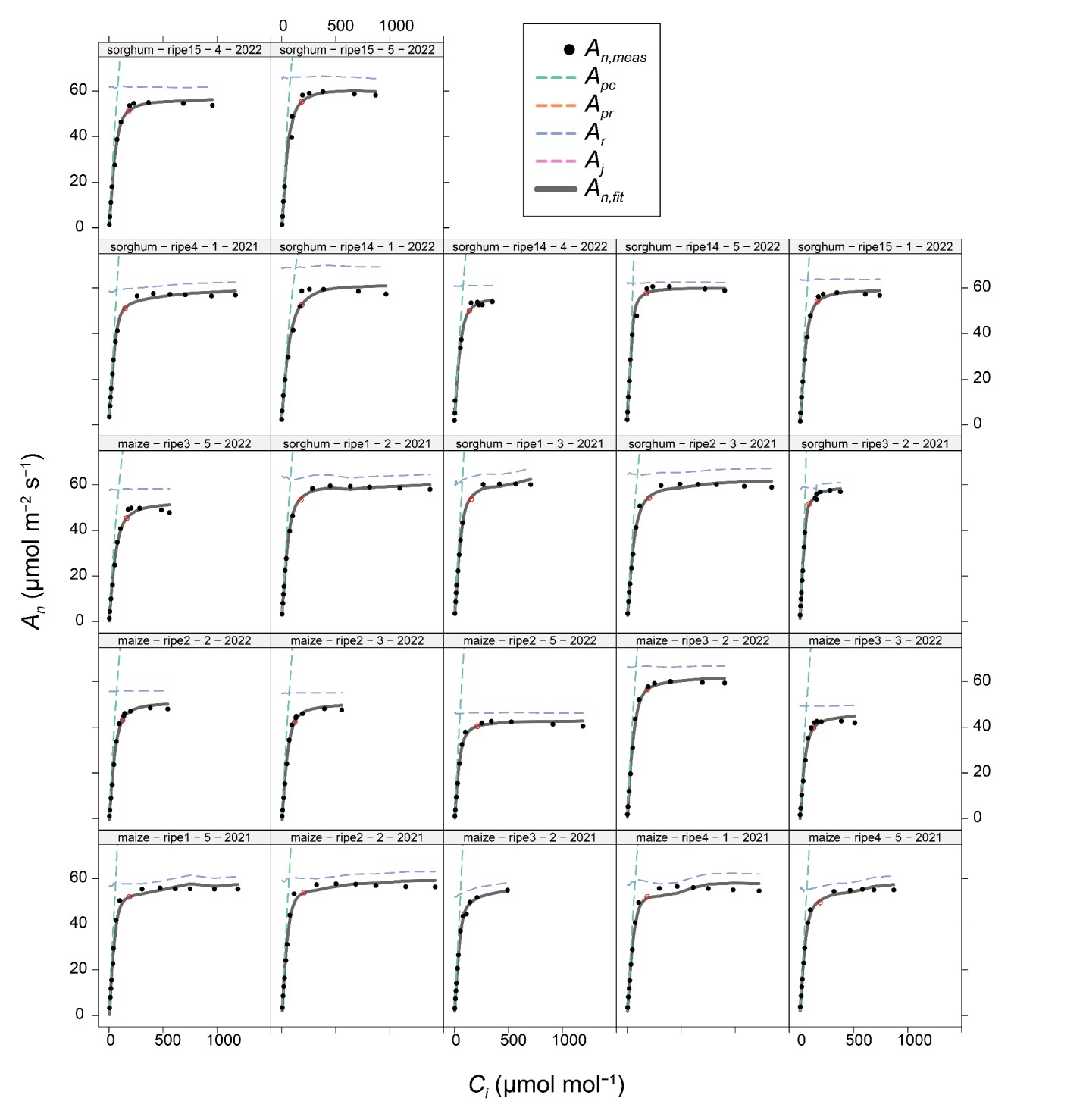


Figure S16: Fits of all eleven maize and eleven sorghum A-C_i_ curves made using PhotoGEA, fitting the mechanistic C_4_ model assuming Rubisco limitations. All lines are composed of straight segments connecting adjacent points and are intended only as guides to the eye. Fitted assimilation rates are shown as lines, measured points are shown as filled black circles, and estimated operating points are shown as open red circles.

## S6.5 Fits Assuming Light Limitations


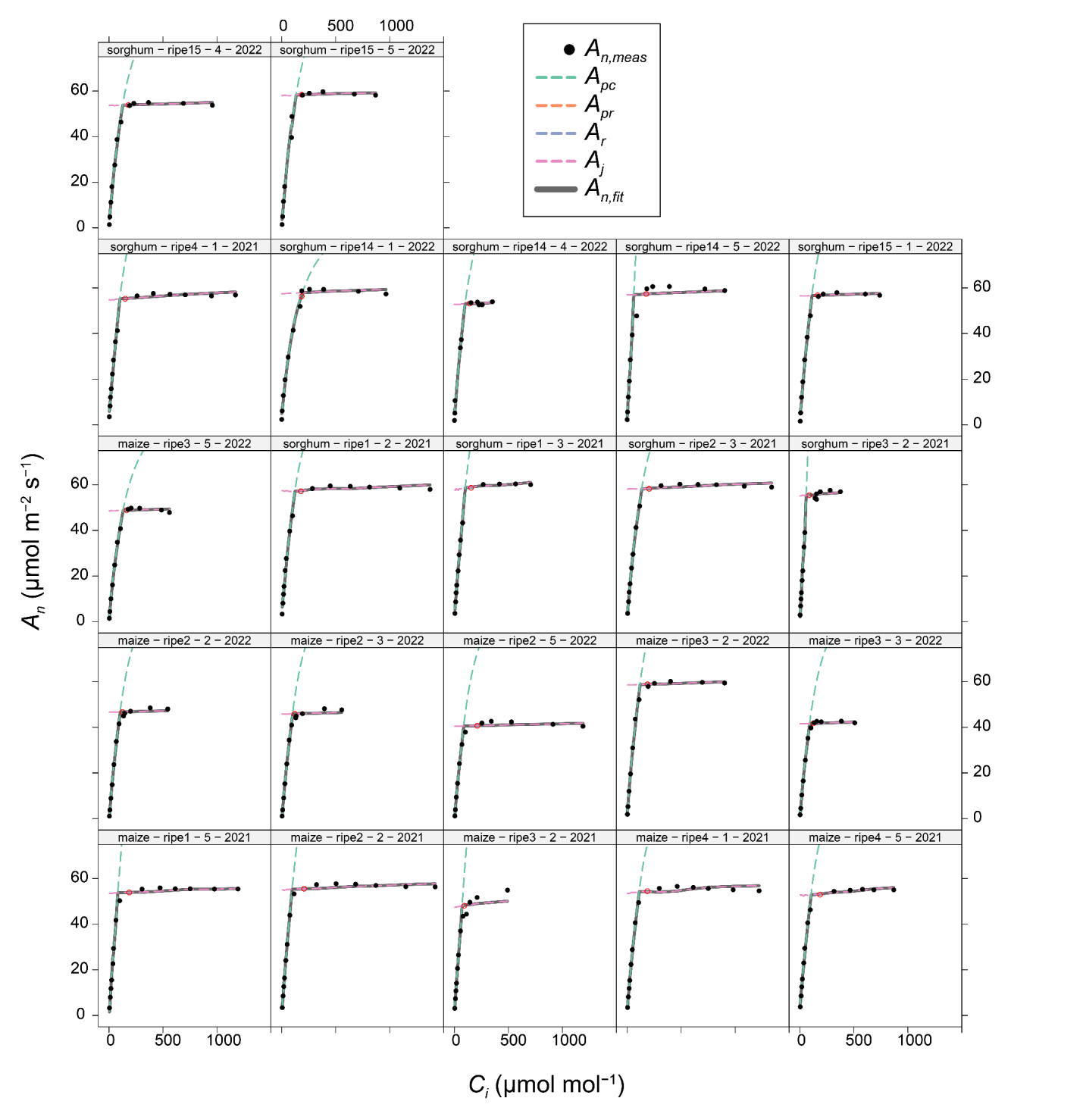


Figure S17: Fits of all eleven maize and eleven sorghum A-C_i_ curves made using PhotoGEA, fitting the mechanistic C_4_ model assuming light limitations. All lines are composed of straight segments connecting adjacent points and are intended only as guides to the eye. Fitted assimilation rates are shown as lines, measured points are shown as filled black circles, and estimated operating points are shown as open red circles.

## S6.6 Each Curve


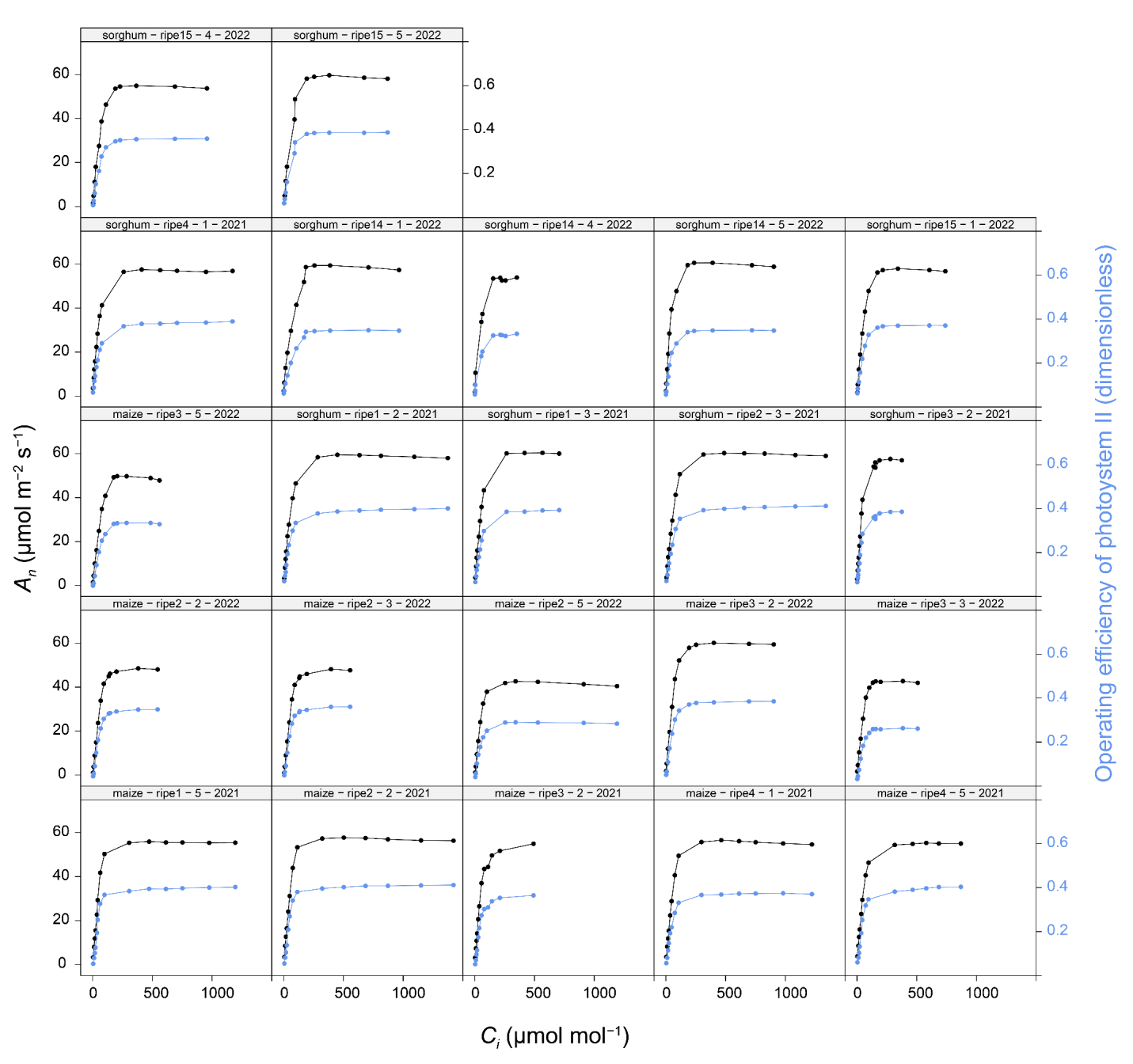


Figure S18: Measured values of $A_{n}$ (filled black circles) and $\phi_{PSII}$ (filled blue circles) vs $C_{i}$ for all eleven maize and eleven sorghum A-C_i_ curves. All lines are composed of straight segments connecting adjacent points and are intended only as guides to the eye.

# S7. Temperature Response in *PhotoGEA*

When fitting a mechanistic model to a CO_2_ response curve with *PhotoGEA*, the values of key photosynthetic parameters at the leaf temperature ($T_{leaf}$) are determined from their corresponding values at a reference temperature ($T_{ref}$) according to

$P_{T_{leaf}}=P_{T_{ref}}\cdot F\left( T_{leaf} \right)$, (**D1**)

where $P_{T_{leaf}}$ and $P_{T_{ref}}$ are a parameter’s value at the leaf and reference temperature, respectively, and $F\left( T_{leaf} \right)$ is the parameter’s normalized temperature response function, such that $F=1$ at the reference temperature. Common temperature responses include Arrhenius and Gaussian responses, given by

$F\left( T_{leaf} \right)=e^{c-E_{a}/\left( R\cdot T_{k} \right)}$ (**D2**)

and

$F\left( T_{leaf} \right)={c\cdot e}^{-\left( T_{leaf}-T_{opt} \right)^{2}/W^{2}}$, (**D3**)

respectively, where $c$ is a scaling factor, $E_{a}$ is an activation energy, $R$ is the ideal gas constant, $T_{k}$ is the leaf temperature in Kelvin, $T_{opt}$ is the optimal temperature (where the parameter’s value is maximized), and $W$ is a width. For a normalized Arrhenius response, the value of $c$ must be equal to $E_{a}/\left( R\cdot T_{ref,K} \right)$ to ensure that $F=1$ at the reference temperature, where $T_{ref,K}$ is the reference temperature in Kelvin. For a normalized Gaussian response, the value of $c$ must be equal to $e^{\left( T_{ref}-T_{opt} \right)^{2}/W^{2}}$to ensure that $F=1$ at $T_{ref}$.

*PhotoGEA* does not enforce a particular type of temperature response when fitting, enabling flexibility that may be necessary for certain species; for example, a second-order polynomial response has been used for some cotton parameters (Sargent et al. 2024). *PhotoGEA* currently provides options for several functional forms: Arrhenius, Gaussian, and Johnson-Eyring-Williams (Johnson, Eyring, and Williams 1942). It also includes preset options for widely used temperature response parameterizations (see below). As package development proceeds, more response functions and preset parameterizations will be made available.

To perform a fit, the value at the reference temperature is varied, with the temperature response function being chosen beforehand by the user. While $P_{T_{ref}}$ is taken to be constant across all points in the curve, $T_{leaf}$ is not necessarily constant, so $P_{T_{leaf}}$ may also vary. To account for this, the mean value of $P_{T_{leaf}}$ across a curve is returned at the end of the fit, along with its value at each point.

For C_3_ *A*-*C_i_* and *A*-*C_i_* + CF curves, normalized temperature response functions with a reference temperature of 25 °C are used for fitting $V_{cmax}$, $R_{L}$, $J$, and $T_{p}$. In contrast, $\alpha_{old}$, $\alpha_{G}$, $\alpha_{S}$, $\alpha_{T}$, and $\tau$ are taken to be independent of temperature. Preset options are available for using the temperature response functions defined in Bernacchi et al. (2001) and Bernacchi, Pimentel, and Long (2003) (*c3_temperature_param_bernacchi*) and Sharkey et al. (2007) (*c3_temperature_param_sharkey*). These presets also calculate temperature-dependent values of other parameters that cannot be fit, such as $K_{c}$ and $K_{o}$.

For C_4_ *A*-*C_i_* curves, normalized temperature response functions with a reference temperature of 25 °C are used for fitting $V_{cmax}$, $V_{pmax}$, $R_{L}$, and $J$. A preset option is available for using the temperature response functions defined in von Caemmerer (2021) (*c4_temperature_param_vc*).

# S8. Likelihood and Confidence Intervals in *PhotoGEA*

Although more general mathematical definitions exist (Rossi 2018), in *PhotoGEA* the likelihood $\mathcal{L}$ that a set of $N$ observed $\left( A_{n},C_{i} \right)$ pairs were sampled from a model with $M$ parameters $\boldsymbol{P}=(P_{1},P_{2},\ldots,P_{M})$ is defined to be

$\mathcal{L}\left( \boldsymbol{P},\sigma\right)=\prod_{k=1}^{N} p\left( A_{n}^{k} | A_{mod}\left( C_{i}^{k},\boldsymbol{P} \right),\sigma\right)$, (**E1**)

where $A_{n}^{k}$ and $C_{i}^{k}$ are the $k^{th}$ observed values of $A_{n}$ and $C_{i}$, $A_{mod}\left( C_{i}^{k},\boldsymbol{P} \right)$ is the corresponding modeled value of $A_{n}$, and $p\left( A_{n}^{k} | A_{mod}\left( C_{i}^{k},\boldsymbol{P} \right),\sigma\right)$ is the probability of observing $A_{n}^{k}$ given a Gaussian probability distribution with mean $A_{mod}\left( C_{i}^{k},\boldsymbol{P} \right)$ and standard deviation $\sigma$ (assumed to be the same for all points in the curve). The values of $p$ in Equation **3** can be rewritten using the definition of a Gaussian probability distribution: $p\left( x|\mu,\sigma\right)=\frac{1}{\sqrt{2\pi}\sigma}\exp\left[ -\left( \frac{x-\mu}{\sqrt{2}\sigma} \right)^{2} \right]$, where $\text{exp}$ refers to exponentiation with base $e$. This leads to another expression for the likelihood:

$\mathcal{L}\left( \boldsymbol{P},\sigma\right)=\left( \frac{1}{\sqrt{2\pi}\sigma} \right)^{N}\cdot exp \left[ -\frac{1}{{2\sigma}^{2}}\sum_{k=1}^{N} \left( A_{n}^{k}-A_{mod}\left( C_{i}^{k},\boldsymbol{P} \right) \right)^{2} \right]$. (**E2**)

The term $\sum_{k=1}^{N} \left( A_{n}^{k}-A_{mod}\left( C_{i}^{k},\boldsymbol{P} \right) \right)^{2}$ can be recognized as the sum of squared residuals (SSR). Because of this, the likelihood (for any value of $\sigma$) is maximized when the SSR is minimized. Thus, the best-fit parameter estimates from maximum likelihood regression with equal variance do not depend on $\sigma$ and always agree with the best-fit parameters from least squares regression (Banks and Joyner 2017). In practice, *PhotoGEA* performs maximum likelihood regression by minimizing the negative logarithm of $\mathcal{L}$ because most optimization algorithms are designed to minimize rather than maximize.

Once the best-fit parameters ($\boldsymbol{P}_{best}$) have been found using an optimization algorithm, the RMSE can be calculated (Equation **1** in the main text) and used as an estimate of the standard deviation ($\sigma_{RMSE}$), enabling the calculation of $\mathcal{L}_{max}\mathcal{=L}\left( \boldsymbol{P}_{best},\sigma_{RMSE} \right)$ (Banks and Joyner 2017). The region of parameter space where the likelihood ratio $\mathcal{L}/\mathcal{L}_{max}$ exceeds a threshold value $t$ can be thought of as a “confidence region” where the likelihood is sufficiently high (Rossi 2018), and its boundaries can be used to calculate “likelihood ratio confidence intervals” (Doganaksoy 2021). Such confidence intervals are one type of non-Gaussian confidence interval, where other methods include bootstrapping and transformation (Pek, Wong, and Wong 2017).

The confidence region may have a complex shape. In *PhotoGEA*, confidence intervals are calculated by finding the boundaries of this region along one-dimensional lines passing through the best-fit value. In other words, the confidence interval for parameter $P_{i}\in\boldsymbol{P}$ is determined by the continuous range of $\delta$ that includes 0 and satisfies

$\mathcal{L}\left( \boldsymbol{P}_{best}\boldsymbol{+}{\delta\boldsymbol{I}}_{i},\sigma_{RMSE} \right)\geq t\cdot\mathcal{L}_{max}$, (**E3**)

where $\boldsymbol{I}_{i}$ is the unit vector along the $P_{i}$ direction in parameter space. The largest (most positive) value of $\delta$ satisfying **E3** ($\delta^{+}$) defines the upper limit of the interval, while the smallest (most negative) value ($\delta^{-}$) defines the lower limit. In practice, because *PhotoGEA* calculates the logarithm of $\mathcal{L}$, confidence intervals are actually calculated by finding the largest and smallest $\delta$ such that

$\ln\mathcal{L}\left( \boldsymbol{P}_{best}\boldsymbol{+}{\delta\boldsymbol{I}}_{i},\sigma_{RMSE} \right)-\ln t-\ln\mathcal{L}_{max}\geq0$, (**E4**)

where $\text{ln}$ refers to a logarithm with base $e$. This is done numerically in *R* using the *stats::uniroot* function. The actual confidence interval then ranges from $P_{i,best}-\delta^{-}$ to $P_{i,best}+\delta^{+}$, where $P_{i,best}$ is the best-fit value of $P_{i}$. This method does not require that the likelihood function is continuous.

An appropriate value of the threshold $t$ can be found by considering the probability that $\mathcal{L}\left( \boldsymbol{P},\sigma_{RMSE} \right)/\mathcal{L}_{max}$ exceeds $t$:

$\begin{matrix} p\left( \frac{\mathcal{L}\left( \boldsymbol{P},\sigma_{RMSE} \right)}{\mathcal{L}_{max}}\geq t \right) & = & p\left( \ln\left[ \frac{\mathcal{L}\left( \boldsymbol{P},\sigma_{RMSE} \right)}{\mathcal{L}_{max}} \right]\geq\ln t \right) \\ & = & p\left( \ln\mathcal{L}\left( \boldsymbol{P},\sigma_{RMSE} \right)-\ln\mathcal{L}_{max}\geq\ln t \right) \\ & = & p\left( -2\left[ \ln\mathcal{L}\left( \boldsymbol{P},\sigma_{RMSE} \right)-\ln\mathcal{L}_{max} \right]\leq-2\ln t \right) \end{matrix}$ (**E5**)

In Equation (**E5**), the expression $-2\left[ \ln\mathcal{L}\left( \boldsymbol{P},\sigma_{RMSE} \right)-\ln\mathcal{L}_{max} \right]$ can be recognized as the likelihood-ratio test statistic, where the null hypothesis is that $\mathcal{L}\left( \boldsymbol{P},\sigma_{RMSE} \right)$ and $\mathcal{L}_{max}$ are not significantly different (Rossi 2018). Thus, by Wilks’ theorem (Wilks 1938), $-2\left[ \ln\mathcal{L}\left( \boldsymbol{P},\sigma_{RMSE} \right)-\ln\mathcal{L}_{max} \right]$ approaches the chi-squared distribution with one degree of freedom when the null hypothesis is true and $N$ is sufficiently large. In that case Equation (**E5**) can be re-expressed as

$p\left( \frac{\mathcal{L}\left( \boldsymbol{P},\sigma_{RMSE} \right)}{\mathcal{L}_{max}}\geq t \right)\approx p\left( \chi_{1}^{2}\leq-2\ln t \right)$ (**E6**)

From Equation (**E6**), $p$ > 0.05 for $\chi_{1}^{2}$ below 3.835, corresponding to $t=e^{-3.835/2}$ = 0.147 (Rossi 2018). Thus, in most cases, confidence intervals calculated using a likelihood ratio threshold of 14.7% closely approximate standard 95% confidence intervals.

Note that by calculating confidence intervals using one-dimensional slices through parameter space, *PhotoGEA* does not consider covariation between parameters. Nevertheless, these intervals contain the true parameter value for more than 95% of estimates when fitting simulated *C_3_* *A*-*C_i_* curves, and intervals that extend to infinity can be used to identify unreliable parameter estimates, enabling *PhotoGEA* to avoid false positives (Supplemental Section **S10**). Quantitative uncertainties calculated by other fitting tools also do not fully consider covariation. For example, in the *plantecophys* fitting tools, Equation **A11** is fit to points where assimilation is identified as being Rubisco-limited; this fit returns standard errors for $V_{cmax}$ and $R_{L}$, including covariation between these two parameters, but the standard error calculation cannot consider covariation of either parameter with $J$ or $T_{p}$, which are estimated from separate fits to other parts of the curve.

# S9. Detailed Fitting Settings for Each Tool

## S9.1 C_3_ *A*-*C_i_* curves

Tobacco *A*-*C_i_* curves were fit on a $C_{i}$ basis (in other words, with infinite mesophyll conductance and $C_{c}=C_{i}$) using six methods, as described in detail below. All fits used temperature response parameters from Sharkey et al. (2007) to calculate leaf-temperature-dependent values of $K_{c}$, $K_{o}$, and $\Gamma^{*}$. For tools that calculate $J_{max}$ (*plantecophys* and *photosynthesis*), the initial slope and curvature of the response of $J$ to $Q_{in}$ were set to $\alpha$ = 0.85 ∙ (1 - 0.15) / 2 and $\theta$ = 0.7, respectively (von Caemmerer 2000). For each fit, residuals and summary statistics (including the RMSE and AIC) were calculated using the *residual_stats* function from *PhotoGEA*.

- The *fit_c3_aci* function from *PhotoGEA*, allowing $R_{L}$, $V_{cmax}$, $J$, $T_{p}$, and $\alpha_{old}$ to vary.
  - The *DEoptim* optimizer was used with 200 generations (the default).
- The *fit_c3_aci* function from *PhotoGEA*, allowing $R_{L}$, $V_{cmax}$, $J$, $T_{p}$, $\alpha_{G}$, and $\alpha_{S}$ to vary.
  - The *DEoptim* optimizer was used with 200 generations (the default).
- The “TPU II” tab of version 2.0 of the *PCE Calculator* Excel spreadsheet (Sharkey 2016), allowing $R_{L}$, $V_{cmax}$, $J$, $T_{p}$, and $\alpha_{old}$ to vary.
  - Mesophyll conductance at leaf temperature was fixed to 1,000,000 μmol m^-2^ s^-1^ Pa^-1^ (effectively infinite).
  - Although this tool is intended for fitting $\alpha_{old}$, cells *J11:J31* (used to calculate the TPU-limited assimilation rate) do not correctly implement Equation **A13**. This mistake in the tool was corrected before fitting the curves.
  - The temperature response parameters for $T_{p}$ in cells *E73:H73* were changed to the values specified in Sharkey et al. (2007); the other parameters did not require any changes.
  - For each curve, limiting processes for each point were initially based on the measured values of $A_{n}$ and $\phi_{PSII}$ (Supplemental Figure **S5**). Regions where $\phi_{PSII}$ or $A_{n}$ decreases with $C_{i}$ were assigned to TPU limitations, regions where $\phi_{PSII}$ is independent of $C_{i}$ were assigned to RuBP regeneration limitations, and regions where $\phi_{PSII}$ increases with $C_{i}$ were assigned to Rubisco limitations. Following an initial fit, the assignments were adjusted as necessary to minimize the sum of squares. Any fit where the actual limiting state differed from the assigned limiting state was rejected as being inadmissible. For three curves, it was necessary to exclude 1-3 points to achieve a good admissible fit.
  - Individual fits were generally not retained when using *PCE calculator*, following common practice across the literature; the one available fit is shown in Figure **1** of the main text.
- The *fitaci* function from version 1.4-6 of the *plantecophys* *R* package (Duursma 2015), allowing $R_{L}$, $V_{cmax}$, $J$, and $T_{p}$ to vary.
  - Although this function provides both full-curve and exhaustive fitting methods, TPU is only included with the exhaustive method (referred to as “bilinear,” in contrast to the “default” option), so that method is used here.
  - This function directly provides values of $J_{max}$ rather than $J$, but it is possible to retrieve values of $J$ using the non-exported *Jfun* function from the *plantecophys* package.
  - The values of $A_{c}$, $A_{j}$, and $A_{p}$ directly returned by this function do not include losses from non-photorespiratory CO_2_ release, but the value of $R_{L}$ can be subtracted from these rates so they agree with Equations **A11-13** above and can be compared to the outputs from the other packages.
  - If no points are found to be limited by TPU, this function returns a $T_{p}$ value of 1000 μmol m^-2^ s^-1^; we exclude these values when analyzing results because they are not meaningful.
  - If no points are found to be limited by RuBP regeneration, this function returns a $J_{max}$ value of 1,000,000 μmol m^-2^ s^-1^ (at leaf temperature); we exclude these values when analyzing results because they are not meaningful.
  - This function is able to (optionally) rescale $V_{cmax}$ and $J_{max}$ to their values at 25 °C, but values of $T_{p}$ and $R_{L}$ are always reported at leaf temperature. For the fits presented in the main manuscript, values were reported at leaf temperature.
  - The default Arrhenius activation energies for $V_{cmax}$ and $J_{max}$ were replaced with values from Sharkey et al. (2007) when calling the function. Values were supplied in units of J mol^-1^ K^-1^, as required by *fitaci*.
  - The default values for $\Gamma^{*}$ and the Michaelis-Menten constant for Rubisco carboxylation ($K_{M}$, defined as $K_{c}\cdot\left( 1+O/K_{o} \right)$) were replaced with their leaf-temperature-dependent values, averaged across all points in the curve. Values were supplied in units of μbar, as required by *fitaci*.
  - The default values of $\alpha$ and $\theta$ (related to $J$ and $J_{max}$) were replaced by the values discussed above (0.36125 and 0.7, respectively).
  - A constant value of $\alpha_{old}=0$ was used for all fits. While it is possible to specify other values, it is not practical to manually change this value for each curve.
  - Data frames were subsetted to just the columns required by the function ($A_{n}$, $C_{i}$, $T_{leaf}$, and $Q_{in}$), and column names were supplied when calling *fitaci*.
- The *fit_aci_response* function from version 2.1.4 of the *photosynthesis* *R* package (Stinziano et al. 2021), allowing $R_{L}$, $V_{cmax}$, $J$, and $T_{p}$ to vary.
  - $R_{L}$ as returned from this tool is the negative of $R_{L}$ as defined in Supplementary Section **S1**, but can be multiplied by negative one so it can be compared to the outputs from the other packages.
  - If no points are found to be limited by TPU, this function returns a value of 1000 μmol m^-2^ s^-1^; we exclude these values when analyzing results because they are not meaningful.
  - The default Arrhenius activation energies and values at 25 °C for $K_{c}$, $K_{o}$, and $\Gamma^{*}$ were replaced by the values from Sharkey et al. (2007) when calling the function. Activation energies were supplied in units of J mol^-1^ K^-1^, as required by *fit_aci_response*; values of for $K_{c}$, $K_{o}$, and $\Gamma^{*}$ at 25 °C were supplied in units of μbar, kPa, and μmol mol^-1^, respectively, as required by *fit_aci_response*.
  - The default values of $\alpha$ and $\theta$ (related to $J$ and $J_{max}$) were replaced by the values discussed above (0.36125 and 0.7, respectively).
  - This function is not able to rescale any output parameters to 25 °C; instead, they are all reported at leaf temperature.
  - A constant value of $\alpha_{old}=0$ was used for all fits. While it is possible to specify other values, it is not practical to manually change this value for each curve.
  - Data frames were subsetted to just the columns required by the function ($A_{n}$, $C_{i}$, $T_{leaf}$, and $Q_{in}$), and column names were supplied when calling *fit_aci_response*.
- The *fitComplete* function from version 1.2.0 of the *msuRACiFit* *R* package (Gregory et al. 2021) , allowing $R_{L}$, $V_{cmax}$, $J$, $T_{p}$, $\alpha_{G}$, and $\alpha_{S}$ to vary.
  - The default values of $K_{c}$, $K_{o}$, and $\Gamma^{*}$ were replaced with their leaf-temperature-dependent values, averaged across all points in the curve. Values were supplied in units of Pa, kPa, and Pa, respectively, as required by *fitComplete*.
  - This function is not able to rescale any output parameters to 25 °C; instead, they are all reported at leaf temperature.
  - Data frames were subsetted to just the columns required by the function ($A_{n}$, and $P_{C_{i}}$), and column names were supplied when calling *fitComplete*.

## S9.2 C_3_ *A*-*C_i_* + CF curves

Soybean *A*-*C_i_* + CF curves were fit using the *fit_c3_variable_j* function from *PhotoGEA*, allowing $R_{L}$, $V_{cmax}$, $J$, $T_{p}$, $\alpha_{old}$, and $\tau$ to vary. The *DEoptim* optimizer was used with 800 generations, and the lower bound for $R_{L}$ was set to -1 μmol m^-2^ s^-1^ to prevent a bad fit for one curve. These fits used temperature response parameters from Sharkey et al. (2007), with the exception of $K_{c}$, $K_{o}$, and $\Gamma^{*}$, where the Arrhenius parameters were estimated from soybean Rubisco $K_{c}$, $K_{o}$, and specificity ($S_{C/O}$) values reported in Orr et al. (2016):

- $c$ = 42.382, $E_{a}$ = 90.476 kJ mol^-1^ for $K_{c}$ expressed in μmol mol^-1^
- $c$ = 12.784, $E_{a}$ = 16.565 kJ mol^-1^ for $K_{o}$ expressed in mmol mol^-1^
- $c$ = 14.127, $E_{a}$ = 26.004 kJ mol^-1^ for $\Gamma^{*}$ expressed in μmol mol^-1^

## S9.3 C_4_ *A*-*C_i_* curves

Maize and sorghum *A*-*C_i_* curves were fit using the *PhotoGEA* *R* package with three different approaches:

- The empirical approach, where points with $C_{i}$ ≤ 60 μmol mol^-1^ are fit using the *fit_c4_aci* function (fixing $g_{bs}$ to zero, setting $g_{mc}$ to infinity, and allowing $R_{L}$ and $V_{pmax}$ to vary) and the entire curve is fit using the *fit_c4_aci_hyperbola* function (allowing $R_{L}$, $V_{max}$, $\theta_{H}$, and $m$ to vary).
- The mechanistic approach assuming Rubisco limitations, where the entire curve is fit using the *fit_c4_aci* function (allowing $R_{L}$, $V_{pmax}$, and $V_{cmax}$ to vary).
- The mechanistic approach assuming light limitations, where the entire curve is fit using the *fit_c4_aci* function (allowing $R_{L}$, $V_{pmax}$, and $J_{max}$ to vary).

For each approach, the *DEoptim* optimizer was used with 200 generations (the default). Temperature response parameters from von Caemmerer (2021) were used for the mechanistic C_4_ fits. When assuming Rubisco limitations, $J_{max}$ and $V_{pr}$ are both fixed to 1000 μmol m^-2^ s^-1^ so that light and PEP regeneration are never able to limit $A_{n}$; likewise, when assuming light limitations, $V_{cmax}$ and $V_{pr}$ are both fixed to 1000 μmol m^-2^ s^-1^.

# S10. Fitting Simulated C_3_ *A*-*C_i_* Curves With Each *R* Package

One way to test the performance of each fitting tool is to estimate parameter values from simulated *A*-*C_i_* curves, where the true parameter values are known beforehand. Here we perform such a test using the *PhotoGEA*, *plantecophys*, *photosynthesis*, and *msuRACiFit* *R* packages to fit a large number of simulated *A*-*C_i_* curves with a range of parameter values. Following the method of (Gu et al. 2010), curves were simulated without any additional noise.

First, a sequence of seventeen $C_{c}$ values was chosen by calculating the average $C_{i}$ value at each reference CO_2_ set-point across the set of measured tobacco response curves: 25, 33, 41, 47, 53, 68, 81, 107, 132, 182, 240, 388, 473, 659, 853, 1044, and 1329 μmol mol^-1^. Then, random combinations of $V_{cmax}$, $J$, $T_{p}$, $R_{L}$, $\alpha_{old}$, $\alpha_{G}$, and $\alpha_{S}$ were chosen using Latin hypercube sampling. For each parameter set, $A_{n}$ was calculated at each value of $C_{c}$ to generate a response curve. For each curve, the true parameter values were retained, along with the number of points where $A_{n}$ is equal to $A_{c}$, $A_{j}$, or $A_{p}$ (denoted as $N_{A_{c}}$, $N_{A_{j}}$, and $N_{A_{p}}$, respectively).

Three categories of curves were generated and separately analyzed: no reverse sensitivity ($\alpha_{old}=\alpha_{G}=\alpha_{S}=0$), “old” reverse sensitivity ($\alpha_{G}=\alpha_{S}=0$), and “new” reverse sensitivity ($\alpha_{old}=0$). 200 curves were generated for each category. The sampled range for each parameter was:

- $V_{cmax}$: 20 – 120 μmol m^-2^ s^-1^
- $J$: 20 – 200 μmol m^-2^ s^-1^
- $T_{p}$: 2 – 18 μmol m^-2^ s^-1^
- $R_{L}$: 0.5 – 2.5 μmol m^-2^ s^-1^
- $\alpha_{old}$: 0 – 1 when not fixed to 0
- $\alpha_{G}$: 0 – 1 when not fixed to 0
- $\alpha_{S}$: 0 – 1 when not fixed to 0

Finally, each of the resulting 600 curves was fit with each of the packages mentioned above. The results for curves without any reverse sensitivity are discussed in detail in Section **S10.1**, while the results for the other two categories are summarized in Sections **S10.2** and **S10.3**.

## S10.1 Curves Without Reverse Sensitivity

One way to compare the results from each package is to determine the rate of categorical errors (false positives and negatives). A “false positive” is said to occur when a tool provides an estimated value for a parameter when the corresponding process is not evident in the curve itself; for example, a value of $T_{p}$ when no points in the curve are limited by TPU ($N_{A_{p}}=0$). Likewise, a “false negative” is said to occur when a tool does not provide an estimated value for a parameter when the corresponding process is evident in the curve; for example, no value of $T_{p}$ when one or more points in the curve are limited by TPU ($N_{A_{p}}>0$).


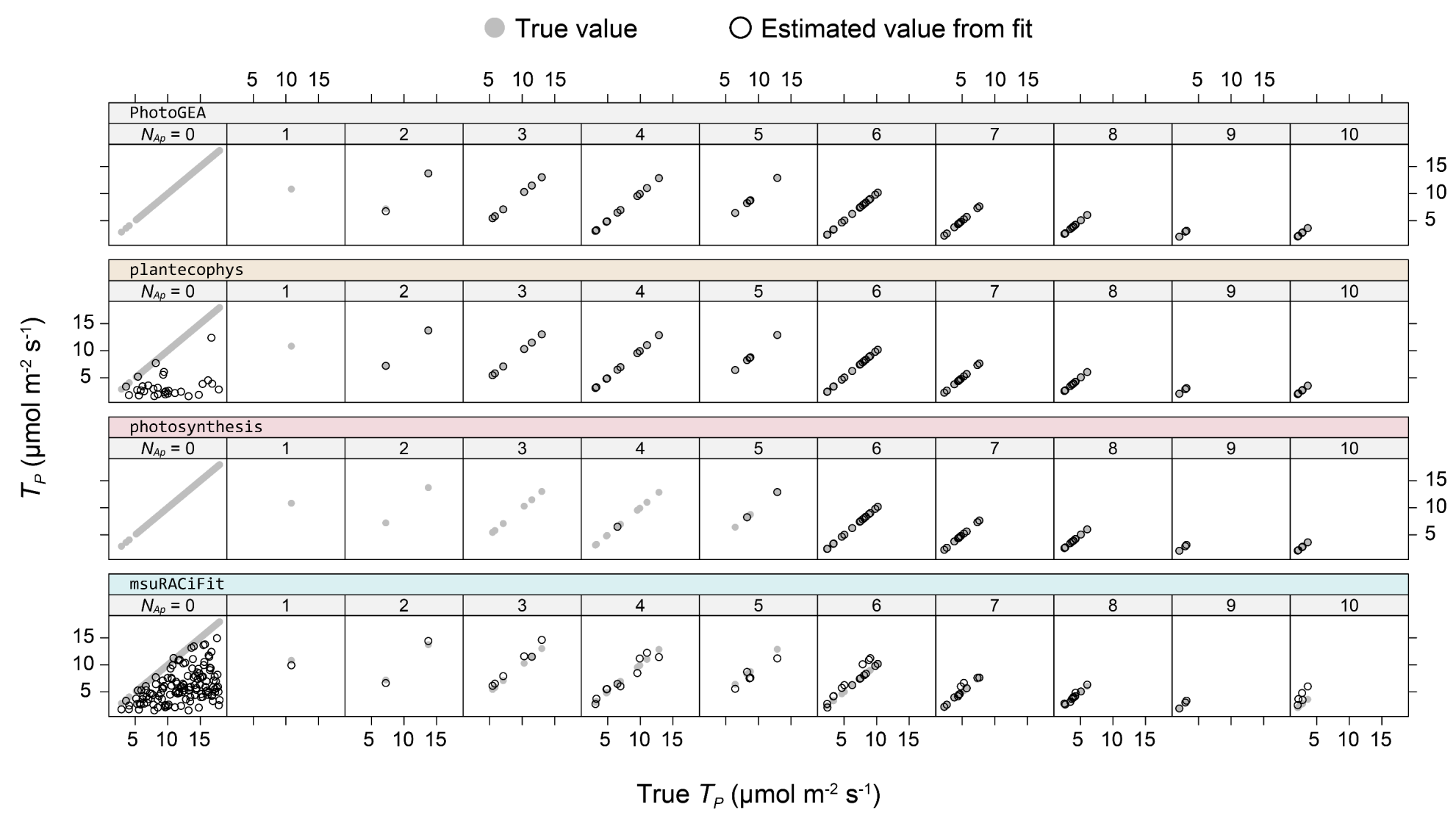


Figure S19: True (filled gray circles) and estimated (open black circles) values of $T_{p}$ as determined from simulated C_3_ A-C_i_ curves (without any reverse sensitivity to CO_2_) by fitting tools from the PhotoGEA, plantecophys, photosynthesis, and msuRACiFit packages. Parameter values are grouped by package and by the number of points in each curve where $A_{n}=A_{p}$ ($N_{A_{p}}$). For the PhotoGEA fits, $\alpha_{old}$ was allowed to vary.

Among the 200 simulated curves without any reverse sensitivity, 130 happen to have no TPU limitations at all ($N_{A_{p}}=0$), while the remaining 70 curves have at least one TPU-limited point. Across the packages and curves, both false positive and false negative estimates for $T_{p}$ were found to occur (Figure **S19**). False positives can be seen when there are estimated values (open black circles) for curves without TPU limitations (columns where $N_{A_{p}}=0$ in Figure **S19**). False negatives can be seen when there are fewer estimates (open black circles) than curves (solid gray circles) when TPU limitations are present (columns where $N_{A_{p}}>0$ in Figure **S19**). The *PhotoGEA*, *plantecophys*, *photosynthesis*, and *msuRACiFit* packages returned 0, 30, 0, and 130 false positives, respectively, corresponding to categorical error rates of 0%, 23.1%, 0%, and 100% (Table **S3**). Likewise, among the 70 curves with TPU limitations, the packages each returned 1, 1, 21, and 0 false negatives, corresponding to categorical error rates of 1.4%, 1.4%, 30%, and 0%, respectively. From this we can see that for the particular case of estimating $T_{p}$ from curves without any reverse sensitivity to CO_2_, *PhotoGEA* has a low incidence of both types of categorical error. The *plantecophys* tool has a high rate of false positives, while *photosynthesis* has a high rate of false negatives, which tend to occur for curves four or fewer TPU-limited points (Figure **S19**). The *msuRACiFit* tool always returns an estimate for $T_{p}$, leading to 0% false negatives but 100% false positives.

A similar analysis for $J$ estimates (Table **S3**) shows that no tools returned any false negatives, but the incidence of false positives varied greatly, with *PhotoGEA* having the lowest incidence (3.5%), followed by *plantecophys* (80%). Both *photosynthesis* and *msuRACiFit* returned $J$ estimates for all curves where $N_{A_{j}}=0$, an error rate of 100%. There were also major differences in categorical errors for $V_{cmax}$ estimates (Table **S3**). Again, no tool returned any false negatives. *PhotoGEA* did not return any false positives, but *plantecophys*, *photosynthesis*, and *msuRACiFit* returned $V_{cmax}$ estimates for all curves where $N_{A_{c}}=0$, an error rate of 100%. Thus, across $T_{p}$, $J$, and $V_{cmax}$, *PhotoGEA* is by far the least likely tool to make categorical errors, either false positives or false negatives.

Another way to characterize the performance of each tool is to check whether the reported confidence interval for each parameter actually contains the true value. Across all parameters and curve types, *PhotoGEA* has the highest rate of “correct confidence intervals” (C-CI), often achieving a 100% success rate (Table **S3**). Its worst performance is for $J$ estimates from curves that do not exhibit any RuBP-regeneration-limited points, but its confidence intervals are still correct for 96.5% of curves in this group. Since all *PhotoGEA* confidence intervals extend to infinity for these curves, this means that the $J$ lower bound estimates are below the true value for 96.5% of curves. Yet, in this same category, the other packages each have a 0% success rate.

Confidence intervals estimated by *plantecophys* are highly successful for estimates of $R_{L}$, and for estimates of $V_{cmax}$ from curves that exhibit Rubisco limitations. Intervals estimated by *photosynthesis* are highly successful for estimates of $R_{L}$, for estimates of $V_{cmax}$ from curves that exhibit Rubisco limitations, and for estimates of $J$ from curves that exhibit RuBP regeneration limitations. Note that *msuRACiFit* does not return standard errors or confidence intervals. Thus, although other tools produce reliable confidence intervals for some parameters, none are as reliable as *PhotoGEA*.

Finally, it is possible to quantify the accuracy of the parameter estimates from each package using the root mean square error, defined by $\sqrt{\sum\left( predicted-true \right)^{2}/n}$, where the sum runs over all $n$ parameter estimates in a particular category of curves. For example, among curves with $N_{A_{j}}>0$, *PhotoGEA* returned 115 estimates of $J$, with an RMSE of 0.09216 μmol m^-2^ s^-1^ (Table **S3**). This is an order magnitude lower than the RMSE of $J$ estimates from the other tools for this type of curve – the *plantecophys*, *photosynthesis*, and *msuRACiFit* RMSE values are much higher at 2.69347, 2.90720, and 5.83665 μmol m^-2^ s^-1^, respectively – indicating that the $J$ estimates from *PhotoGEA* are closest to the true values. Note that it is not sensible to calculate RMSE values for $J$ estimates from curves where $N_{A_{j}}=0$ because any such estimates are false positives. Estimates of $V_{cmax}$ and $R_{L}$ from *PhotoGEA* also show RMSE values at least an order of magnitude lower than those from other packages (Table **S3**). For $T_{p}$, the *plantecophys* and *photosynthesis* packages outperform *PhotoGEA*, but the RMSE values are within a factor of two, indicating a similar level of accuracy (Table **S3**).

To summarize the results from this set of simulated curves, the estimates from *PhotoGEA* are least likely to include categorical errors (false positives or false negatives), the confidence intervals from *PhotoGEA* are most likely to include the true parameter value, and the estimates from *PhotoGEA* are generally closest to the true values (with the exception of $T_{p}$, where the PhotoGEA estimates are nearly as good as the best tools).

| **Parameter** | **Curve type** | ***N*** | **Package** | ***N_est_*** | **Error rate (%)** | **N_C-CI_** | **C-CI rate (%)** | **RMSE**  **(μmol m^-2^ s^-1^)** |
| --- | --- | --- | --- | --- | --- | --- | --- | --- |
| $J$ | $A_{j}$ limited | 115 | *PhotoGEA* | 115 | 0.0 | 115 | 100.0 | 0.09216 |
|  |  |  | *plantecophys* | 115 | 0.0 | 0 | 0.0 | 2.69347 |
|  |  |  | *photosynthesis* | 115 | 0.0 | 107 | 93.0 | 2.90720 |
|  |  |  | *msuRACiFit* | 115 | 0.0 | 0 | 0.0 | 5.83665 |
|  | Not $A_{j}$ limited | 85 | *PhotoGEA* | 3 | 3.5 | 82 | 96.5 | – |
|  |  |  | *plantecophys* | 68 | 80.0 | 0 | 0.0 | – |
|  |  |  | *photosynthesis* | 85 | 100.0 | 0 | 0.0 | – |
|  |  |  | *msuRACiFit* | 85 | 100.0 | 0 | 0.0 | – |
| $T_{p}$ | $A_{p}$ limited | 70 | *PhotoGEA* | 69 | 1.4 | 69 | 98.6 | 0.05541 |
|  |  |  | *plantecophys* | 69 | 1.4 | 0 | 0.0 | 0.02989 |
|  |  |  | *photosynthesis* | 49 | 30.0 | 45 | 64.3 | 0.02019 |
|  |  |  | *msuRACiFit* | 70 | 0.0 | 0 | 0.0 | 0.90974 |
|  | Not $A_{p}$ limited | 130 | *PhotoGEA* | 0 | 0.0 | 130 | 100.0 | – |
|  |  |  | *plantecophys* | 30 | 23.1 | 0 | 0.0 | – |
|  |  |  | *photosynthesis* | 0 | 0.0 | 0 | 0.0 | – |
|  |  |  | *msuRACiFit* | 130 | 100.0 | 0 | 0.0 | – |
| $V_{cmax}$ | $A_{c}$ limited | 174 | *PhotoGEA* | 174 | 0.0 | 174 | 100.0 | 0.09389 |
|  |  |  | *plantecophys* | 174 | 0.0 | 162 | 93.1 | 4.53374 |
|  |  |  | *photosynthesis* | 174 | 0.0 | 164 | 94.3 | 2.56770 |
|  |  |  | *msuRACiFit* | 174 | 0.0 | 0 | 0.0 | 4.61974 |
|  | Not $A_{c}$ limited | 26 | *PhotoGEA* | 0 | 0.0 | 26 | 100.0 | – |
|  |  |  | *plantecophys* | 26 | 100.0 | 1 | 3.8 | – |
|  |  |  | *photosynthesis* | 26 | 100.0 | 1 | 3.8 | – |
|  |  |  | *msuRACiFit* | 26 | 100.0 | 0 | 0.0 | – |
| $R_{L}$ | all | 200 | *PhotoGEA* | 200 | 0.0 | 200 | 100.0 | 0.00172 |
|  |  |  | *plantecophys* | 200 | 0.0 | 195 | 97.5 | 0.05323 |
|  |  |  | *photosynthesis* | 200 | 0.0 | 200 | 100.0 | 0.03459 |
|  |  |  | *msuRACiFit* | 200 | 0.0 | 0 | 0.0 | 1.54598 |

Table S3: Summary of fit results from a set of 200 simulated C_3_ A-C_i_ curves (without any reverse sensitivity to CO_2_). For the PhotoGEA fits, $\alpha_{old}$ was allowed to vary. In the “curve type” column, “$A_{j}$ limited” means that at least one point in the curve has $A_{n}=A_{j}$ ($N_{A_{j}}>0$), while “not $A_{j}$ limited” means that $N_{A_{j}}=0$; likewise for the other limiting processes. N_est_: the number of parameter estimates from each package for a particular parameter and curve type. Error rate: the rate of categorical errors (false positives when the process is not limiting, false negatives otherwise). N_C-CI_: the number of parameter estimates with “correct confidence intervals” (C-CI); in other words, where the confidence interval includes the true value (within a margin of 1% to account for roundoff errors). C-CI rate: the fraction of estimates with correct confidence intervals. RMSE: the root mean squared error for the parameter estimates, calculated as $\sqrt{\sum\left( estimated-true \right)^{2}/N_{est}}$. Note: $R_{L}$ always influences $A_{n}$, so it can always be estimated regardless of which limiting processes are present, and only false negative categorical errors are possible for $R_{L}$.

## S10.2 Curves With Reverse Sensitivity (α_old_)

The results from simulated curves that potentially exhibit reverse sensitivity to CO_2_ due to randomly chosen values of $\alpha_{old}$ are similar to those from curves without reverse sensitivity (Table **S4**). Again, *PhotoGEA* is least likely to produce categorical errors, and in fact, did not report any false negatives or false positives at all for this data set. Confidence intervals from *PhotoGEA* are also the most likely to contain the true value. Estimated values from *PhotoGEA* exhibit the lowest RMSE for all parameters, in contrast to the results in Section **S10.1**, where it was not the most precise for $T_{p}$ estimates.

| **Parameter** | **Curve type** | ***N*** | **Package** | ***N_est_*** | **Error rate (%)** | **N_C-CI_** | **C-CI rate (%)** | **RMSE**  **(μmol m^-2^ s^-1^)** |
| --- | --- | --- | --- | --- | --- | --- | --- | --- |
| $J$ | $A_{j}$ limited | 120 | *PhotoGEA* | 120 | 0.0 | 120 | 100.0 | 0.09671 |
|  |  |  | *plantecophys* | 120 | 0.0 | 0 | 0.0 | 0.82778 |
|  |  |  | *photosynthesis* | 120 | 0.0 | 114 | 95.0 | 1.54131 |
|  |  |  | *msuRACiFit* | 120 | 0.0 | 0 | 0.0 | 5.97323 |
|  | Not $A_{j}$ limited | 80 | *PhotoGEA* | 0 | 0.0 | 80 | 100.0 | – |
|  |  |  | *plantecophys* | 61 | 76.3 | 0 | 0.0 | – |
|  |  |  | *photosynthesis* | 80 | 100.0 | 0 | 0.0 | – |
|  |  |  | *msuRACiFit* | 80 | 100.0 | 0 | 0.0 | – |
| $T_{p}$ | $A_{p}$ limited | 68 | *PhotoGEA* | 68 | 0.0 | 65 | 95.6 | 0.11967 |
|  |  |  | *plantecophys* | 67 | 1.5 | 0 | 0.0 | 0.52818 |
|  |  |  | *photosynthesis* | 53 | 22.1 | 6 | 8.8 | 0.42609 |
|  |  |  | *msuRACiFit* | 68 | 0.0 | 0 | 0.0 | 0.90257 |
|  | Not $A_{p}$ limited | 132 | *PhotoGEA* | 0 | 0.0 | 132 | 100.0 | – |
|  |  |  | *plantecophys* | 30 | 22.7 | 0 | 0.0 | – |
|  |  |  | *photosynthesis* | 0 | 0.0 | 0 | 0.0 | – |
|  |  |  | *msuRACiFit* | 132 | 100.0 | 0 | 0.0 | – |
| $V_{cmax}$ | $A_{c}$ limited | 174 | *PhotoGEA* | 174 | 0.0 | 174 | 100.0 | 0.09403 |
|  |  |  | *plantecophys* | 174 | 0.0 | 168 | 96.6 | 0.15480 |
|  |  |  | *photosynthesis* | 174 | 0.0 | 166 | 95.4 | 1.42325 |
|  |  |  | *msuRACiFit* | 174 | 0.0 | 0 | 0.0 | 2.09398 |
|  | Not $A_{c}$ limited | 26 | *PhotoGEA* | 0 | 0.0 | 26 | 100.0 | – |
|  |  |  | *plantecophys* | 26 | 100.0 | 1 | 3.8 | – |
|  |  |  | *photosynthesis* | 26 | 100.0 | 1 | 3.8 | – |
|  |  |  | *msuRACiFit* | 26 | 100.0 | 0 | 0.0 | – |
| $R_{L}$ | all | 200 | *PhotoGEA* | 200 | 0.0 | 200 | 100.0 | 0.00173 |
|  |  |  | *plantecophys* | 200 | 0.0 | 194 | 97.0 | 0.00771 |
|  |  |  | *photosynthesis* | 200 | 0.0 | 200 | 100.0 | 0.04135 |
|  |  |  | *msuRACiFit* | 200 | 0.0 | 0 | 0.0 | 1.47386 |

Table S4: Summary of fit results from a set of 200 simulated C_3_ A-C_i_ curves (with reverse sensitivity to CO_2_ achieved via potentially nonzero $\alpha_{old}$). For the PhotoGEA fits, $\alpha_{old}$ was allowed to vary. In the “curve type” column, “$A_{j}$ limited” means that at least one point in the curve has $A_{n}=A_{j}$ ($N_{A_{j}}>0$), while “not $A_{j}$ limited” means that $N_{A_{j}}=0$; likewise for the other limiting processes. N_est_: the number of parameter estimates from each package for a particular parameter and curve type. Error rate: the rate of categorical errors (false positives when the process is not limiting, false negatives otherwise). N_C-CI_: the number of parameter estimates with “correct confidence intervals” (C-CI); in other words, where the confidence interval includes the true value (within a margin of 1% to account for roundoff errors). C-CI rate: the fraction of estimates with correct confidence intervals. RMSE: the root mean squared error for the parameter estimates, calculated as $\sqrt{\sum\left( estimated-true \right)^{2}/N_{est}}$. Note: $R_{L}$ always influences $A_{n}$, so it can always be estimated regardless of which limiting processes are present, and only false negative categorical errors are possible for $R_{L}$.

## S10.3 Curves with Reverse Sensitivity (α_G_ and α_S_)

The curves discussed in Sections **S10.1** and **S10.2** were simulated using the “original” FvCB model (Equations **A1**-**A5**), but the *msuRACiFit* package uses the “updated” version (Equations **A6**-**A10**). Because of this difference, *msuRACiFit* may be at a disadvantage when fitting those curves. To address this, curves were simulated with randomly-chosen $\alpha_{G}$ and $\alpha_{S}$ and tested as before. The results are summarized in Table **S5**.

These curves were more challenging to fit for all four packages. *PhotoGEA* had the lowest rates of categorical errors for several curve types, but it was the only package to return false negatives when estimating $V_{cmax}$ (5.1%), and was tied with *plantecophys* for the highest rate of false negatives when estimating $J$ (0.8%). The number of correct confidence intervals was generally smaller for this data set across all packages. Nevertheless, *PhotoGEA* was the most successful across most curve types, with one exception: $R_{L}$ confidence intervals, where 8.5% of *PhotoGEA* intervals were correct, compared to *photosynthesis* and *plantecophys*, which were each correct for 10% of curves. Although RMSE values were higher than for the curves in Sections **S10.1** and **S10.2**, *PhotoGEA* had the lowest value for each parameter, indicating that its estimates were still the most precise.

| **Parameter** | **Curve type** | ***N*** | **Package** | ***N_est_*** | **Error rate (%)** | **N_C-CI_** | **C-CI rate (%)** | **RMSE**  **(μmol m^-2^ s^-1^)** |
| --- | --- | --- | --- | --- | --- | --- | --- | --- |
| $J$ | $A_{j}$ limited | 133 | *PhotoGEA* | 132 | 0.8 | 58 | 43.6 | 2.37044 |
|  |  |  | *plantecophys* | 132 | 0.8 | 0 | 0.0 | 11.60597 |
|  |  |  | *photosynthesis* | 133 | 0.0 | 6 | 4.5 | 11.96985 |
|  |  |  | *msuRACiFit* | 133 | 0.0 | 0 | 0.0 | 7.72071 |
|  | Not $A_{j}$ limited | 67 | *PhotoGEA* | 0 | 0.0 | 63 | 94.0 | – |
|  |  |  | *plantecophys* | 52 | 77.6 | 0 | 0.0 | – |
|  |  |  | *photosynthesis* | 67 | 100.0 | 0 | 0.0 | – |
|  |  |  | *msuRACiFit* | 67 | 100.0 | 0 | 0.0 | – |
| $T_{p}$ | $A_{p}$ limited | 67 | *PhotoGEA* | 67 | 0.0 | 29 | 43.3 | 0.37097 |
|  |  |  | *plantecophys* | 67 | 0.0 | 0 | 0.0 | 0.51144 |
|  |  |  | *photosynthesis* | 53 | 20.9 | 20 | 29.9 | 0.41527 |
|  |  |  | *msuRACiFit* | 67 | 0.0 | 0 | 0.0 | 0.72911 |
|  | Not $A_{p}$ limited | 133 | *PhotoGEA* | 0 | 0.0 | 132 | 99.2 | – |
|  |  |  | *plantecophys* | 0 | 0.0 | 0 | 0.0 | – |
|  |  |  | *photosynthesis* | 21 | 15.8 | 0 | 0.0 | – |
|  |  |  | *msuRACiFit* | 133 | 100.0 | 0 | 0.0 | – |
| $V_{cmax}$ | $A_{c}$ limited | 156 | *PhotoGEA* | 148 | 5.1 | 96 | 61.5 | 0.86459 |
|  |  |  | *plantecophys* | 156 | 0.0 | 82 | 52.6 | 7.09267 |
|  |  |  | *photosynthesis* | 156 | 0.0 | 76 | 48.7 | 7.50199 |
|  |  |  | *msuRACiFit* | 156 | 0.0 | 0 | 0.0 | 1.49568 |
|  | Not $A_{c}$ limited | 44 | *PhotoGEA* | 0 | 0.0 | 43 | 97.7 | – |
|  |  |  | *plantecophys* | 44 | 100.0 | 0 | 0.0 | – |
|  |  |  | *photosynthesis* | 44 | 100.0 | 0 | 0.0 | – |
|  |  |  | *msuRACiFit* | 44 | 100.0 | 0 | 0.0 | – |
| $R_{L}$ | all | 200 | *PhotoGEA* | 200 | 0.0 | 17 | 8.5 | 0.85587 |
|  |  |  | *plantecophys* | 200 | 0.0 | 20 | 10.0 | 0.93245 |
|  |  |  | *photosynthesis* | 200 | 0.0 | 20 | 10.0 | 0.94701 |
|  |  |  | *msuRACiFit* | 200 | 0.0 | 0 | 0.0 | 1.01118 |

Table S5: Summary of fit results from a set of 200 simulated C_3_ A-C_i_ curves (with reverse sensitivity to CO_2_ achieved via potentially nonzero $\alpha_{G}$ and $\alpha_{S}$). For the PhotoGEA fits, $\alpha_{G}$ and $\alpha_{S}$ were allowed to vary. In the “curve type” column, “$A_{j}$ limited” means that at least one point in the curve has $A_{n}=A_{j}$ ($N_{A_{j}}>0$), while “not $A_{j}$ limited” means that $N_{A_{j}}=0$; likewise for the other limiting processes. N_est_: the number of parameter estimates from each package for a particular parameter and curve type. Error rate: the rate of categorical errors (false positives when the process is not limiting, false negatives otherwise). N_C-CI_: the number of parameter estimates with “correct confidence intervals” (C-CI); in other words, where the confidence interval includes the true value (within a margin of 1% to account for roundoff errors). C-CI rate: the fraction of estimates with correct confidence intervals. RMSE: the root mean squared error for the parameter estimates, calculated as $\sqrt{\sum\left( estimated-true \right)^{2}/N_{est}}$. Note: $R_{L}$ always influences $A_{n}$, so it can always be estimated regardless of which limiting processes are present, and only false negative categorical errors are possible for $R_{L}$.

# Works Cited

Banks, H. T., and Michele L. Joyner. 2017. “AIC under the Framework of Least Squares Estimation.” *Applied Mathematics Letters* 74 (December):33–45. https://doi.org/10.1016/j.aml.2017.05.005.

Bernacchi, C. J., C. Pimentel, and S. P. Long. 2003. “In Vivo Temperature Response Functions of Parameters Required to Model RuBP-Limited Photosynthesis.” *Plant, Cell & Environment* 26 (9): 1419–30. https://doi.org/10.1046/j.0016-8025.2003.01050.x.

Bernacchi, C. J., E. L. Singsaas, C. Pimentel, A. R. Portis Jr, and S. P. Long. 2001. “Improved Temperature Response Functions for Models of Rubisco-Limited Photosynthesis.” *Plant, Cell & Environment* 24 (2): 253–59. https://doi.org/10.1111/j.1365-3040.2001.00668.x.

Busch, Florian A. 2020. “Photorespiration in the Context of Rubisco Biochemistry, CO2 Diffusion and Metabolism.” *The Plant Journal* 101 (4): 919–39. https://doi.org/10.1111/tpj.14674.

Busch, Florian A., Rowan F. Sage, and Graham D. Farquhar. 2018. “Plants Increase CO2 Uptake by Assimilating Nitrogen via the Photorespiratory Pathway.” *Nature Plants* 4 (1): 46–54. https://doi.org/10.1038/s41477-017-0065-x.

Caemmerer, S. von. 2000. *Biochemical Models of Leaf Photosynthesis*. CSIRO Publishing. https://doi.org/10.1071/9780643103405.

———. 2021. “Updating the Steady-State Model of C4 Photosynthesis.” *Journal of Experimental Botany* 72 (17): 6003–17. https://doi.org/10.1093/jxb/erab266.

Doganaksoy, Necip. 2021. “A Simplified Formulation of Likelihood Ratio Confidence Intervals Using a Novel Property.” *Technometrics* 63 (1): 127–35. https://doi.org/10.1080/00401706.2020.1750488.

Duursma, Remko A. 2015. “Plantecophys - An R Package for Analysing and Modelling Leaf Gas Exchange Data.” *PLOS ONE* 10 (11): e0143346. https://doi.org/10.1371/journal.pone.0143346.

Farquhar, G. D., and S. von Caemmerer. 1982. “Modelling of Photosynthetic Response to Environmental Conditions.” In *Physiological Plant Ecology II: Water Relations and Carbon Assimilation*, edited by O. L. Lange, P. S. Nobel, C. B. Osmond, and H. Ziegler, 549–87. Encyclopedia of Plant Physiology. Berlin, Heidelberg: Springer. https://doi.org/10.1007/978-3-642-68150-9_17.

Farquhar, G. D., S. von Caemmerer, and J. A. Berry. 1980. “A Biochemical Model of Photosynthetic CO2 Assimilation in Leaves of C3 Species.” *Planta* 149 (1): 78–90. https://doi.org/10.1007/BF00386231.

Gregory, Luke M., Alan M. McClain, David M. Kramer, Jeremy D. Pardo, Kaila E. Smith, Oliver L. Tessmer, Berkley J. Walker, Leonardo G. Ziccardi, and Thomas D. Sharkey. 2021. “The Triose Phosphate Utilization Limitation of Photosynthetic Rate: Out of Global Models but Important for Leaf Models.” *Plant, Cell & Environment* 44 (10): 3223–26. https://doi.org/10.1111/pce.14153.

Gu, Lianhong, Stephen G. Pallardy, Kevin Tu, Beverly E. Law, and Stan D. Wullschleger. 2010. “Reliable Estimation of Biochemical Parameters from C3 Leaf Photosynthesis–Intercellular Carbon Dioxide Response Curves.” *Plant, Cell & Environment* 33 (11): 1852–74. https://doi.org/10.1111/j.1365-3040.2010.02192.x.

Johnson, Frank H., Henry Eyring, and R. W. Williams. 1942. “The Nature of Enzyme Inhibitions in Bacterial Luminescence: Sulfanilamide, Urethane, Temperature and Pressure.” *Journal of Cellular and Comparative Physiology* 20 (3): 247–68. https://doi.org/10.1002/jcp.1030200302.

Kirschbaum, M. U. F., and G. D. Farquhar. 1984. “Temperature Dependence of Whole-Leaf Photosynthesis in Eucalyptus Pauciflora Sieb. Ex Spreng.” *Australian Journal of Plant Physiology* 11 (6): 519–38. https://doi.org/10.1071/pp9840519.

Lochocki, Edward B., and Justin M. McGrath. 2025. “Widely Used Variants of the Farquhar-von-Caemmerer-Berry Model Can Cause Errors in Parameter Estimation.” bioRxiv. https://doi.org/10.1101/2025.03.11.642611.

Orr, Douglas J., André Alcântara, Maxim V. Kapralov, P. John Andralojc, Elizabete Carmo-Silva, and Martin A.J. Parry. 2016. “Surveying Rubisco Diversity and Temperature Response to Improve Crop Photosynthetic Efficiency.” *Plant Physiology* 172 (2): 707–17. https://doi.org/10.1104/pp.16.00750.

Pek, Jolynn, Augustine C. M. Wong, and Octavia C. Y. Wong. 2017. “Confidence Intervals for the Mean of Non-Normal Distribution: Transform or Not to Transform.” *Open Journal of Statistics* 7 (3): 405–21. https://doi.org/10.4236/ojs.2017.73029.

Rossi, Richard J. 2018. *Mathematical Statistics: An Introduction to Likelihood Based Inference*. John Wiley & Sons.

Sargent, Demi, Jeffrey S. Amthor, Joseph R. Stinziano, John R. Evans, Spencer M. Whitney, Michael P. Bange, David T. Tissue, Warren C. Conaty, and Robert E. Sharwood. 2024. “The Importance of Species-Specific and Temperature-Sensitive Parameterisation of A/Ci Models: A Case Study Using Cotton (Gossypium Hirsutum L.) and the Automated ‘OptiFitACi’ R-Package.” *Plant, Cell & Environment* 47 (5): 1701–15. https://doi.org/10.1111/pce.14800.

Sharkey, Thomas D. 1985. “Photosynthesis in Intact Leaves of C3 Plants: Physics, Physiology and Rate Limitations.” *The Botanical Review* 51 (1): 53–105. https://doi.org/10.1007/BF02861058.

———. 2016. “What Gas Exchange Data Can Tell Us about Photosynthesis.” *Plant, Cell & Environment* 39 (6): 1161–63. https://doi.org/10.1111/pce.12641.

Sharkey, Thomas D., Carl J. Bernacchi, Graham D. Farquhar, and Eric L. Singsaas. 2007. “Fitting Photosynthetic Carbon Dioxide Response Curves for C3 Leaves.” *Plant, Cell & Environment* 30 (9): 1035–40. https://doi.org/10.1111/j.1365-3040.2007.01710.x.

Stinziano, Jospeh R, Cassaundra Roback, Demi Sargent, Bridget K Murphy, Patrick J Hudson, and Christopher D Muir. 2021. “Principles of Resilient Coding for Plant Ecophysiologists.” *AoB PLANTS* 13 (5): plab059. https://doi.org/10.1093/aobpla/plab059.

Wilks, S. S. 1938. “The Large-Sample Distribution of the Likelihood Ratio for Testing Composite Hypotheses.” *The Annals of Mathematical Statistics* 9 (1): 60–62. https://doi.org/10.1214/aoms/1177732360.
